# Plant-derived mitochondria mitigate aging-related neurodegeneration by reprogramming microglial mitochondrial energy metabolism

**DOI:** 10.1186/s40035-026-00565-1

**Published:** 2026-07-08

**Authors:** Yun Teng, Chao Luo, Qingbo Xu, Jingyao Mu, Lucy Teng, Hongjia Qian, Yinan Huang, Minmin Liu, Lifeng Zhang, Juw Won Park, Jae Yeon Hwang, Maiying Kong, Jun Yan, Michael L. Merchant, Huang-Ge Zhang

**Affiliations:** 1 Department of Microbiology and Immunology, University of Louisville, Louisville, KY USA; 2 Brown Cancer Center, Department of Medicine, University of Louisville School of Medicine, CTRB Room 309, 505 S. Hancock Street, Louisville, KY 40202 USA; 3 Robley Rex Veterans Affairs Medical Center, Louisville, KY 40206 USA; 4 Department of Central Laboratory, The Affiliated Huai’an First People’s Hospital of Nanjing Medical University, Huai’an, 223300 Jiangsu China; 5 Department of Breast and Thyroid Surgery, The Affiliated Huai’an First People’s Hospital of Nanjing Medical University, Huai’an, 223300 Jiangsu China; 6 Massachusetts Institute of Technology, Cambridge, MA USA; 7 Department of Bioinformatics and Biostatistics, SPHIS, University of Louisville, Louisville, KY 40202 USA; 8 Kidney Disease Program and Clinical Proteomics Center, University of Louisville, Louisville, KY USA

**Keywords:** Plant mitochondria, Mitochondria transfer therapy, Aging-related neurodegeneration, Cross-kingdom mitochondrial fusion, Plant mitochondrial microRNAs, Microglia mitochondrial metabolism, NADH dehydrogenase (Complex I), Reverse electron transport (RET), Reactive oxygen species (ROS), Cardiolipin

## Abstract

**Background:**

Intercellular mitochondrial transfer is pivotal in both healthy and pathological states. Supplementing healthy mitochondria is emerging as a promising therapeutic approach for various diseases. Non-immunogenic edible plants, which contain mitochondria, offer a novel avenue for such therapies.

**Methods:**

Mitochondria were isolated from several commonly consumed edible plants (P-Mit) using differential centrifugation followed by sucrose gradient ultracentrifugation. The distribution of P-Mit, particularly in the brain, was examined with a mitochondrial membrane-potential dye and an imaging system. As a proof of concept, the molecular interactions underlying turmeric-derived mitochondria (T-Mit) uptake by microglia were elucidated through affinity precipitation coupled with mass spectrometry. By labeling with gold-nanoparticles in a distinct triangular or spherical shape followed by electron microscopy and energy dispersive spectroscopy analysis, we demonstrated the physical fusion of T-Mit and animal mitochondria in microglia. Mitochondrial functions such as superoxide levels, ATP-linked mitochondrial respiration, glycolysis and electron transport chain activity were assessed to determine the impact of T-Mit on aging-related microglial dysfunction. Next-generation small RNA sequencing revealed the underlying mechanism by which T-Mit-derived small RNAs modulate the expression of NADH dehydrogenase (ND) genes in microglia.

**Results:**

Orally administered T-Mit travelled from the gut to the brain in aged male mice, where they fused with microglial mitochondria (M-Mit), reprogramming M-Mit energy metabolism and reversing aging-related cognitive dysfunction. Specifically, T-Mit was taken up by microglia via the phagocytic receptor TREM2. Subsequently, T-Mit fused with M-Mit in a mitofusin 1-dependent manner. The T-Mit microRNAs Tae-miR319 and Osa-miR166a-3p then integrated into M-Mit, inhibiting the expression of complex I subunits ND4 and ND5. This inhibition alleviated reverse electron transport (RET) at complex I, reducing reactive oxygen species (ROS) production and facilitating ATP production, ultimately rescuing aging-related cognitive decline. Data from elderly human subjects also showed overactivation of the RET process and overproduction of ROS, accompanied by low ATP levels in microglia.

**Conclusions:**

Our findings fundamentally alter our understanding of the regulation of mammalian mitochondrial biology by P-Mit and may lead to P-Mit-based transfer therapy for preventing or treating human mitochondrial disorder-related diseases.

**Supplementary Information:**

The online version contains supplementary material available at 10.1186/s40035-026-00565-1.

## Introduction

Mitochondria are traditionally recognized as energy-producing centers in both mammalian and plant cells. However, recent studies suggest that mammalian cell mitochondria also serve as signaling organelles, participating in intracellular interactions and intercellular communication [1–4]. While many healthy diets come from plants, and plant cells also contain mitochondria, it remains unclear whether mitochondria from non-immunogenic edible plants can impact the health of consumers via regulating the activity of mammalian cell mitochondria.

Emerging evidence indicates that mammalian cell mitochondria can be transferred between cells [3, 5]. This intercellular mitochondrial transfer occurs through various mechanisms in vivo and plays a role in both normal physiological processes and disease pathogenesis [1, 3, 4, 6–8]. Importantly, this dynamic process allows damaged mitochondria to be replaced, thereby restoring cellular energy metabolism [5, 9, 10]. This phenomenon has been utilized as a foundation for developing mitochondria transfer therapy [4, 11]. Whether edible plant mitochondria (P-Mit) can positively influence mammalian cell mitochondria activity through such transfer remains an open question.

The electron transport chain (ETC) is a hallmark of mitochondrial function, serving as the primary source of ATP while also generating reactive oxygen species (ROS) that participate in cellular signaling but can cause oxidative damage if not properly regulated [12]. The ETC in the inner mitochondrial membrane consists of several complexes. Compared with other complexes such as complex II and complex III [13], the mammalian NADH-ubiquinone dehydrogenase (complex I) is the major entry point for ATP production via the NAD-driven forward electron transport (FET) process [14]. In this process, ubiquinone (CoQ) receives electrons from NADH, becoming ubiquinol (CoQH_2_) [15], facilitating the production of ATP in the process of FET. These electrons can flux back to complex I through CoQH_2_ via the reverse electron transport (RET) process, facilitating the production of ROS [16]. While RET can contribute to cellular signaling and adaptation, excessive RET is often associated with oxidative stress and mitochondrial dysfunction [14], leading to various pathologies, including neurodegenerative diseases and metabolic disorders [17]. In microglia from aged mice, a high CoQH_2_/CoQ ratio [18] at mitochondria complex I triggers the occurrence of RET leading to a burst of ROS [16, 18, 19]. The balance between RET and FET in mitochondria is crucial for maintaining cellular homeostasis and managing ROS and ATP production, as excessive ROS from RET can cause oxidative stress and damage [20, 21], while insufficient ROS can impair signaling pathways. The balance between RET and FET is influenced by factors such as substrate availability, oxygen levels, and the overall metabolic state of the cell [21, 22]. Intercellular mitochondrial transfer can potentially modulate this balance by introducing healthy mitochondria with optimal ubiquinone ratios into recipient cells, thereby improving their bioenergetic capacity and reducing oxidative stress [7, 10]. Therefore, targeting the complex I activity-related RET and ROS production provides a promising potential therapeutic strategy to benefit mitochondrial dysfunction-associated disease [16].

Mitochondrial dysfunction is implicated in neurodegenerative diseases [23]. Alzheimer’s disease (AD) mice treated with human mitochondria via intravenous administration show improved cognitive performance and reduced neuronal loss [24]. Inspired by these findings, in this study, we aimed to investigate whether edible plant mitochondria (P-Mit) from a healthy diet, known for their potential health benefits, can reverse age-related cognitive dysfunction by reprogramming mammalian cell mitochondrial activities, particularly via restoration of ATP production and inhibition of overproduction of ROS. We characterized the distribution of mitochondria isolated from select dietary plants, including turmeric, ginger, garlic and aloe in mice receiving gavage-given P-Mit. We further define the significance and molecular mechanisms of oral administration of turmeric-derived mitochondria (T-Mit), which leads to rescue of aging-related cognitive dysfunction by dynamically fusing with microglial mitochondria. Our findings provide a perspective for the treatment of mitochondrial dysfunction-related diseases through P-Mit transfer therapy.

## Materials and methods

### Mice

Specific-pathogen-free (SPF) male C57BL/6 mice were purchased from the Jackson Laboratory (Bar Harbor, ME) and were divided into young group (6–8 weeks old), mid-aged group (10–12 weeks old) and aged group (18–20 weeks old). All mice had free access to regular chow diet and drinking water, and were housed under SPF conditions. Oral gavage was administered to mice using a curved gavage needle (20 gauge). Animal care was given following the Institute for Laboratory Animal Research (ILAR) guidelines, and all animal experiments were conducted following protocols approved by the University Institutional Animal Care and Use Committee (IACUC, #24374). The mice were acclimated for at least 1 week before any experiments were conducted.

### Human brain

Human brain hypothalamus tissues from deceased young (30.88 ± 4.09 years old, *n* = 20) and old subjects (70.75 ± 4.59 years old, *n* = 20) from the traffic accidents without apparent traumatic brain injury were provided by Huai'an First People's Hospital, Huai'an, Jiangsu, China and Biochip Co. Lid (Shanghai China). Written informed consent for tissue collection and research use was obtained from a legally authorized individual empowered to make decisions on behalf of the deceased.

All brain tissues were collected from deceased individuals within 12 h of death. To investigate the alteration of microglial mitochondrial activity in aging without the estrogen interruption, this study only involved human brain tissues from men. Gender was determined based on karyotype analysis. Approval for the study was granted by the Institute Research Ethics Committee at the Health Department of Huai’an.

### Cells

C57BL/6 murine BV2 microglial cells, and U937 human macrophages were purchased from American Type Culture Collection (ATCC, Manassas, VA) and cultured in tissue culture plates with Dulbecco's modified Eagle's medium (DMEM) supplemented with 10% heat-inactivated fetal bovine serum (FBS), 100 U/mL penicillin, and 100 µg/mL streptomycin at 37 °C in a 5% CO_2_ atmosphere.

### Microglial isolation from mouse brain

To isolate microglia, transcardial perfusion was conducted via the inferior vena cava with perfusion buffer (Ca^2+^-Mg^2+^ free Hanks' Balanced Salt Solution [HBSS] containing 0.5 mM EGTA, 10 mM HEPES and 4.2 mM NaHCO_3_; pH 7.2). The brain tissue (< 400 mg) was homogenized and dissociated gently using the Neural Tissue Dissociation Kit (Miltenyi Biotec, Cat# 130–092-628, Auburn, CA). Briefly, the mouse brain tissue was incubated with 1950 µL of enzyme Mix at 37 °C for 15 min followed by transferring the mixture into the C Tube with enzyme mix 1. The brain cells were dissociated at 37 °C for 10 min using the Dissociator. The resuspended cell suspension was passed through a MACS SmartStraner. Microglia were isolated using Microglia MicroBeads (Miltenyi Biotec, Cat# 130-093-634), and then separated using autoMACS Pro Separator with a MACS MS Columns (Miltenyi Biotec, Cat# 130-042-201). The target microglial cells were eluted from the autoMACS Column as the positive fraction, using Running buffer.

### Isolation of mitochondria from plants and microglial cells

Plant and microglial mitochondria (P-Mit and M-Mit) were isolated using a previously described method [25, 26] with minor modifications. Turmeric roots (price look-up (PLU) code: 4918), Hawaiian ginger roots (Simply ginger, PLU#: 4612), garlic (PLU#: 3399), and aloe (PLU#: 3064) were purchased from a supermarket and peeled after disinfecting with ethyl alcohol (70%). The plants were then separately homogenized in a low-speed blender with homogenization buffer A (0.4 M sucrose, 1.0 mM EDTA, and 1% PVP-40) for 1–2 min. The juice was collected after net filtration and diluted with homogenization buffer. The filtered homogenate was then centrifuged for 10 min at 1000 × *g*. The washed organelles were further separated by density gradient centrifugation in sucrose (0.6 to 1.8 M sucrose in 10 mM Tri-Cl, pH 7.2, 1.0 mM EDTA, 0.1% bovine serum albumin [BSA]) at 12,000 × *g* for 30 min at 4 °C. After centrifugation, the mitochondria were collected from a band at ∼1.25 M to 1.35 M sucrose followed by washing three times with PBS [25].

To extract mitochondria from microglia in mice and BV2 cells, the isolated microglia were homogenized with a Teflon-glass homogenizer. The homogenate was centrifuged for 10 min at 1000 × *g* and the supernatants were collected. The pellets were washed with buffer B followed by a sucrose density gradient centrifugation as described above. The mitochondrial band was collected, washed and resuspended in wash buffer for further study. Purified mitochondria were visualized under a Zeiss EM 900 electron microscope using a previously described method [27].

### Purification of mitochondrial complex I

Mitochondrial complex I was extracted using a previously described method [28]. The isolated mitochondria were suspended in TE buffer (20 mM Tris-Cl, pH 7.5, 5 mM EDTA) and disrupted by a Dounce homogenizer. After centrifugation at 100,000 × *g* for 30 min at 4 °C, the pellets were suspended in 0.1% Triton X-100 and the lysates were centrifuged again at 100,000 × g for 30 min at 4 °C. The resulting supernatants were used for complex I isolation by sucrose gradient centrifugation (10%–25% in 20 mM Tris-HC1, pH 7.5, 5 mM EDTA, 150 mM NaCl, 0.1% Triton X-100).

### Biotinylation of protein

The protein was labeled with biotin using a Biotinylation Kit (Thermo Fisher, Cat# 20217, Branchburg, NJ) according to the manufacturer’s instructions. Briefly, 1 mg protein of mitochondrial complex I was incubated with 10 mg/mL of NHS-PEG4-biotin for 2 h at 4 °C. The biotinylated protein was purified by dialysis. The biotinylated proteins and their interacting complex were pulled-downed by the MagnaBind Streptavidin Beads (Cat# 21344, Thermo Fisher) using autoMACS Pro Separator with a MACS MS Columns (Miltenyi Biotec).

### Transmission electron microscopy (TEM)

Isolated mitochondria or murine brain tissue was fixed in 2% paraformaldehyde (Electron Microscopy Science, Cat# 15700, Morgantown, PA) in PBS for 2 h at 22 °C followed by 1% glutaraldehyde (Electron Microscopy Science, Cat# 111–30-8) for 30 min at 22 °C. The fixed samples (15 µL) or brain tissue sections (100 nm) were placed on 2% agarose with formvar/carbon-coated copper grids and allowed to absorb for 5–10 min. The grids with adherent mitochondria were fixed in 2% paraformaldehyde in PBS for 10 min followed by extensive washing in PBS. Negative contrast staining was performed with 1.9% methyl cellulose and 0.3% uranyl acetate for 10 min. The grids with negatively stained mitochondria were dried before observation under a Zeiss EM 900 electron microscope.

### Labeling mitochondria with gold nanoparticles for TEM

P-Mit and M-Mit were labeled with triangular and spherical gold-nanoparticles, respectively [29, 30]. To label P-Mit with the gold-triangle nanoparticles for TEM analysis, isolated P-Mit (1 mg/mL) in 1 × HBSS was incubated with 10 µg of N-hydroxysuccinimide-terminated gold-triangle nanoparticles (Nanopartz, Cat# C1T-625-TNHS-DRY-0.5, Loveland, CO) in pH 8.0 at 30 °C for 12 h. After centrifugation at 10,000 × *g* for 15 min, the P-Mit labeled with gold-triangle nanoparticles were washed three times with HBSS (pH = 7) and resuspended in 2.5% glutaraldehyde buffer in 0.1 M sodium cacodylate buffer pH 7.4 (Electron Microscopy Sciences, Cat#11654) overnight at 4 °C for TEM analysis or stored at − 20 °C for later experimental use.

To label mitochondria in microglia and mouse brain tissue in situ with the gold-sphere nanoparticles for TEM analysis, the microglial cells or brain tissue section was loaded on 2% agarose-formvar/carbon-coated copper grids for 30 min. The grids were washed three times with 0.02 M glycine in PBS, and then treated with a blocking solution of 1% BSA in phosphate-buffered saline (PBS). The grids were then floated for 1 h on droplets of anti-Tom20 antibody (1:200) (Proteintech, Cat# 11802, Rosemont, IL) in blocking solution. After a wash with 0.1% BSA in PBS, the grids were incubated for 30 min with protein A-conjugated 10-nm-diameter gold-sphere particles (Cytodiagnostics, Cat# G-100-20, Tulsa, OK) diluted 1:40 in blocking solution. After one wash in PBS, the grids were fixed with 1% glutaraldehyde, washed with distilled water, and then stained with 1% ammonium molybdate. After removal of excess ammonium molybdate by washing three times using PBS, samples were observed using a Thermo-Fisher TEM Tecnai Spirit at 80 kV, and images were collected with an AMT XR60 digital camera.

### Energy dispersive spectroscopy (EDS)

Isolated T-Mit were labeled with silica-coated gold nanoparticles (Nanopartz, Cat# C61T-600-10-TPA-DIH) using the same strategy for TEM described above. BV2 cells were incubated with the labeled T-Mit, then loaded on 2% agarose-formvar/carbon-coated copper grids. The BV2 cell mitochondria were labeled with gold nanoparticles through the interaction between anti-Tom20 and protein A-conjugated gold nanoparticles. The cells was loaded on 2% agarose-formvar/carbon-coated copper grids followed by fixing with glutaraldehyde. The imaging and elemental mapping to identify and quantify elements, particularly gold and silica within mitochondria, were conducted using TEM integrated with an energy-dispersive X-ray spectroscopy (EDS) detector (Thermo Fisher Apreo).

### In situ protein proximity ligation assay (PLA)

The protein proximity between animal mitochondrial membrane protein Mitofusin (MFN) 1 and plant mitochondrial membrane-specific protein VDAC1 [31] within mitochondria was assessed using the MolBoolean Mouse/Rabbit Assay Kit (Novus Biologicals, # MolB00001, Centennial, CO). Brain specimen were collected from T-Mit-administered aged mice and fixed with periodate-lysine-paraformaldehyde (PLP) followed by dehydrating with 30% sucrose in PBS at 4 °C overnight. The sections were blocked with 1 × Blocker for 1 h at 37 °C and incubated with anti-mouse MFN1 (Novus Biologicals, Cat# H00055669) and anti-plant VDAC1 (PhytoAB Inc., Cat# PHY0694S, San Jose, CA) sequentially in TBST diluted 1:100. After wash with TBST, the slides were incubated with Probe A and Probe B for 1 h at 37 °C. Circle Tag oligo was hybridized for 30 min and incubated with NIckase enzyme for 1 h. The slides were incubated with DNA ligase enzyme and polymerase enzyme, respectively, for rolling circle amplication. After incubation with detection oligos and nuclear conuter stain, The signal of fluorescence was visulized with a confocal microscope with appropriate spectra such as Probe A (MFN1) at 575–620 nm (Texas red filter) or Probe B (VDAC1) at 657–750 nm (Y5 filter). The relative number of PLA dots was then quantified using the ImageJ software.

### Extraction of mitochondrial lipids

To isolate lipids from purified mitochondria, one volume of mitochondria was mixed with 1.25 volumes of chloroform and 2.5 volumes of methanol in a glass tube and thoroughly vortexed. An additional 1.25 volumes of chloroform and 1.25 volumes of H_2_O were added and mixed well. The mixture was centrifuged at 2000 rpm for 10 min at room temperature and the lower organic phase removed and dispensed into a new clean glass tube [32]. The samples were dried under nitrogen (2 psi) and stored at − 80 °C.

### Lipidomic analysis with liquid chromatography-mass spectrometry (LC–MS)

Lipid samples extracted from mitochondria were submitted to the Lipidomics Research Center, Kansas State University (Manhattan, KS) for analysis. In brief, the lipid composition was determined using triple quadrupole MS (Applied Biosystems Q-TRAP, Applied Biosystems, Carlsbad, CA). The data are reported as the percentage of each lipid within the total signal for the molecular species determined after normalization to internal standards of the same lipid class.

### Preparation of nanovesicles (NVs) with T-Mit lipids (T-NV)

Briefly, 1 mg of mitochondrial lipids was dissolved in 2 mL of anhydrous ethanol. The lipid solution was filtered using a 0.45-µm syringe PES filter (Santa Cruz Biotechnology, Cat# sc-516779, Dallas, TX). Deionized water was filtered through a 0.45-µm syringe PES filter (Santa Cruz Biotechnology, Cat# sc-516779) to obtain the water solution. The NVs were generated using Liposome Maker (PreciGenome LLC, San Jose, CA) according to the manufacturer’s instructions.

### Preparation of turmeric-derived exosome-like nanoparticles

The same turmeric used for mitochondrial isolation was used for the isolation and purification of nanoparticles. Briefly, turmeric root was peeled after disinfecting with ethyl alcohol (70%) and then homogenized in a high-speed blender for 1 min. The juice was collected after net filtration and diluted with sterile PBS. The supernatant was collected after centrifugation at 1000 × *g* for 10 min, 2000 × *g* for 20 min, 4000 × *g* for 30 min, and 10,000 × *g* for 1 h. The pellets containing nanoparticles derived from each plant were spun down at 100,000 × *g* for 1.5 h at 4 °C and named as the exosome-like nanoparticles. The isolated nanoparticles were further purified by sucrose gradient (8%, 30%, 45%, and 60% sucrose in 20 mM Tri-Cl, pH 7.2) centrifugation at 100,000 × *g* for 1.5 h at 4 °C.

### Labeling of mitochondria with fluorescent dye

Plant mitochondria were labeled with Lipophilic Tracers including DiR (Emission, 780 nm, Cat# D12731), DiO (Emission, 501 nm, Cat# D3898), DiI (Emission, 565 nm, Cat# D3911) (Invitrogen, Eugene, OR), and mitochondrial membrane potential-based dye MitoTracker (Thermo Fisher, Cat# M7514, Cincinnati, OH). Intra-mitochondrial protein, RNA and DNA were labeled with ExoGlowTM-Protein/-RNA/-DNA (Thermo Fisher, Cat# EXOC300A) in accordance with the manufacturer's instructions. The stock solution of the tracers was prepared with dimethylsulfoxide at 1.0 mg/mL. After washing with PBS, mitochondria (10 mg) were suspended in 250–500 µL of PBS with 2–4 µL of stock solution and subsequently incubated for 30 min at room temperature. After centrifugation for 5 min at 13,000 × *g*, the labeled mitochondria were resuspended and washed three times with PBS.

### Image assessment of P-Mit distribution

P-Mit labeled with DiR was administered to mice via oral gavage and free dye DiR was used as negative control. Transcardial perfusion was conducted via the inferior vena cava with perfusion buffer (Ca^2+^-Mg^2+^free HBSS containing 0.5 mM EGTA, 10 mM HEPES and 4.2 mM NaHCO_3_; pH 7.2) prior to tissue collection. The fluorescence signal was collected using an Odyssey Imager (LiCor Inc, Lincoln, NE). The display values of images from different groups of animals and different tissues were linked by the image ID using the software Image Studio ver4.0 (LiCor Inc). This applies the values of a given channel to all images scanned at different time-points. The setting of image intensity was adjusted until the negative controls exhibited no significant signal. The intensity of the signals were provided for statistical analysis by the software.

### Integrity assessment of isolated plant mitochondria

The integrity of isolated mitochondria was assessed by oxidation of added cytochrome c using an Oxygen Consumption Rate Plate Assay Kit (Dojindo, Cat# VG661, Rockville, MD).

### Complex I enzyme activity assay

Complex I activity was assessed using a Complex I Enzyme Activity Microplate Assay Kit (Abcam, Cat# ab109721, Cambridge, UK) according to the manufacturer’s instructions. Briefly, 2 × 10^5^ cells or 1.0 mg isolated mitochondria were suspended in 0.5 mL PBS with 1/10 (*v*/*v*) detergent solution for 30 min at 4 °C. After centrifugation at 16,000 × *g* for 20 min at 4 °C, 100 µL of supernatant was collected and mixed with 100 µL of incubation solution in a 96-well microplate for 3 h at room temperature. The solution was removed, and the plate was washed with 300 µL of 1 × wash buffer solution and 200 µL of assay solution was added, followed by the measurement of the optical density (OD450 nm) with a BioTek microplate reader (Agilent, Santa Clara, CA).

### ATP synthase enzyme activity assay

ATP synthase activity was measured using a General ATP Synthase Microplate Assay Kit (MyBioSource, Cat# MBS8305380, San Diego, CA) according to the manufacturer’s instructions. 2 × 10^5^ cells or 1.0 mg isolated mitochondria were suspended in 1.0 mL Assay Buffer I containing 10 µL Assay Buffer II for 30 min at 4 °C. After centrifugation at 11,000 × *g* for 10 min at 4 °C, the pellet was suspended in 200 µL Assay Buffer III containing 2 µL Assay Buffer II. After centrifugation at 11,000 × g for 10 min at 4 °C, 50 µL of supernatant was mixed with Reaction Buffer and Substrate for 30 min at 37 °C. 50 µL Stop Solution was added and the mixture was centrifuged at 4000 × *g* for 10 min, followed by addition of 150 µL Dye Reagent and mixing. The enzyme-catalyzed reaction produces inorganic phosphate that reacts with the Dry Reagent at which point the optical density was measured at 660 nm using the BioTek microplate reader (Agilent).

### Measurement of ATP levels

The ATP levels were measured using an ATP Assay Kit-Luminescence (Dojindo, Cat# A550) according to the manufacturer’s instructions. First, a working solution was prepared by adding 10 µL of enzyme solution to 5.5 mL of assay buffer. Then, 2 × 10^5^ cells or 1.0 mg isolated mitochondria were incubated with 100 µL 1 × HBSS and 100 µL of Working solution for 10 min at 25 °C. Luminescence was measured using the BioTek microplate reader (Agilent), and the concentration of ATP in the samples was determined from the ATP standard curve.

### Mitochondrial membrane potential assay

Mitochondrial membrane potential was measured using a TMRE-Mitochondrial Membrane Potential Assay Kit (Abcam, Cat# ab113852) and JC1 MitoMP Detection kit (Dojindo, Cat# MT09-10) according to the manufacturer’s instructions. The oxidative phosphorylation uncoupler FCCP (20 µM) was added after 180 s and served as a depolarization control to exclude the false-positive potential.

### Mitochondrial reactive oxygen species (mtROS) detection

The mtROS was assess using MitoBright ROS Deep Red (Dojindo, Cat# MT16) according to the manufacturer’s instructions. Briefly, 2 × 10^5^ cells or 1.0 mg isolated mitochondria were plated in a 96-well black plate and incubated with 100 µL 1 × HBSS containing 10 µmol/L MitoBright ROS Deep Red at 37 °C for 30 min. After washing with 1 × HBSS, 100 µL HBSS containing Antimycin (stimulator, 10 µmol/L) was added to the plate at 37 °C for 15 min. Without washing, the fluorescent intensity (excitation at 535–565 nm and emission at 660–690 nm) was measured using a BioTek microplate reader (Agilent).

### Mitochondrial superoxide (mtSOX) detection

mtSOX was estimated using MitoSOX™ Mitochondrial Superoxide indicator (Invitrogen, Cat# M36008) according to the manufacturer’s instructions. 2 × 10^5^ cells or 1.0 mg isolated mitochondria were plated in a 96-well black plate and incubated with 100 µL 1 × Hanks' Balanced Salt Solution (HBSS) containing 500 nmol/L MitoSOX™ Mitochondrial Superoxide indicator at 37 °C for 30 min. After washing with 1 × HBSS, 100 µL HBSS containing Antimycin (10 µmol/L) was added to the plate at 37 °C for 15 min. Without washing, the fluorescent intensity (excitation at 396 nm and emission at 610 nm) was measured using a BioTek microplate reader (Agilent).

### Measurement of ROS in cerebrospinal fluid (CSF)

CSF was collected from the lateral ventricle through the foramina [33] and ROS level was assessed using a DCF ROS Assay Kit (Abcam, Cat# ab238535). The quenched fluorogenic probe (100 μL) was incubated with CSF (10 μL) at room temperature for 20 min and the ROS-oxidized highly fluorescent 2', 7'-dichlorodihydrofluorescein (DCF) was measured using a microplate reader (Agilent) with excitation at 480 nm and emission at 530 nm.

### Mitochondrial NAD^+^ /NADH ratio assay

Mitochondrial NAD^+^/NADH ratio was assessed using a NAD^+^/NADH Assay Kit (Sigma-Aldrich, Cat# MAK460, Burlington, MA) according to the manufacturer’s instructions. 2 × 10^5^ cells or 1.0 mg isolated mitochondria were suspended in 100 µL of NAD^+^ or NADH Extraction Buffer, respectively and incubated at 60 °C for 5 min. The reaction of extraction was neutralized with the opposite buffer and subsequently centrifuged at 14,000 × *g* for 10 min at 4 °C. Then 50 µL of supernatant was mixed with the same volume of fresh Working Reagent in a 96-well black plate and the fluorescent intensity (excitation: 530 nm and emission: 585 nm) was measured using a BioTek microplate reader (Agilent).

### Analysis of cell energy metabolism using Seahorse assay

To assess mitochondrial energy metabolism, both oxygen consumption rate and extracellular acidification rate were evaluated to monitor mitochondrial respiration and glycolysis in cells using selective substrates/inhibitors at different metabolic states. Seahorse XF analysis was performed at 37 °C simultaneously measuring oxygen consumption rate (pmole O_2_ /min) and extracellular acidification rate (mpH/min) using the XF-96 Extracellular Flux Analyzer (Agilent).

### Measurement of cardiolipin in mitochondria

Mitochondrial cardiolipin was assessed using Nonyl Acridine Orange (NAO) fluorescent dye (Thermo Fisher, Cat# A1372). Plant-derived mitochondria (1–2 mg) were incubated with Acridine Orange 10-Nonyl Bromide (10 ng/mL) at 37 °C for 15 min. Without washing, the fluorescent intensity (excitation: 495 nm and emission: 519 nm) was measured using a BioTek microplate reader (Agilent).

### Flow cytometry analysis

Microglia BV2 cells or brain cells isolated from mice were incubated with blocking buffer (2% BSA) and subsequently stained for 1 h or overnight at 4 °C with the appropriate fluorochrome-conjugated antibodies in PBS with 2% FBS. After fixdation/permeabilization using the Fixation/Permeabilization Buffer Set (eBioscience, Cat# 88-8824-00, San Diego, CA), cells were incubated with antibodies against CD-11b (Abcam, Cat# 11-0112-41), CD45 (Abcam, Cat# 11-7321-82), NeuN (Abcam, Cat# ab223994), MOG (Abcam, Cat# ab306602), GFAP (Abcam, Cat# ab223127), and p16Ink4a (LS Bio, Cat# LS-C691417, Seattle, WA). Data were acquired on a BD FACS CantoTM (BD Biosciences, Cat# 338962, San Diego, CA) and were analyzed using FlowJo™ software (v10.0, Tree Star Inc., Ashland, OR). Forward versus side scatter (FSC vs SSC) gating was used to identify cells of interest while excluding debris and dead cells. This was followed by gating signals in fluorescence channels such as PE and FITC for further analysis of cell markers or collection of cells.

### Western blotting

Proteins were extracted using the radioimmunoprecipitation assay (RIPA) buffer (Sigma, Cat# 20-188) with addition of protease and phosphatase inhibitors (Roche, Cat# 11836170001, Basel, Switzerland). Proteins were separated by 10% SDS-PAGE and transferred to polyvinylidene difluoride (PVDF) membranes (Bio-Rad Laboratories, Hercules, Inc., CA). Antibodies were purchased as follows: MFN1 (Novus Biologicals, Cat# NBP1-51841), MFN2 (Novus Biologicals, Cat# NBP2-66383), optic atrophy 1 (OPA1) (Novus Biologicals, Cat# NBP2-59770), glyceraldehyde 3-phosphate dehydrogenase (GAPDH) (Abcam, Cat# ab9657), MT-ND4 (Invitrogen, Cat# PA5-101697) and ND5 (Invitrogen, Cat# PA5-116791), IκBα (Cell Signaling, Cat# 4814, Danvers, MA), phospho-NF-κB p65 (Cell Signaling, Cat# 3031), Tom20 (ProteinTech, Cat# 11802). Plant antibodies included anti-isocitrate dehydrogenase (IDH) (Boca Scientific Inc, Cat# AS06-203A, Boca Raton, FL) and anti-patellin (PATL) (PhytoAB, Cat# PHY2663). The dilution of the primary antibodies was 1:500 to 1:1000. The secondary antibodies conjugated to Fluors Alex-488 (Invitrogen, Cat# A32723) or Alex-594 (Invitrogen, Cat# A11012) were used at a dilution of 1:5000 to 1:10,000. The bands were visualized on the Odyssey Imager (LiCor Inc) and band density was analyzed using ImageJ software (NIH).

### Histological analysis

For hematoxylin and eosin (H&E) staining, tissues were fixed with buffered 10% formalin solution (Thermo Fisher, Cat# SF93-20) overnight at 4 °C. Dehydration was achieved by immersion in a graded series of 70%, 80%, 95%, and 100% ethanol for 40 min each. Tissues were embedded in paraffin and subsequently cut into ultra-thin slices (5 µm) using a microtome. Tissues were deparaffinized by xylene (Thermo Fisher, Cat# 1330-20-7) and rehydrated by decreasing concentrations of ethanol and PBS. Tissue sections were stained with H&E and slides were scanned with an Aperio ScanScope. For frozen sections, tissues were fixed with periodate-lysine-paraformaldehyde (PLP) and dehydrated with 30% sucrose in PBS at 4 °C overnight. The sections were incubated overnight at 4 °C with anti-Iba1 (FujiFilm, #019-19741, Tokyo, Japan) and anti-Tom20 (ProteinTech, Cat# 11802) diluted 1:100. The signal was visualized with secondary antibodies conjugated to Fluors Alex-488 or Alex-594 and nuclei were stained with 4',6-diamidino- 2-phenylindole dihydrochloride (DAPI). The slides were scanned using an Aperio ScanScope or visualized by confocal laser scanning microscopy (Nikon, Melville, NY).

### Nissl body staining

Nissl bodies in neurons were stained using cresyl violet [34]. Paraffin sections were conventionally deparaffinized and rehydrated through 100% ethanol to distilled water. The slides were stained in 0.1% cresyl violet solution (Thermo Fisher, Cat# 405760025) at 37 °C for 20 min. After three washes with distilled water, the slides were dried at 50 °C followed by dehydration in 95% ethanol and xylene, respectively. The blue Nissl substances were visualized using a bright-field microscope (Olympus, × 400, Tokyo, Japan). The number of Nissl bodies at the same site in each group of mice was counted. The average thickness of the granular layer was measured at five random positions in each slice under the same field of view.

### CRISPR/Cas9 lentivirus production

The CRISPR/Cas9 knockout (KO) plasmids containing specific guide RNA sequences for Triggering receptor expressed on myeloid cells 2 (TREM2) (Santa Cruz Biotechnology, Cat# sc-429903), MFN1 (Santa Cruz Biotechnology, Cat# sc-426545) and MFN2 (Santa Cruz Biotechnology, Cat# sc-431291) were used. Control CRISPR/Cas9 Plasmid (Santa Cruz Biotechnology, Cat# sc-418922) was used as negative control. To assemble the lentivirus activation particles in cells, HEK293T cells (ATCC, Cat# CRL-11268) were transfected with the above Cas9 and sgRNA-containing plasmids, and packaging plasmid pCMV delta R8.2 and pCMV-VSV-G (Addgene, Cat# 12263 and 8454, Watertown, MA) in DMEM medium (with no antibiotics) containing 30 µg polyethylenimine (Polysciences, Cat# 23966, Warrington, PA) using FuGENE reagent. After 72 h of transfection, pseudovirus-containing culture medium was collected and the viral titer determined by performing BV2 cell transduction. The transduction efficiency and the *TREM2*, *MFN1* and *MFN2 *knockout efficiency of the virus particles were confirmed by western blot.

### Morris water maze (MWM) test

Mice were trained in a 122-cm diameter, 50-cm high open-field water maze with non-reflective interior surfaces. The pool water was maintained at 25 ± 1 °C and mixed with non-toxic white tempera paint. Distinctive and extra-maze cues were marked on the wall at specific locations and were visible to the mice in the maze. Mice were placed in the water from quasi-random start points and allowed a maximum of 60 s to probe the escape platform. The interval between trials was 15 s. For four consecutive days, four trials (training) were recorded each day with a final test on day 5. The performance was recorded with a camera and evaluated using the ANY-Maze software (Stoelting Co, Wood Dale, IL) based on every training trial and a final test on day 5 [35]. Performance in the MWM was evaluated by the mean escape latencies based on every training trial [35]. The spatial learning capacity of the mice was then evaluated by the relative distance traveled (path length/pool diameter). To minimize potential age-related physical bias, improvements in latency time during the training process were analyzed as indicators of learning and memory, while path lengths in the probe tests were normalized to each mouse’s initiate trial path length. 

### Novel object recognition (NOR) test

During the habituation phase, mice were allowed free exploration of a 40 × 40 cm^2^ open-field box for 5 min. The arena was thoroughly cleaned with 70% (*v*/*v*) ethanol between mouse use. On the first training run, two identical objects were placed in opposite quadrants of the box and the mice were allowed to familiarize themselves with the objects for 10 min. After 24 h, one of the objects was replaced by a novel one of different color and shape but in the same location. Each mouse was placed back in the box. The time mice spent in exploring the familiar and the new objects was recorded over a 10-min period. After four consecutive days, the final test was performed on day 5. Mouse performance was recorded using the ANY-Maze software (Stoelting Co). The discrimination index was calculated by comparing the exploration time on the new object to the total object exploration time to assess recognition performances [36].

### T-maze test

Modified T-maze testing was performed to evaluate cognitive impairment without the requirement of full motor function [37]. Mice were gently handled, habituated to the T-maze apparatus, and subjected to food deprivation for 24 h. In the forced alternation sample trial (T1), each animal was placed in the T-maze with one of the arms opened, with a pellet at the end of the arm as a reward. The opposite arm was blocked by a guillotine door. The mouse was allowed to explore the open arm and consume the reward. After 10 consecutive trials, the mouse was removed. After 30 min, retrieval testing (T2) was performed. The blocked arm was opened and the mouse was placed in the same start position. If the mouse entered the previous open arm with a reward, the response was recorded as “correct” and the animal would receive a reward. Each mouse was subjected to 10 consecutive runs. After four consecutive days, the final test was performed on day 5. Mouse performance was recorded using the ANY-Maze software (Stoelting Co). Forced Alternation (%) was defined as the percentage of mice first entering the “correct” arm during T2.

### Surface plasmon resonance (SPR) analysis

To identify whether the binding of TREM2 with T-Mit occurred in a lipid-dependent manner, SPR experiments were conducted on the OpenSPR™ platform (Nicoya Lifesciences, Ontario, Canada). Experiments were performed on a streptavidin sensor (Nicoya Lifesciences). SPR was run at a flow rate of 20 µL/min using the HBS running buffer (20 mM HEPES, 150 mM NaCl, pH 7.4). First, the streptavidin sensor chip was cleaned with octyl β-D-glucopyranoside (40 mM) and CHAPS (20 mM). Then 200 µL of biotin-conjugated recombinant TREM2 protein (1 µg/mL) was injected into the sensor chip for 10 min until a stable resonance was obtained. After immobilization of bio-TREM2, the surface was blocked with BSA (3%) in a running buffer. After a stable signal was obtained, T-Mit-derived T-NV with/without lipid phosphatidylserine (PS) depletion (10 nM) was run over the immobilized bio-TREM2 via the flow until a stable resonance was obtained. A negative control test was also performed by injecting phosphatidylglycerol (PG) onto a blank sensor chip to check for non-specific binding. The sensograms were analyzed using TraceDrawer kinetic analysis software.

### High-performance liquid chromatography (HPLC)

The reduced and the oxidized coenzyme Q (CoQH_2_, CoQ) as well as curcumin were assessed by HPLC. Samples were diluted with an equal volume of methanol. After centrifugation at 10,000 × *g* for 30 min, 50 μL of supernatant was injected for HPLC analysis. The HPLC analysis was performed on an Agilent 1260 Infinity system equipped with an Agilent ZORBAX SB-C18 column (4.6 × 150 mm, 3.5 μm), with the following parameters: mobile phase A: 5 mM NH_4_Ac in water modified with 0.1% formic acid (*v/v*); mobile phase B: 5 mM NH_4_Ac in 90% acetonitrile modified with 0.1% formic acid (*v/v*); gradient: 5% B in first 5 min, 5%–20% B for 10 min, hold 20% B for 5 min, 20%–50% B for 5 min, hold 50% B for 5 min, 50%–100% B for 5 min, hold 100% B for 10 min, 100%–5% B for 5 min; flow rate: 1.0 mL/min; temperature: 30 °C. The standards of CoQH_2_ (Cat# 1705334), CoQ (Cat# C9538) and curcumin (Cat C1386) were purchased from Sigma.

### Total RNA extraction

Total RNA containing small RNA was isolated from P-Mit and murine tissues using a miRNeasy mini kit (Qiagen, Cat# 1038703, Hilden, Germany) according to the manufacturer's instructions. In brief, 50 mg of P-Mit was lysed in 100 µL of cetyltrimethylammonium bromide (CTAB) buffer (2% CTAB, 2% polyvinylpyrrolidone, 2.0 M NaCl, 20 mM EDTA, 100 mM Tris–HCL (pH 8.0) and 5% ß-mercaptoethanol) at 60 °C for 30 min prior to mixing with 700 µL of QIAzol Lysis Reagent, which facilities P-Mit lysis [38]. The mammalian tissue was homogenized using a tissue grinder and lysed directly in QIAzol Lysis Reagent. The homogenate was mixed with 140 μL of chloroform and centrifuged. The upper aqueous phase was mixed with 1.5 volumes of ethanol and loaded into a RNeasy spin column. The flow-through was discarded after centrifugation, and the column was washed with RWT and RPE sequentially. Total RNA was eluted with 50 μL RNase-free water. The quality and quantity of the isolated RNA were analyzed using a NanoDrop spectrophotometer and Agilent Bioanalyzer.

### Next-generation small RNA sequencing

To identify the microRNAs (miRNAs) in T-Mit, small RNA libraries were generated with 100 ng of total RNA isolated from T-Mit, using TruSeq Small RNA Library Preparation Kits (Illumina, Cat# RS-200-0012, San Diego, CA) according to the manufacturer’s instructions. Following PCR amplification (16 cycles), libraries between 140 and 160 bp in size were gel purified and resuspended in 11 μL of ultrapure water. Equal amounts of libraries were pooled and sequenced on the Illumina HiSeq 2500, followed by demultiplexing and fastq generation with CASAVA v1.8.4. Raw fastqs were adapter- and quality score-trimmed with cutadapt v1.10, resulting in a minimum length of 15 nt. MiRNAs were identified using sRNABench Pipeline software (version 05/14) [39]. A core set of plant miRNAs from miRBase v21 were used. Within the sRNABench pipeline, mapping was performed with bowtie software (v0.12.9), and miRNA folding was predicted with RNAfold from the Vienna package (v2.1.6).

### Quantitative real-time polymerase chain reaction (qPCR)

For analysis of gene mRNA expression, 1 µg of total RNA was reverse transcribed using SuperScript III reverse transcriptase (Invitrogen, Cat# 11–732-020). Gene amplification and quantitation were performed using a QuantiTect SYBR Green PCR Kit (Qiagen, Cat# 204145). Primers are provided in Table S5. qPCR was performed using a BioRad CFX96 qPCR System with each reaction run in triplicate. Analysis and fold-changes were determined using the comparative threshold cycle (Ct) method. Changes in mRNA expression were normalized by GAPDH.

### Measurement of aspartate aminotransferase (AST) and alanine aminotransferase (ALT)

AST and ALT were measured using AST and ALT Activity Assay Kits according to the manufacturer's instructions (Abcam, Ab105135 and Ab105134). Briefly, 50 µL of serum was incubated with 100 µL prepared Reaction Mix at 37 °C for 1 h. The optical density (OD450: AST; OD570:ALT) was measured using the BioTek microplate reader (Agilent).

### Collection of gastric guice from mice

Mice were fasted for at least 6 h with free access to water prior to sample collection. After euthanasia with a mixture of ketamine (80 mg/kg) and xylazine (12 mg/kg) administered via intraperitoneal injection, the stomach was immediately excised, and gastric juice was aspirated using a syringe [40]. Under these conditions without stimulation, the yield of gastric fluid in our laboratory was approximately 50–200 µL per mouse. The average pH was 2.8 ± 1.2. To further reduce inter-animal variability, gastric juice collected from multiple mice was pooled, centrifuged, and the supernatant was aliquoted in equal volumes for each experimental group.

### Enzyme-linked immunosorbent assay (ELISA)

The cytokines in microglial cells were quantified using ELISA kits (eBioscience, Cat# 4201–56) according to the manufacturer’s instructions. Briefly, a microtiter plate was coated with anti-mouse tumor necrosis factor (TNF)α, interleukin (IL)-10, IL-12, transforming growth factor beta (TGF-β), inducible nitric oxide synthase (iNOS), CD206 and mouse immunoglobulin G (lgG) capture antibody (eBioscience, Cat# 18-4015-82) at 1:200 at 4 °C overnight. Excess binding sites were blocked with 200 µL of 1 × ELISA/ELISPoT Diluent (eBioscience, Cat# 4202-55) for 1 h at 22 °C. After washing three times with PBS containing 0.05% Tween 20, the plate was incubated with detective antibody in blocking buffer for 1 h at 22 °C. After washing three times, avidin conjugated with horseradish peroxidase and substrate were each added sequentially for 1 h and 30 min at 22 °C, respectively. Absorbance at 405 nm was recorded using a microplate reader (BioTek Synergy HT).

### Liquid chromatography with tandem mass spectrometry (LC–MS-MS)

Protein aliquots (50 μg) diluted in 4% SDS/0.1 M Tris–HCl (pH8.5) containing 1 M DTT was processed according to the filter-aided sample preparation (FASP) method as described [41, 42]. The digested, ultra-filtered samples were trap-cleaned with C18 PROTO™, 300 Å Ultra MicroSpin columns, lyophilized by vacuum centrifugation, and re-dissolved into 16 µL of 2% *v/v* acetonitrile. Concentrations were estimated based on absorption at 205 nm by Nanodrop 2000 (Thermo Fisher). Peptide samples were loaded onto an inhouse pulled (360 µm OD × 100 µm ID) fused silica tip needle tip packed with 12 cm of Aeris Peptide XB-C18 (3.6 µm, 100 Å) (Phenomenex, Cat# 00G-4507-E0, Torrance, CA) using a Proxeon EASY n-LC (Thermo Fishe, Cat# LC120) UHPLC system. Peptides were eluted using a linear gradient of 2% *v/v* acetonitrile/0.1% *v/v* formic acid to 40% *v/v* acetonitrile/0.1% *v/v* formic acid over 80 min. The sample was introduced into an LTQ-Orbitrap ELITE (Thermo Fisher) using a Nanospray Flex source with the ion transfer capillary temperature of the mass spectrometer set at 225 °C. The spray voltage was set at 1.6 kV. Data were acquired with an approach known as Nth Order Double Play created in Xcalibur v2.2. Scan event 1 of the method acquired an FTMS MS1 scan (normal mass range, 240,000 resolution, full scan mode, positive polarity, and profile data type) over an m/z range of 300–2000. Scan event 2 acquired ITMS MS2 scans (normal mass range, rapid scan rate, and centroid data type) for up to 20 peaks with a minimum signal threshold of 5000 counts selected from scan event 1. The lock mass option was enabled (0% lock mass abundance) using the 371.1012 m/z polysiloxane peak as an internal calibrant standard. Proteome Discoverer v1.4.1.114 (Thermo Fisher) was used to analyze the data collected by the mass spectrometer. The database was used in Mascot v2.5.1 and SequestHT. Scaffold was used to calculate the false discovery rate using the Peptide and Protein Prophet algorithms.

### Quantification and statistical analysis

Unless otherwise indicated, all statistical analyses were performed using SPSS 16.0 software. Continuous outcomes are presented as means ± standard deviations (SD). Comparisons between two groups were conducted using Student’s *t*-test, while comparisons among multiple groups were performed using one-way ANOVA, followed by post-hoc *t*-tests for selected pairwise comparisons. Categorical outcomes were analyzed using chi-square tests. *P* < 0.05 was considered as statistically significant.

Both animals and human subjects were randomly assigned to different experimental groups, with stratified randomization by age and sex. Double-blind procedures were implemented in both animal and human subject studies. Unless otherwise indicated, the mice used in the study were male C57BL/6 J mice. *n* = 10 mice for behavioral tests and *n* = 5 mice for non-behavioral assays [43]. Using a two-sample *t*-test, these sample sizes provide 80% power at a two-sided significance level of 0.05 to detect effect sizes of 1.32 (*n* = 10) and 2.02 (*n* = 5), respectively.

## Results

### P-Mit traffic from the gut into the brain in mice in an age-dependent manner

**Fig. 1:**
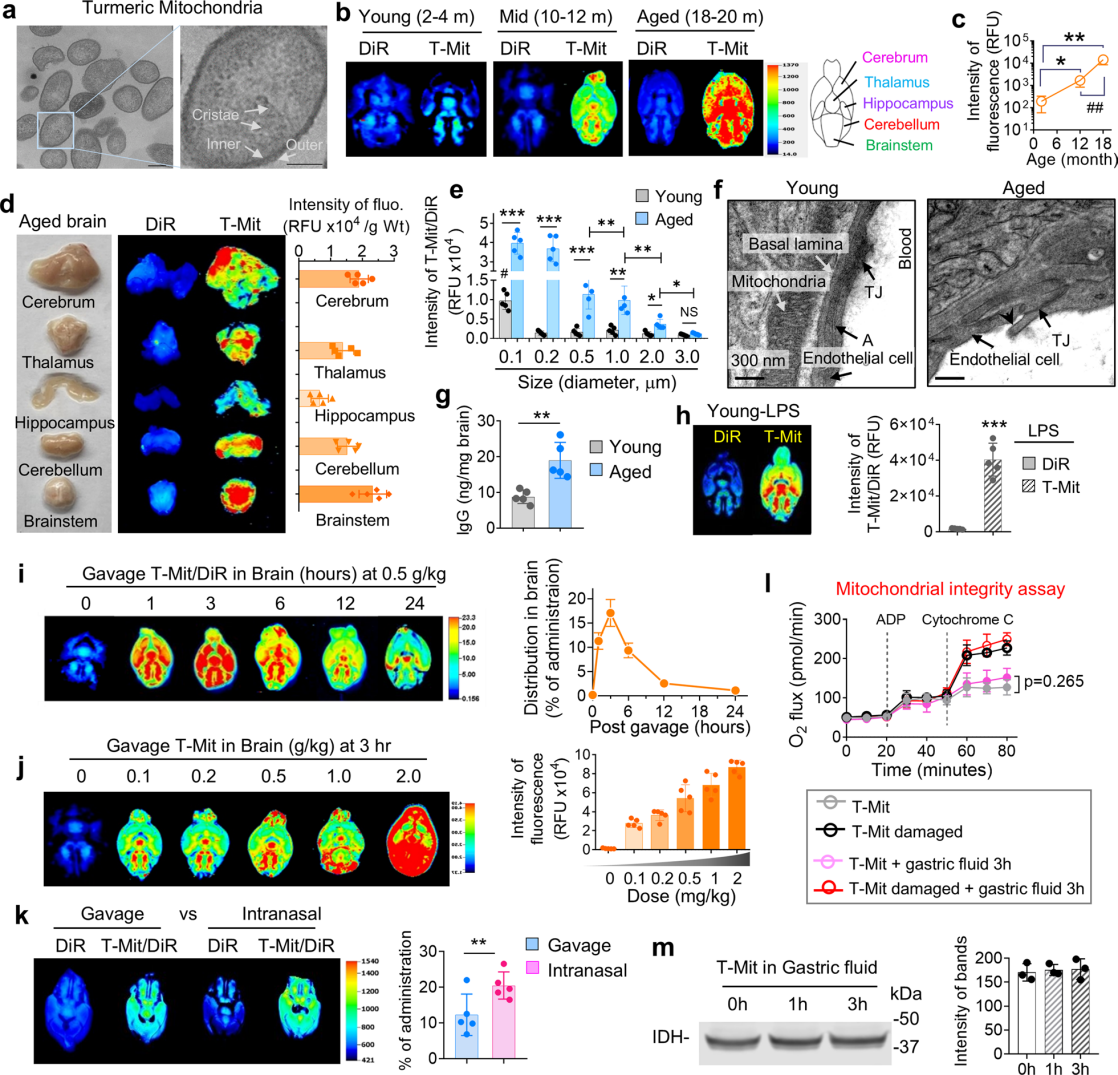
Turmeric-derived mitochondria (T-Mit) traffic from gut to brain in an aging-dependent manner. **a** A representative transmission electron microscopy (TEM) image of mitochondria isolated from turmeric (T‑Mit). Scale bars, 200 nm (left) and 100 nm (right). Arrows indicate the outer membrane, inner membrane, and cristae. **b** Left, representative fluorescent images of the brains from young (2–4 months), middle-aged (Mid, 10–12 months) and aged (18–20 months) C57BL/6 mice (*n* = 5) administered with a single gavage of 10 mg DiR dye-labeled T-Mit for 2 h. Mice with the same age fed with DiR free dye were used as a control. Right, a schematic of the brain major regions in mice. **c** Quantification of T-Mit/DiR fluorescence intensity in (**b**). *P < 0.05, **P < 0.01 (vs young); ^##^*P* < 0.01 (vs Mid) (one-way ANOVA with post-hoc *t*-tests). **d** A representative picture (left panel) and image of the cerebrum, thalamus, hippocampus, cerebellum and brainstem in the brains from the aged mice in (**b**) (middle panel); quantification of fluorescent density (right panel). **e** Permeability of fluorescent beads ranging in size from 100 nm to 3.0 µm, quantified as relative fluorescence units (RFUs) per gram of tissue. **f** Representative transmission electron microscopy (TEM) images showing tight junctions (TJ) and adherens junctions (AJ) (arrows) between two endothelial cells in the cerebral cortex of young (left) and aged (right) mice. Arrowhead indicates TJ disruption. Magnification, 11,000 × ; scale bars, 300 nm. **g** ELISA quantification of IgG levels in brain tissues from young and aged mice. **h** Representative fluorescence images of brains of young C57BL/6 mice (*n* = 5) receiving two intracranial injections of lipopolysaccharide (LPS) (100 µg) within a 12‑h interval, followed by a single gavage of 10 mg DiR dye‑labeled P‑Mit for 2 h. Quantification of T‑Mit–associated DiR fluorescence intensity in brain tissue is shown in the right. **i** Fluorescence images of aged mouse brains at different time points post T-Mit/DiR gavage (left panel, *n* = 5), and quantification of relative fluorescent intensity compared to the total administered T-Mit dose (right panel). **j** Representative fluorescence images of aged mouse brains at 3 h post T-Mit/DiR gavage at different doses (left panel, *n* = 5), and quantification of fluorescent intensity (right panel). **k** Representative fluorescence images of aged mouse brains at 2 h post oral and intranasal administration (1 mg), and quantification of fluorescent intensity compared to the total administered T-Mit dose. **l** Integrity of P-Mit (0.1 g) suspended in 0.1 mL gastric juice collected from aged mice for 3 h at 37 °C, assessed by O_2_ flux. **m** Western blot analysis of IDH protein in T-Mit at 3 h post gastric juice incubation, and quantification. Data from three independent experiments are presented as mean ± SD. * *P* < 0.05, ** *P* < 0.01, one-way ANOVA followed by post-hoc *t*-tests for continuous variables

Mitochondrial transfer or transplantation provides a promising strategy for the treatment of mitochondrial dysfunction-related diseases [44, 45]. This raises the attractive possibility that oral administration of non-immunogenic edible P-Mit may be used for the treatment of mitochondrial dysfunction-related diseases. As a proof of concept, mitochondria were isolated from several daily consumed edible plants, such as turmeric (T-), ginger (Gin-), garlic (Gar-) and aloe (A-), due to their beneficial effect on the health of humans [46–49]. These P-Mits were isolated by differential centrifugation and purified by sucrose gradient ultracentrifugation [50] (Fig. S1a). First, we analyzed their integrity, which is critical for their function and role in various cellular processes. TEM images (Fig. 1a and Fig. S1b) indicated that the average P-Mit size was 596.84 ± 68.72 nm in width and 1008.42 ± 284.51 nm in length, and BV2 microglial mammalian mitochondria were 615.33 ± 87.18 nm in width and 1256.08 ± 210.52 nm in length (Fig. S1b). In addition, the P-Mit exhibited a similar shape as mammalian mitochondria (Fig. S1b). Comparing the ratio of length to width, the aspect ratio distribution suggested that P-Mit tended to be more spherical and grain-shaped compared to M-Mit (Fig. S1c) [51]. The yield and the concentration of P-Mit in the tissue were quantitatively analyzed by laser-based dynamic light scattering analysis (Fig. S1d). The density-relative refractive index (RI) (Fig. S1e) and the surface Zeta potential (Fig. S1f) of P-Mit were assessed using a refractometer and ZetaView Nanoparticle Tracking Analyzer, respectively. The T-Mit had the highest RI while the A-Mit had the lowest RI (Fig. S1e). The ZetaView data suggested that the surface of the different P-Mits carries a negative charge between -15 to -35 mV (Fig. S1f).

ATP generation is a hallmark of mitochondrial functionality which is essential for cellular health and energy production. Mitochondria generate 95% of the cellular ATP through oxidative phosphorylation (OXPHOS) and the tricarboxylic acid cycle [9, 52]. We found that the Gar-Mit had the highest while the A-Mit had the least ATP product among the four P-Mits (Fig. S1g). The purity of the isolated P-Mit was estimated using western blot analysis with anti-plant specific antibodies. The data suggested that P-Mit were enriched with the mitochondrial-specific protein IDH but lacked the cytoplasmic protein PATL (Fig. S1h), indicating high purity of the isolated P-Mit without visible cytoplasmic contamination. Because cytochrome c increases mitochondrial oxygen flux when mitochondrial membrane integrity is compromised, the oxygen flux of P-Mit exposed to cytochrome c was measured to assess the integrity of the isolated P-Mit [53]. The results showed that exogenous cytochrome c did not significantly induce oxygen flux of any of the four P-Mits tested, suggesting unchanged membrane integrity of the isolated P-Mit [54] (Fig. S1i). As a control, P-Mit was pretreated with 1% Triton X-100 and the oxygen flux was elevated by cytochrome c, indicating damage of the outer mitochondrial membrane (Fig. S1i). Moreover, mitochondrial membrane potential was tested using the JC-1 dye as an additional independent way to test P-Mit purity and integrity [55]. The P-Mit exhibited a high ratio of red fluoresecence at 590 nm (polarized and functional mitochondria) to green fluoresecence at 527 nm (depolarized and impaired mitochondria), which was markedly shifted by the mitochondrial uncoupler carbonyl cyanide p-trifluoromethoxyphenylhydrazone (FCCP) [56] (Fig. S1j). Mitochondrial membrane potential of P-Mit was abolished by boiling or FCCP [56] (Fig. S1k). In addition, the mitochondrial-specific phospholipid cardiolipin [57] was assessed using the NAO fluorescent dye, and results showed that P-Mit contained high levels of cardiolipin in a membrane potential-dependent manner, as NAO fluorescence was also extinguished by FCCP (Fig. S1l). These data suggested that the isolated P-Mit had intact membrane potential, integrity and high purity.

Next, the distribution of P-Mit after oral administration was investigated. T-Mit was labeled with fluorescence DiR dye and administered via gavage (0.5 g/kg body weight) in 6–8-week-old (Young group), 10–12-month (Mid-aged group) and 18–20-month (Aged group) C57BL/6 mice. Brain distribution of T-Mit/DiR was assessed using an in vivo imaging assay at 2 h post-gavage. As expected, very little of T-Mit/DiR was evident in the brains of young mice (Fig. 1b). However, interestingly, a detectable fluorescent signal of T-Mit/DiR appeared in the brains of mid-aged mice, while the aged mice exhibited the strongest fluorescent signal of T-Mit/DiR in the brain (Fig. 1b, c). As a negative control, an equal amount of free DiR dye was administered but not detected in the brain (Fig. 1b). Moreover, of the five major separate regions of the brain analyzed, the hippocampus had the least fluorescent intensity of T-Mit/DiR (Fig. 1d). The data generated from T-Mit in aged mice were reproduced using mitochondria isolated from aloe, ginger and garlic (Fig. S2a). To exclude the possibility that the inaccessibility of P-Mit to young brain was attributed to the impact of gut barrier permeability, P-Mit was administered via an intravenous (i.v.) injection to young mice. As with the gavage method, very little signal of P-Mit was detected in the brains of young mice (Fig. S2a), while the majority of T-Mit accumulated in the liver and lungs (Fig. S2a, b). This result suggested that the accessibility of P-Mit to the brain in aged mice was not dependent on or related to gut barrier permeability. The distribution of P-Mit also suggested that regardless of the age of mice, all P-Mit could traffic to the liver and lungs via oral gavage in young and aged mice, while no evidence showed P-Mit trafficking to the spleen (Fig. S2a, b). However, quantitatively, compared to the young mice, aged mice had more T-Mit and A-Mit trafficking to the lungs, and more Gin-Mit trafficking to the liver and lung (Fig. S2a, b).

Considering that the blood–brain-barrier (BBB) plays an essential role in molecular trafficking to the brain, we next investigated whether BBB permeability was altered in the aged mice. Analysis of BBB permeability using liposomes of different sizes (Encapsula Nano Sciences, Cat# FPC-605, Brentwood, TN) via gavage suggested that the permeability through the BBB was size-limited and age-dependent, with permeability for liposomes below 200 nm in young and 3.0 µm in aged mice (Fig. 1e). Tight junction (TJ) structure was further investigated using freeze-fracture electron microscopy. We found disorganization of physical strand and interruptions of TJ between two endothelial cells in the cerebellum of aged mice (Fig. 1f). This interruption in TJ structure explains the BBB permeability dysfunction in the aged mouse brain. ELISA result revealed significantly increased IgG levels in aged mice, indicating leakage of circulating IgG into the brain parenchyma due to BBB disruption (Fig. 1g). Disruption of the BBB of young mice by intracranial injection of lipopolysaccharide [58] promoted P-Mit trafficking into the brain (Fig. 1h), suggesting that the BBB barrier function restricts P-Mit trafficking to the brain. Aged mice have an attenuated BBB barrier function [59, 60], which allows trafficking of more P-Mit to the brains of aged mice. Analysis of images of T-Mit/DiR in the peripheral blood of mice after gavage with T-Mit/DiR showed no difference in the intensity of fluorescence between aged and young groups, suggesting that the gut permeability to T-Mit did not affect the signal intensity of T-Mit/DiR circulating in the blood (Fig. S3a).

Next, we examined whether the signal of T-Mit/DiR in the brain is time-course- and dose-dependent. Our data showed that after gavage, T-Mit/DiR was detectable in the brains within 1 h, reached a peak at 3 h, and subsequently declined (Fig. 1i). Quantification of T-Mit trafficking from the gut to the brain revealed that up to 15% of T-Mit successfully reached the brain (Fig. 1i). Moreover, the brain signal intensity exhibited a clear dose-dependent relationship: higher gavaged doses of T-Mit/DiR resulted in stronger brain signals (Fig. 1j), with no significant differences observed between male and female mice (Fig. S3b). The absence of free DiR signal in the brain confirmed that the observed fluorescence originated from intact T-Mit rather than nonspecific dye leakage, thereby validating the integrity of T-Mit trafficking.

For therapeutic purposes, intranasal administration is another non-invasive strategy for delivering therapeutic agents to brain [61]. Intranasal administration of T-Mit (0.01 g/kg body weight) showed a higher efficiency of trafficking into the murine brain compared to gavage administration at the same dose **(**Fig. 1k**).** However, the volume of T-Mit was limited to 3–5 μL in a single intranasal administration. Therefore, the gavage-given route was selected for this study. We tested the impact of gastric fluid on the T-Mit integrity. Murine gastric fluid (pH 2.8 ± 1.2) was collected from the stomach of aged mice and incubated with T-Mit for 3 h at 37 °C. The results suggest that gastric fluid had no effect on T-Mit integrity (Fig. 1l) and the level of T-Mit protein IDH (Fig. 1m).

### T-Mit rescues the cognitive dysfunction in aged mice

**Fig. 2:**
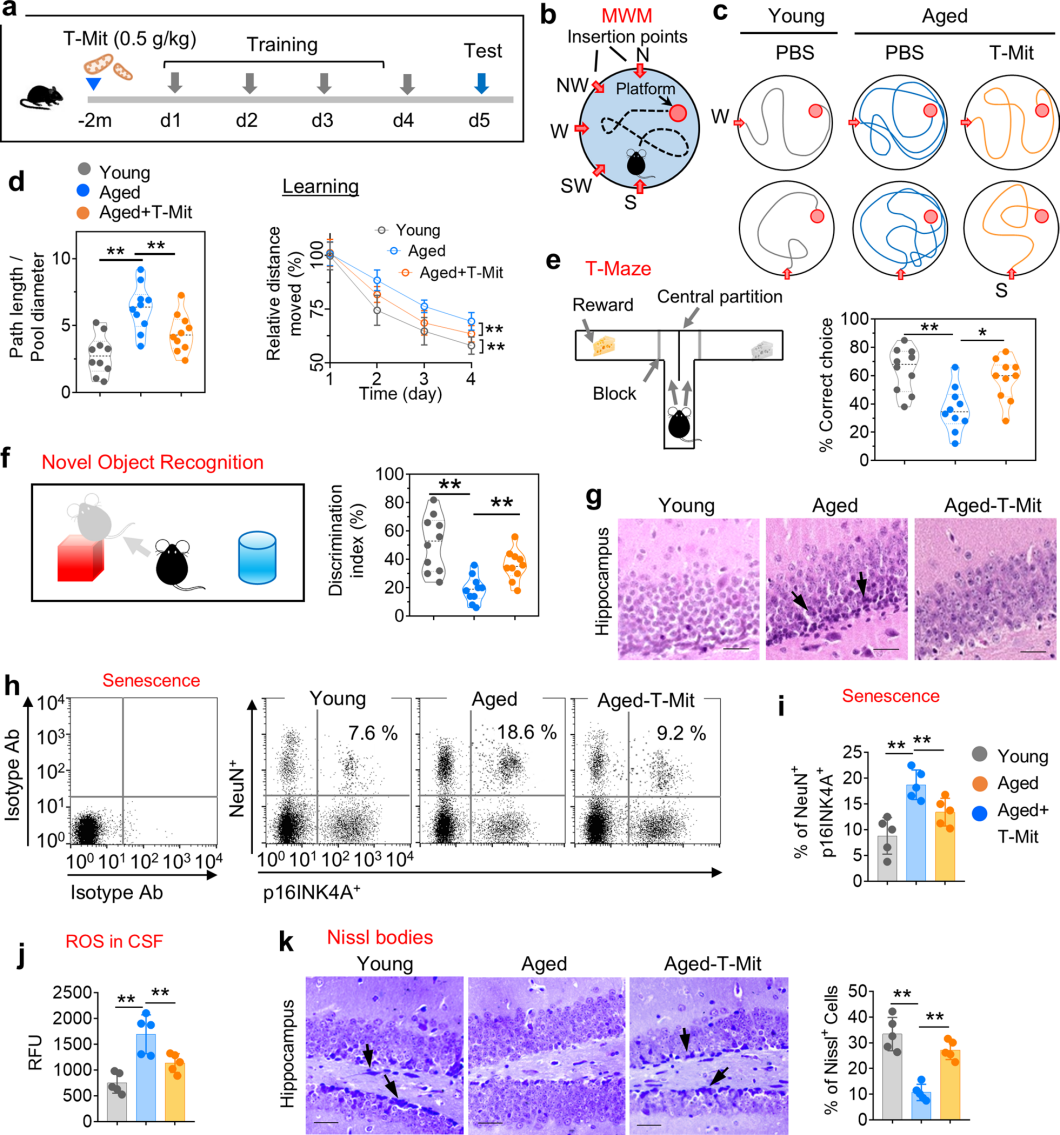
T-Mit rescues aging-related cognitive dysfunction and neural damage. **a** Schematic diagram of the treatment schedule. **b** Schematic diagram of the Morris water maze (MWM) test showing the platform and the variety of insertion points (S, south; SW, southwest; W, west; NW, northwest; N, north). **c** Representative search paths taken by mice on the day of the test at the insertion point of west (W, top panel) and south (S, bottom panel). **d** MWM performance of mice in the final test on day 5 (left panel) and in trials (days 1–4) (right panel) (*n* = 10 per group). **e** Schematic diagram of the T-maze Alternation test, and quantification of correct T-maze alternation. **f** Schematic diagram of the 2-Object Novel Object Recognition test (left panel), and quantification of the mean time spent interacting with each object (right panel). **g** H&E staining of sections of the hippocampus (scale bars, 200 µm). Arrows indicate neuronal loss and dying neurons (*n* = 5 per group). **h** FACS analysis of p16INK4A^+^NeuN^+^ cells in the brain. **i** Quantification of p16INK4A^+^NeuN^+^ cells in the FACS assay. **j** Analysis of mtROS in CSF of mice. RFU, relative fluorescence units. **k** Representative Nissl staining in mouse hippocampal sections and quantification of Nissl^+^ cells. Scale bars, 50 µm. Arrows indicate neuronal Nissl bodies (*n* = 5 per group). Data from three independent experiments are presented as mean ± SD. *P < 0.05, **P < 0.01, Chi-square test for categorical data; one-way ANOVA followed by post-hoc *t*-tests for continuous variables

Considering the capacity of P-Mit to traffic to the brain in an age-dependent manner and that turmeric root has been reported to be beneficial for brain health due to its antioxidant activity [62], we next determined whether T-Mit is beneficial for brain health in aging-related neurodegeneration. Aged mice were pretreated with T-Mit at 0.5 g/kg body weight every other day via oral gavage for two months, and then memory and learning was evaluated by MWM, T-maze alternation (TMSA) and two-object NOR (Fig. 2a). All animals were acclimated with four consecutive days of trial before the formal test (Fig. 2a). In the MWM, mice that successfully reached the escape platform within 60 s were counted as having passed the test (Fig. 2b). Aged mice exhibited a lower passing ratio compared with young mice (Fig. S4a). Quantification of representative swim paths (Fig. 2c, d) suggested that the aged mice had a longer latency to find the platform and T-Mit significantly shortened the latency of the aged mice. Given that the aged mice exhibited lower swimming speeds (Fig. S4b), to avoid physical bias in age, we assessed memory and learning by comparing improvements in latency during the training process, using path length to normalize each mouse’s trial performance (Fig. 2d, right panel). The results showed that aged mice lost memory and learning compared to young mice and T-Mit significantly reduced the loss of memory and learning in aged mice (Fig. 2d, right panel). In TMSA (Fig. 2e, left panel), aged mice showed a significantly lower percentage of correct alternation, suggesting spatial memory and cognitive decline. After a 2-month treatment with T-Mit, the deficit or loss of spatial learning in aged mice was alleviated (Fig. 2e, right panel). Specific regions of the brain, such as hippocampus, basal forebrain and prefrontal cortex, contribute to this task [63]. NOR as a non-stressful task, assesses cognitive behavior in small rodents (Fig. 2f, left panel) [64]. In the NOR, aged mice preferred to visit a familiar object rather than a new one and T-Mit rescued the cognitive decline (Fig. 2f, right panel). Histological analysis of the hippocampus suggested that T-Mit significantly reduced neuronal loss and neuronal degeneration in the hippocampus of aged mice (Fig. 2g). FACS analysis indicated that T-Mit decreased the expression of p16INK4A (a senescence marker [65]) (Fig. 2h, i) in NeuN^+^ neurons and ROS production within the CSF of aged mice [66] (Fig. 2j). Nissl staining using cresyl violet [67] suggested that the Nissl bodies in neurons decreased in aged mice, while T-Mit rescued the Nissl body loss in aged mice (Fig. 2k). Collectively, orally administered T-Mit rescues the neuropathological alterations and cognitive dysfunction in aged mice.

### T-Mit is preferentially taken up by microglial cells in a T-Mit PS-dependent manner via the TREM2-mediated pathway

**Fig. 3:**
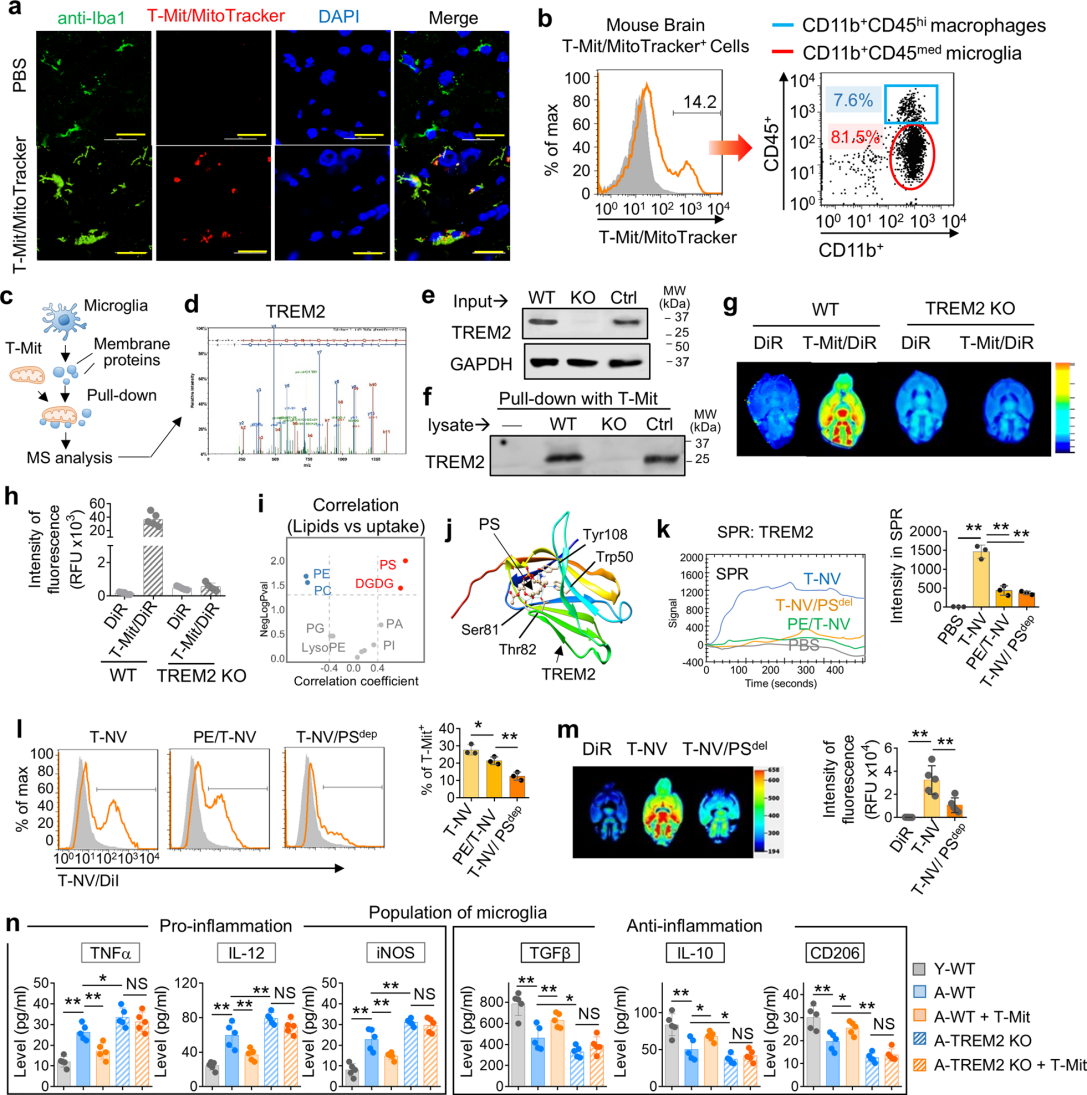
T-Mit is preferentially taken up by microglia via TREM2 and modulates the microglia in aged mice.** a** Immunofluorescence of microglia (Iba1^+^) and T-Mit/MitoTracker in the brain at 2 h post gavage (10 mg/each, *n* = 5). Cell nuclei were counterstained with 4',6-diamidino-2-phenylindole (DAPI). Scale bars, 20 μm. **b** Brain cells isolated from the mice above. The T-Mit/DMitoTracker-positive cells were gated (left panel) and identified as microglia (CD11b^+^CD45^med^ in the red gate) (right panel) using flow cytometry (FACS). **c** Analysis workflow of microglial membrane protein-mediated T-Mit uptake. T-Mit was mixed with the lysates of microglial cytoplasmic membrane. T-Mit-binding protein was isolated by centrifugation and identified using HPLC–MS/MS. **d** The MS/MS spectrum of the tryptic peptide from T-Mit interacted with the protein identified as TREM2. **e** Western blot analysis of TREM2 in microglial lysates from the wild-type (WT) and TREM2 knockout (KO) mice. *Trem2* sgRNA CRISPR Lentivirus was intracranially injected (> 1 × 10^7^ IU/ml) and control CRISPR/Cas9 was used as non-specific negative control (Ctrl). **f** T-Mit incubated with microglial membrane proteins isolated from WT and TREM2 KO mice, followed by pull-down of T-Mit-interacting complexes. TREM2 expression was analyzed by Western blot. **g** Representative fluorescent images of the brain from aged WT (*n* = 5) and TREM2 KO mice fed with 10 mg DiR dye-labeled T-Mit for 2 h.** h** Quantification of image fluorescence intensity in **g**. **i** Volcano plots of Spearman correlation coefficients (x-axis) and significance with *P*-value (y-axis) showing a correlation between the lipids in P-Mit and the uptake efficiency of microglia. Significant positive and negative associations (*P* < 0.05) highlighted in red and blue, respectively. **j** Potential 3D structures of the interaction between protein (TREM2) and lipid (PS) of T-Mit, predicted using UCSF Chimera. Four amino acids of the TREM2 protein are involved in the binding at the positions indicated by the hydrogen bonds. **k** Surface plasmon resonance (SPR) analysis of the interaction (left panel) and quantification of signals in SPR (right panel). TREM2-biotin protein was immobilized on a streptavidin sensor chip, and pretreated with/without PE. T-Mit lipid-derived nanovesicle (T-NV) with/without PS depletion (PS^del^) via the flow (left panel). **l** FACS analysis of BV2 cells treated with DiI-labeled T-NV, PE/T-Mit, T-NV/PS^del^ (left) and quantification of T-NV/DiI^+^ population in FACS (right). Isotype-matched control antibody shown in gray color. **m** Representative fluorescent images of brains from aged mice (*n* = 5) fed with 10 mg DiR dye-labeled T-NV or T-NV/PS^del^ for 2 h (left panel). Quantification of image fluorescence intensity (right panel). **n** ELISA analysis of levels of pro-inflammatory markers tumor necrosis factor alpha (TNFα), interleukin (IL)-12, inducible nitric oxide synthase (iNOS), and anti-inflammatory markers transforming growth factor beta (TGFβ), IL-10 and CD206 in young and aged WT mice as well as aged *Trem2* KO mice receiving T-Mit gavage over a 2-month period. Data from three independent experiments are represented as mean ± SD. * *P* < 0.05, ** *P* < 0.01, NS, not significant, chi-square test for categorical variables; one-way ANOVA followed by post-hoc *t*-tests test for continuous variables

Next, we determined the cellular mechanisms underlying the T-Mit-mediated rescue of the cognitive function in aged mice. We first determined the specific cell type targeted by T-Mit in the brain. T-Mit was labeled with mitochondrial specific fluorescent dye MitoTracker, and was given to the aged mice via gavage. Immunofluorescence performed at two hours post gavage demonstrated the colocalization of T-Mit/MitoTracker with Iba1, a marker of activated microglia in the brain (Fig. 3a). We also tested T-Mit distribution. The T-Mit labeled with lipophilic dye DiI was fed to aged mice and the colocalization of T-Mit/Dil and Iba1 cells was also observed (Fig. S4c). To further identify the cell targets of T-Mit, mouse brain cells were isolated [68] for FACS analysis (Fig. S5a). Results showed that the majority of T-Mit/MitoTracker-positive cells (Fig. 3b, left panel) were CD11b^+^CD45^med^ microglial cells [69] (81.5% ± 4.1%, mean ± SD) (Fig. 3b, right panel and Fig. S5b) and a minor percent was GFAP^+^CD11b^−^ astrocytes [70] (6.7% ± 1.5%) (Fig. S5c). Neurons (NeuN^+^) [71] or oligodendrocytes (MOG^+^CD11b^−^) [72] did not take up T-Mit (Fig. S5c). To further determine whether microglia are the predominate cells that take up T-Mit, we depleted microglia from the brain by intracranial injection of Clodronate [73]. The T-Mit/MitoTracker signals were declined in the brain after Iba1^+^ microglia depletion by Clodronate treatment (Fig. S5d), suggesting that microglia are the primary recipient of T-Mit in the brain. The uptake efficiency of P-Mit by microglia was further assessed in BV2 cells using FACS (Fig. S5e). Phagocytosis and micropinocytosis are major forms of endocytosis that mediate the internalization of particles by macrophages [74]. The BV2 cell uptake of P-Mit including T-Mit was dramatically inhibited by the phagocytosis inhibitor cytochalasin D, but not by the micropinocytosis inhibitor amiloride (Fig. S5f). Membrane proteins of cells play crucial roles in mediating internalization of particles [74]. To identify the molecular mechanism of microglial uptake of T-Mit, CD11b^+^CD45^med^ microglial cells were sorted with FACS and plasma membrane proteins were extracted according to the previously described methods [75, 76]. The membrane proteins were then incubated with T-Mit. The T-Mit complex was collected by centrifugation and the T-Mit-interacting proteins were identified by LC–MS/MS (Fig. 3c). TREM2 was subsequently identified as a potential T-Mit-binding protein by MS (Fig. 3d). To verify the MS result, TREM2 was knocked out (KO) by intracranial injection of TREM2 sgRNA CRISPR Lentivirus in mouse brain (> 1 × 10^7^IU/mL). Western blot confirmed TREM2 KO in brain microglial cells two months after injection [77] (Fig. 3e). The T-Mit interacted with TREM2 in wild-type mice but not TREM2 KO mice (Fig. 3f). TREM2 KO also abolished T-Mit/DiR in the brain (Fig. 3g, h). Western blot confirmed that TREM2 is primary expressed in microglia in the brain (Fig. S5g), consistent with previous studies [78, 79]. Collectively, these data indicate that T-Mit is preferentially taken up by microglial cells via the TREM2-mediated pathway.

Mitochondrial membranes have a distinct lipid composition and phospholipids play critical roles in the phagocytosis processes [80]. Since phagocytosis plays a role in the uptake of all 4 P-Mits we tested (Fig. S5f), we hypothesize that the P-Mit membrane lipid interacts with microglial membrane protein TREM2 to mediate the phagocytic process. To test our hypothesis, P-Mit lipids were extracted using chloroform and the lipid composition was identified by lipidomic analysis using triple quadrupole MS [39] (Fig. S6a and Table S1). Principal component analysis (PCA) revealed clear separation among the four P-Mit groups, with principal component 1 (PC1) accounting for 45.7% of the variance, while PC2 accounting for an additional 31.8%, together explaining over 77% of the total variability in the data (Fig. S6b). The PCA analysis revealed that the overall lipid composition was more similar among T-Mit, Gin-Mit and Gar-Mit since their PCA plots closely resemble each other (Fig. S6b). In contrast, the composition of A-Mit lipids showed more variation.

To further determine which lipids serve as “eat me”/ “do not eat me” signals that mediate phagocytosis of P-Mit by microglia, a correlation analysis between P-Mit lipid composition (Fig. S6a) and the uptake efficiency (Fig. S5e) was conducted using Spearman’s correlation coefficient test (Fig. S6c). The correlation analysis suggested that PS and digalactosyldiacylglycerol (DGDG) served as “eat me” signals enhancing P-Mit uptake by microglia (Fig. 3i), while phosphatidylcholine and phosphatidylethanolamine (PE) served as “do not eat me” signals to prevent microglial uptake of T-Mit (Fig. 3i). To determine the molecular binding mode and structural interactions between PS and TREM2, docking simulations were performed using the extracellular domain of TREM2 (amino acids 19–171) and AutoDock Vina [81]. Interaction analysis using UCSF Chimera [82] suggested that PS forms seven hydrogen bonds with four amino acid residues located at Trp50, Ser81, Thr82, and Tyr108. A Vina score of − 5.2 indicated a strong binding affinity between PS and the TREM2 extracellular domain. The three-dimensional molecular model and potential interactions between PS and TREM2 were further analyzed using AlphaFold 3 and UCSF Chimera (Fig. 3j).

To experimentally verify the correlation between mitochondrial lipids and uptake by microglia, the lipids were isolated from T-Mit and were used to generate nanovesicles (T-NV) using NanoGenerator™. To assess the role of T-Mit PS in the interaction with microglia, PS was depleted from the total lipids of T-Mit [39] using thin-layer chromatography (Fig. S6d). The remainder of the lipids were reassembled into nanovesicles (T-NV/PS^del^) using NanoGenerator™. SPR analysis confirmed the interaction of TREM2 and T-NV. We found that the interaction between TREM2 protein immobilized on the sensor chip and T-NV as the flow, was diminished by pretreatment with free PE or after PS depletion from T-NV (Fig. 3k). FACS analysis of BV2 cells further demonstrated that the uptake of T-NV by microglia was significantly reduced in PS (one of the “eat me” signals) depletion condition and in the presence of PE (one of the “do not eat” signals) (Fig. 3l). In vivo image analysis indicated that PS depletion attenuated T-NV trafficking to the aged mouse brain (Fig. 3m). These data suggested that T-Mit uptake by microglia is mediated by microglial membrane protein TREM2 and T-Mit PS. These data explained our observation of increased T-Mit uptake by microglia with higher expression of TREM2 (Fig. S6e, f). The TREM2-related excessive microglial phagocytosis and induction of pro-inflammatory microglia are linked to the aging-related neurodegeneration [83, 84]. To evaluate microglial immune responses, pro- and anti-inflammatory microglial phenotypes were characterized by quantifying representative cytokine and marker expression levels using ELISA. Aged microglia exhibited increased levels of pro-inflammatory markers, including tumor necrosis factor-alpha (TNFα), IL-12 and iNOS, together with reduced levels of anti-inflammatory markers, including transforming growth factor beta (TFGβ), IL-10 and CD206, compared with young microglia. T-Mit treatment reversed these aging-associated inflammatory changes in WT microglia but not in TREM2-deficient microglia (Fig. 3n). These data suggested that T-Mit promotes the conversion of microglia from a pro-inflammatory to an anti-inflammatory phenotype in a TREM2-dependent manner. 

### T-Mit fuses with M-Mit via MFN1 to form hybrid mitochondria (TM-Mit)

**Fig. 4:**
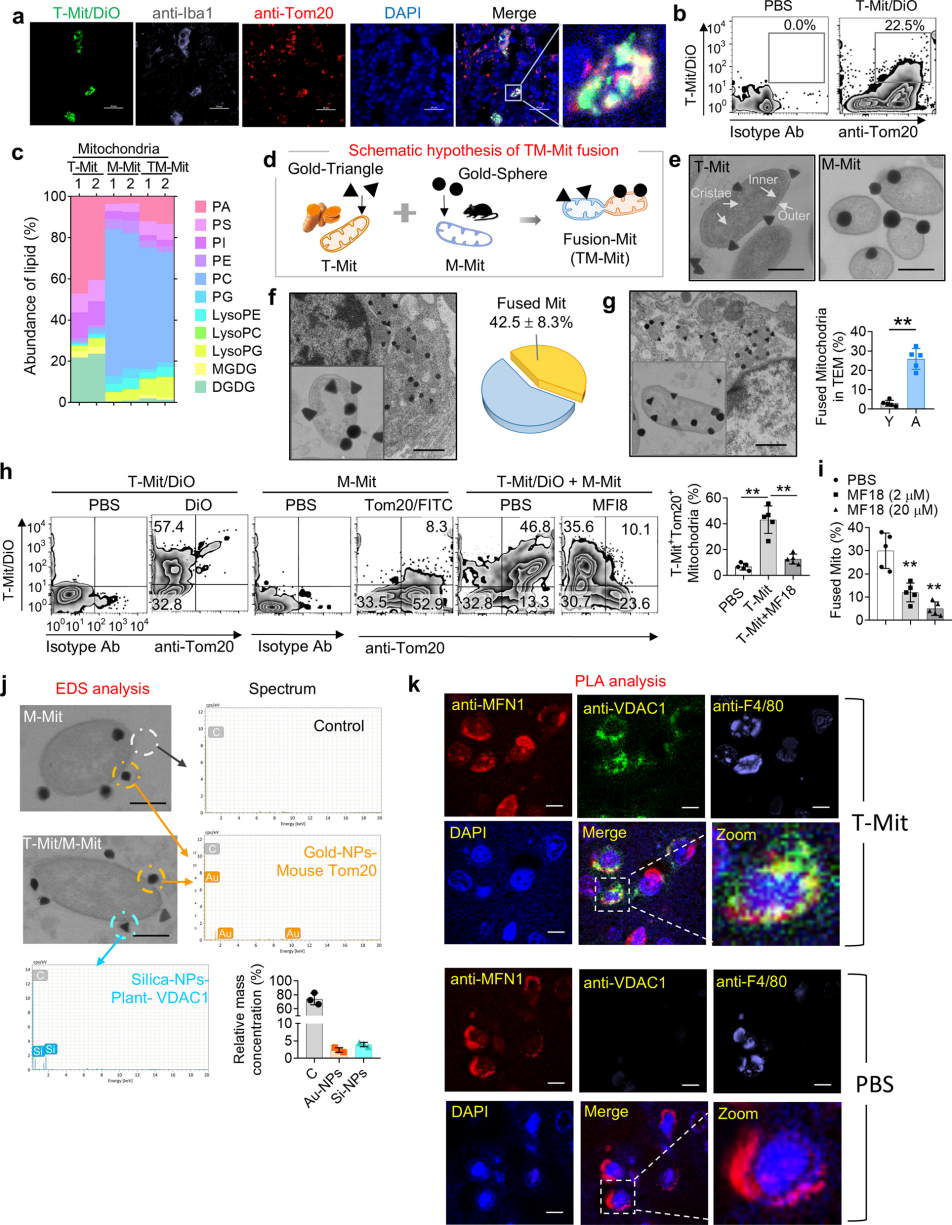
T-Mit fuses with microglial mitochondria (TM-Mit). **a** Immunofluorescence of T-Mit/DiO, microglial marker (Iba1^+^) and mitochondrial marker (Tom20) in brain by confocal microscopy 2 h post gavage (10 mg/each, *n* = 5). Cell nuclei counterstained with DAPI. Scale bars, 20 μm. **b** Mitochondria were isolated from microglia of mice fed with T-Mit/DiO for 6 h and T-Mit/DiO^+^Tom20^+^ mitochondria were identified by FACS. **c** Lipids were extracted from T-Mit, untreated microglial mitochondria (M-Mit), T-Mit/DiO^+^Tom20^+^ mitochondria (TM-Mit) and lipid composition was identified by LC–MS. **d** Strategy of labeling for T-Mit and M-Mit using triangular and spherical gold-nanoparticles (NPs), respectively. **e** Transmission electron microscopy (TEM) of gold‑triangle-labeled T‑Mit (left) and gold‑sphere-labeled M‑Mit (right). Scale bars, 500 nm. Arrows indicate the T‑Mit outer membrane, inner membrane, and cristae. **f** A representative TEM image of gold-triangle^+^ and gold-sphere^+^ mitochondria in BV2 cells (left panel). Scale bar, 500 nm. Gold-Triangle-labeled T-Mit incubated with BV2 cell for 2 h, followed by isolation of BV2 mitochondria. BV2 mitochondria labeled with gold-sphere NPs/protein A using an anti-Tom20 antibody. Fused mitochondria (Triangle^+^/Sphere^+^) visualized by TEM (left bottom). Quantification of Gold-Triangle^+^ and Gold-Sphere^+^ mitochondria (right panel). **g** Gold-Triangle-labeled T-Mit was fed to young (Y) and aged (A) mice. A representative TEM of Gold-Triangle^+^ and Gold-Sphere^+^ mitochondria in aged microglia (left panel). Scale bar, 500 µm. Mitochondria were isolated from microglia and fused mitochondria visualized by TEM (left bottom). Quantification of Gold-Triangle^+^Gold-Sphere^+^ fused mitochondria in microglia (right panel). **h** FACS analysis of Tom20 in T-Mit labelled with DiO and M-Mit in naïve aged mice. Aged mice received oral gavage of T-Mit/DiO (10 mg/each, *n* = 5) and mitochondrial fusion inhibitor MFI8 (10 μM) by intraperitoneal injection. After 6 h, the mitochondria were isolated from the microglia and T-Mit/DiO^+^Tom20^+^ mitochondria identified by FACS (left panel). Quantification of Mit/DiO^+^Tom20^+^ mitochondria (right panel). **i** BV2 cells were pretreated with MFI8 (2 µM and 20 µM). The fused mitochondria (Mit/DiO^+^Tom20^+^) assessed by FACS. **j** BV2 cells were incubated with T-Mit labeled with anti-VDAC1/silica-coated gold nanoparticles, then BV2 mitochondria labeled with anti-Tom20 antibody/gold nanoparticles were isolated. Representative spectrums of Gold (Au) and Silica (Si) in an energy dispersive spectroscopy (EDS) analysis on TEM grids to validate the different elemental distribution on mitochondria. Scale bars, 500 nm. **k** In-situ proximity ligation assay (PLA) analysis of proximity between murine MFN1 (red) and T-Mit VDAC1 (green) in microglia of aged mice fed with T-Mit. Scale bars, 10 μm. Data are representative of three independent experiments (error bars, SD). * *P* < 0.05, ** *P* < 0.01, chi-square test for categorical variables; ANOVA followed by post-hoc* t*-tests for continuous variables

To further locate the microglial intracellular organelle targeted by T-Mit, T-Mit labelled with fluorescent dye DiO was administered to mice via oral gavage. Immunofluorescence indicated that T-Mit signals were colocalized with the microglial marker Iba1 and mitochondrial marker Tom20 in the brain (Fig. 4a), indicating that T-Mit resided with the microglial mitochondria. Subsequently, the CD11b^+^CD45^med^ microglia cells were sorted from the brain cells using FACS and mitochondria were further isolated by gradient centrifugation [85]. FACS analysis suggested that more than one fifth of Tom20^+^ microglial mitochondria exhibited the signal of T-Mit/DiO (Fig. 4b). The T-Mit^+^Tom20^+^ TM-Mit were sorted by FACS. The lipid profiling by MS/MS showed that the lipid compositions of T-Mit and M-Mit were significantly different (Fig. 4c). The abundance of TM-Mit lipids, including phosphatidic acid, PS, PE, LysoPG and DGDG, was quantitatively intermediate between the levels observed in T-Mit and M-Mit (Fig. 4c and Table S2). This finding indicated that TM-Mit is a subset of mitochondria generated after T-Mit fusion with M-Mit.

As mitochondria are dynamic organelles with the ability to fuse and undergo fission in animal and plant cells [51], we hypothesized that the plant-derived T-Mit might physically fuse with the mammalian mitochondria in the T-Mit recipient cells (Fig. 4d). To test this hypothesis, we labeled T-Mit and M-Mit with gold-nanoparticles in triangular and spherical shapes, respectively. Specifically, the T-Mit were labeled with gold-triangle nanoparticles and mouse M-Mit are labeled with Gold-Sphere nanoparticles/protein A beads followed by anti-Tom20 antibody binding, respectively. TEM confirmed labeling in vitro (Fig. 4e). Next, the gold-triangle nanoparticle-conjugated T-Mit were added to BV2 cells and intracranially injected in aged mice. The mitochondria in BV2 cells and mouse brain cells were labeled with gold-sphere nanoparticles and Tom20 antibodies. Interestingly, gold nanoparticles of both shapes were seen in the same mitochondria of BV2 cells (42.5% ± 8.3%) (Fig. 4f) and brain cells (Fig. 4g), suggesting fusion of T-Mit with mitochondria of mouse recipient cells after internalization. Similarly, Gin-Mit, Gar-Mit and A-Mit also fused with BV2 mitochondria (Fig. S7a) and the human macrophage-like cell line, U937 cells (Fig. S7b), using TEM (Figs. S7a and S7b) and FACS (Fig. S7c), respectively. T-Mit and A-Mit displayed the highest and lowest fusion percent, respectively (Fig. S7c). FACS further confirmed and quantified the fusion (Fig. 4h). The mitochondrial fusion was diminished by the mitochondrial fusion inhibitor MFI8 [86] in vivo (Fig. 4h) and in vitro (Fig. 4i).

In addition to TEM imaging and FACS analysis, we performed EDS analysis on TEM grids to identify the distinct element in gold nanoparticle-M-Mit and silica-coated gold nanoparticle-T-Mit, ensuring accurate identification of fused structures on a single mitochondria (Fig. 4j). Proximity Ligation Assay (PLA) [87] demonstrated sub-40 nm proximity between murine mitochondrial membrane protein MFN1 and the P-Mit outer membrane protein VDAC1 [31], with PLA puncta indicating distinct colocalization of MFN1 and VDAC1 within BV2 mitochondria (Fig. 4k).

**Fig. 5:**
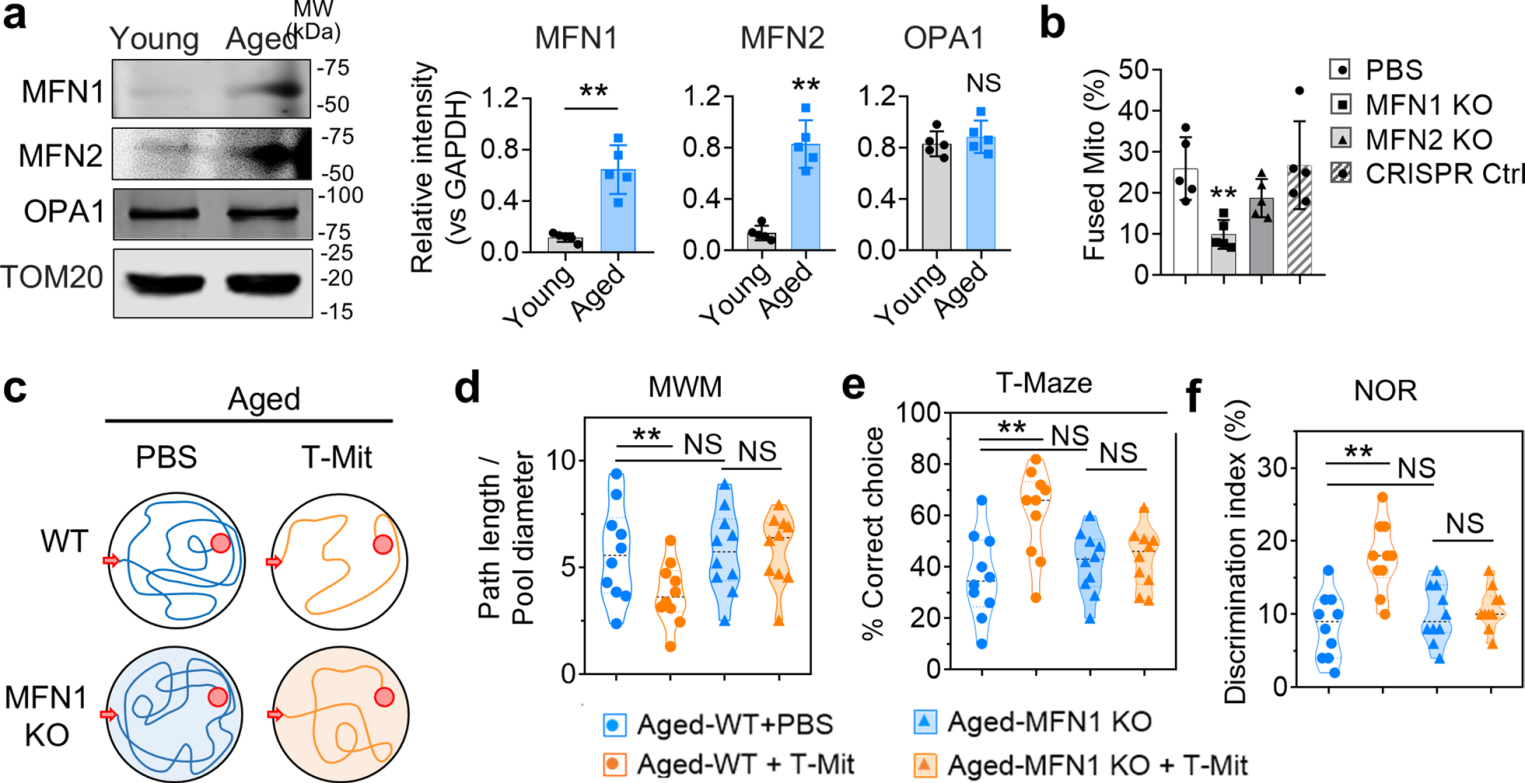
MFN1-dependent fusion between T-Mit and M-Mit contributes to the rescue of aging-related cognitive dysfunction.** a** Western blot analysis of MFN1, MFN2 and OPA1 levels in M-Mit of young and aged mice, and quantification. Tom20 used as the loading control. **b** Analysis of TM-Mit fused mitochondria in WT, *Mfn1* KO and *Mfn2* KO mice by FACS. **c** Representative search paths of mice on the day of test in the MWM assay (*n* = 10 each group). **d**–**f** Quantification of MWM performance on day 5 (**d**), T-maze alternation (**e**), and the discrimination index in the NOR test (**f**). Data are representative of three independent experiments (error bars, SD). *P < 0.05, **P < 0.01, NS, not significant; chi-square test for categorical variables; ANOVA along with post-hoc *t*-tests for continuous variables

Accumulating evidence suggests that three GTPases, including MFN1, MFN2 and OPA1, are involved in mitochondrial fusion and cristae remodeling on the outer and inner membrane of mitochondria [88]. Thus, we next assessed the expression of these genes in microglia. Western blots revealed increased expression of MFN1 and MFN2 in the M-Mit isolated from aged mice (Fig. 5a). FACS analysis indicated that the T-Mit fusion with M-Mit was abrogated in MFN1-deficient microglial cells (Fig. 5b). The effects of T-Mit on the performance in behavioral assays were abolished in MFN1-deficient aged mice (Fig. 5c–f). Collectively, these findings suggest that T-Mit taken up by microglia subsequently target and fuse with the mitochondria of the recipient cells. The microglial outer membrane protein MFN1 mediates the fusion of T-Mit with microglial M-Mit and the formation of hybrid TM-Mit.

### Turmeric-microglial mitochondrial fusion reprograms mitochondrial energic metabolism in microglia of aged mice

**Fig. 6:**
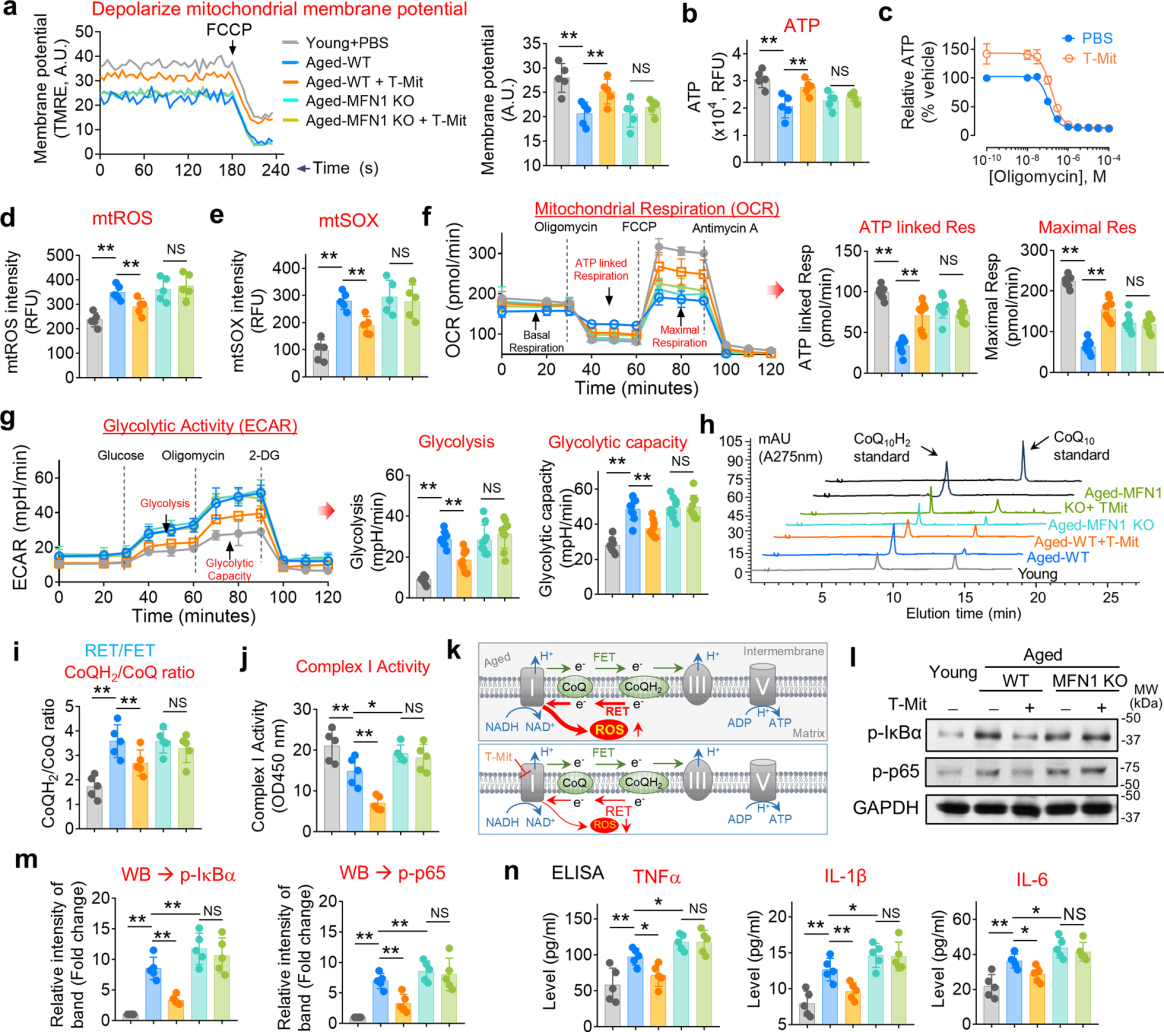
Turmeric-microglial mitochondrial (TM-Mit) fusion reprograms the aging-related dysfunction of mitochondrial complex I in microglial cells. **a** TMRE analysis of mitochondrial membrane potential of microglia from young, aged WT and aged *Mfn1* KO mice (*n* = 5 per group) fed with T-Mit for 2 months. **b** ATP activity measured in freshly aliquoted cells (2 × 10^5^ cells). **c** ATP monitoring of microglia incubated with T-Mit at 37 °C for 2 h and subsequently exposed to ATP synthase inhibitor oligomycin with different concentrations indicated in the plot. **d** Mitochondrial reactive oxygen species (mtROS) in microglia measured with MitoBright ROS Deep Red. **e** Mitochondrial superoxide (mtSOX) in microglia measured with SOX indicator MitoSOX™. **f** Oxygen consumption rate (OCR) analysis (left), and quantification of ATP linked respiration (middle) and maximal respiration (right). **g** Extracellular acidification rate (ECAR) analysis (left), and quantification of glycolysis in glucose conditions (middle) and glycolytic capacity (right). Both OCR and ECAR were assessed using an Agilent Seahorse Analyzer and values were normalized to the total cell number. **h** Representative HPLC traces for the oxidized and reduced form of coenzyme Q (CoQ and CoQH_2_) from microglia.** i** Quantification of the CoQH_2_/CoQ ratio. **j** Mitochondrial complex I activity assessment. **k** Schematic model showing the induction of reverse electron transfer (RET) and ROS generation at complex I in the mitochondrial inner membrane of aged microglia. T-Mit rescued ATP generation by inhibiting RET via complex I and reprograming mitochondrial energy metabolism. **l, m** Western blot analysis of NF-κB key components phosphorylated IκBα (p-IκBα) and phosphorylated p-65 (p-p65) (**l**), and quantification of band intensity (**m**).** n** ELISA analysis of TNFα, IL-1β and IL-6 in microglia cell lysates. Data are representative of three independent experiments (error bars, SD). *P < 0.05, **P < 0.01, NS, not significant; chi-square test for categorical variables; ANOVA followed by post-hoc *t*-tests for continuous variables

Mitochondrial membrane potential directly powers many critical functions of mitochondria, including ATP and ROS production, mitochondrial protein import, and metabolite transport [89]. Alteration of mitochondrial membrane potential is a feature of aging and mitochondrial diseases [90]. Given that T-Mit fuses with the M-Mit, we next sought to determine whether T-Mit could affect mitochondrial membrane potential. We assessed mitochondrial membrane potential in microglia using the TMRE assay [91]. The results showed that microglia from aged mice had a relatively lower potential compared to those from young mice, whereas T-Mit partially restored the mitochondrial membrane potential in the aged microglia (Fig. 6a). This effect was abolished MFN1-deficient mice (Fig. 6a), exhibiting less mitochondrial binding and inhibition of electron transport chain. Mitochondrial membrane potential is a key indicator of mitochondrial activity in response to ATP synthesis and ROS production [92–94]. Therefore, we next examined the impact of T-Mit on the ATP synthase activity in microglia. The results showed that the ATP activity in mitochondria was lower in aged microglia and that T-Mit improved ATP production in aged mouse microglia in a MFN1-dependent manner (Fig. 6b). In mammalian cells, ATP is primarily produced from oxidation in mitochondria and very little ATP is generated via aerobic glycolysis [92–94]. We found that the ATP production in T-Mit-treated microglia was diminished when the cells were exposed to mitochondrial ATP synthase inhibitor oligomycin (Fig. 6c), suggesting that T-Mit enhances microglial ATP synthase activity via mitochondrial redox metabolism.

Mitochondria generate ATP through OXPHOS by electron transport [95]. OXPHOS in mitochondria also generates ROS including the superoxide (SOX) anion (O_2_^•−^), hydrogen peroxide (H_2_O_2_) and hydroxyl radical (OH), which can inhibit the activity of mitochondrial ATP synthase and NADH dehydrogenase [96]. Next, we assessed mtROS in microglia using MitoBright ROS Deep Red. Moreover, SOX indicator MitoSOX™ (Invitrogen) was used to estimate mtSOX, as SOX is the most abundant ROS in mitochondria. The results suggested that the levels of mtROS (Fig. 6d) and mtSOX (Fig. 6e) were significantly higher in the microglia of aged mice compared to young mice. T-Mit reduced the levels of mtROS (Fig. 6d) and mtSOX (Fig. 6e) in microglia from aged WT mice, and this effect was abolished in aged MFN1-KO mice. Next, we evaluated mitochondrial respiration and glycolysis by measuring oxygen consumption rate and extracellular acidification rate. We found that T-Mit reversed the reduction of oxygen consumption rate at the level of ATP-linked respiration and maximal respiration (Fig. 6f), while restored the induction of extracellular acidification rate at glycolysis and glycolytic capacity (Fig. 6g) in microglia from aged mice.

The levels of ATP and ROS generation rely on the mitochondrial electron transport chain. Electrons flow in the opposite direction from reduced ubiquinone (CoQH_2_) to oxidize ubiquinone (CoQ) and complex I is considered to be the major source of ROS generation [97, 98]. This process known as RET is activated during aging and aging-related neurodegeneration [17, 22]. To determine the influence of T-Mit on the electron transport in microglia, we measured the reduced/oxidized form of coenzyme Q (CoQH_2_/CoQ) in microglia by HPLC (Fig. 6h). The results suggested that T-Mit significantly reduced RET activity indicated by the CoQH_2_/CoQ ratio (Fig. 6i) and complex I activity (Fig. 6j) in the mitochondria of aged mice. The attenuation of RET by T-Mit is consistent with the reduction of ROS with T-Mit treatment (Fig. 6d, e, k). The attenuation is RET-specific as there is no evidence of effect of T-Mit on the conversion of NADH and NAD^+^ (Fig. S7d). The effects of T-Mit were abolished in MFN1-deficient mice (Fig. 6a-j), suggesting that the MFN1-mediated fusion is required for T-Mit inhibition of RET and attenuation of complex 1 activity. Although complex I is the major source of ROS production, especially through RET, complex II and complex III also contribute to ROS production. To determine whether they also contribute to the T-Mit-mediated modulation of ROS production, microglia from aged mice were exposed to rotenone, thenoyltrifluoroacetone (TTFA) or antimycin A, which can induce ROS production by targeting complex I, II or III, respectively [99–101]. At 1 h after treatment, microglia were treated with T-Mit (1 mg) for 2 h. We found that the complex I inhibitor rotenone interrupted the T-Mit-mediated downregulation of ROS production (Fig. S7e), while the other two inhibitors did not, indicating that T-Mit modulates M-Mit activity through complex I.

ROS mediates activation of NF-κB via phosphorylation of RelA (p-p65) [102]. Western blots showed phosphorylation of p65 and IκBα in microglia of aged mice and that T-Mit inhibited the NF-κB subunit induction in an MFN1-dependent manner (Fig. 6l, m), while the total levels of the subunits did no change (Fig. S7f, g). Excessive ROS production is tightly linked to the NF-kB pathway-mediated inflammation and cytokine release, which is responsible for the aging-related neurodegeneration process [103]. Activation of NF-κB could contribute to the induction of pro-inflammatory microglia (IL-6^+^TNFα^+^) which play a pathogenic role in the aging process [84]. ELISA results showed that T-Mit decreased the levels of TNFα, IL-1β and IL-6 in microglial cells (Fig. 6n), consistent with the attenuated NF-κB activation. Collectively, T-Mit reprograms mitochondrial energy metabolism at least by repressing mitochondrial complex I activity, thus attenuating the RET process, leading to reduced mtSOX generation, subsequently boosting mitochondrial ATP production.

### T-Mit alleviates aging-related oxidative stress in microglia through Tae-miR319 and Osa-miR166a-3p, which inhibit mitochondrial ND4 and ND5 expression, respectively

**Fig. 7:**
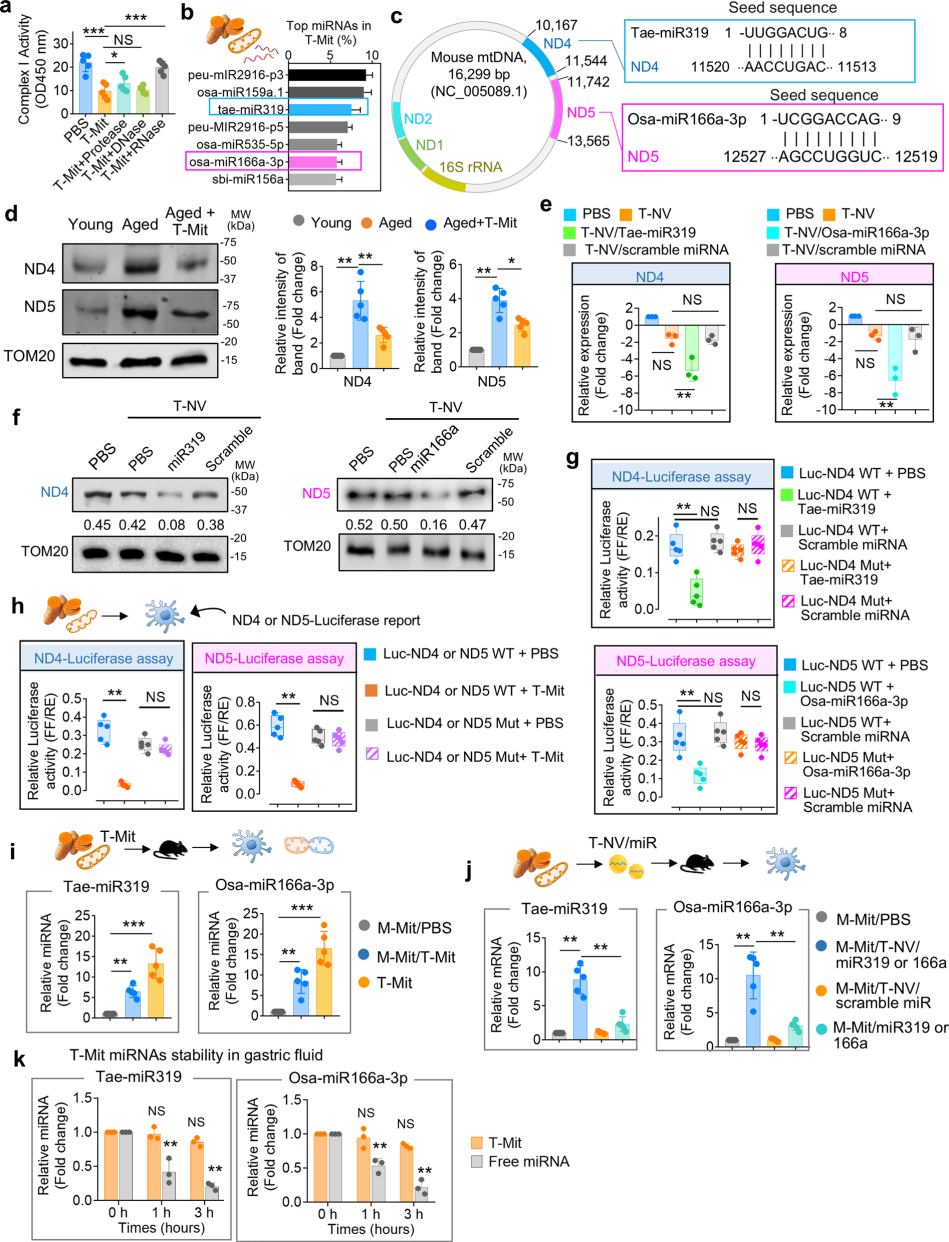
T‑Mit-enriched Tae‑miR‑319 and Osa‑miR166a‑3p mediate the inhibition of mitochondrial ND4 and ND5 expression in microglia. **a** Microglial complex 1 activity analysis using the Complex 1 Enzyme Activity Microplate Assay. Aged mice (*n* = 5) received 1-month oral gavage of T-Mit (10 mg) incubated with protease (1.0 mg/ml), DNase (1.0 mg/ml) or RNase (1.0 mg/ml) at 37 °C for 1 h. **b** List of microRNAs (miRNAs) with top abundance in T-Mit. Total RNA was isolated from T-Mit and small RNAs identified by next-generation NextSeq small RNA sequencing. **c** Simplified diagram of mouse mitochondrial DNA (NC_005089.1) (left panel), showing putative binding sites for T-Mit Tae-miR319 and Osa-miR166a-3p in ND4 and ND5 via 8-nt and 9-nt of seed sequences, respectively. **d** Western blots of ND4 and ND5 levels in microglia from young, aged mice and aged mice fed T-Mit every other day for 1 month, and quantification. **e** qPCR analysis of ND4 (left panel) and ND5 (right panel) in BV2 at 72 h post-transfection of T-Mit-derived nanovesicle (T-NV) encapsulated with 1 μg Tae-miR319 or Osa-miR166a-3p. **f** Analysis of proteins ND4 (left panel) and ND5 (right panel) in BV2 by western blot. **g** Luciferase assay at 72 h after transfection with pEZX-Luc-ND4 & pEZX-Luc-ND5 and Tae-miR319 & Osa-miR166a-3p, respectively in BV2 cells. **h** Luciferase assay for the transfection with pEZX-Luc-ND4 & pEZX-Luc-ND5 followed by T-Mit treatment for 1 day. **i** qPCR analysis of Tae-miR319 and Osa-miR166a-3p in M-Mit of the mice fed with T-Mit for 1 month. **j** Analysis of Tae-miR319 or Osa-miR166a-3p in M-Mit using qPCR. T-NV encapsulated with Tae-miR319 or Osa-miR166a-3p (10 μg/each, every other day) was administered to aged mice (*n* = 5 each group) via oral gavage for 1 week. **k** qPCR analysis of Tae-miR319 and Osa-miR166a-3p in T-Mit. T-Mit (10 mg) or free Tae-miR319 or Osa-miR166a-3 (1 nmol) suspended in 0.1 mL gastric juice collected from aged mice for 1–3 h at 37 °C. Data are representative of three independent experiments (error bars, SD). *P < 0.05, **P < 0.01, ***P < 0.001, NS, not significant; chi-square test for categorical variables; ANOVA followed by post-hoc* t*-tests for continuous variables

Next, we hypothesized that T-Mit modulates complex I activity via targeting specific subunits of complex I. Plant mitochondria carrying proteins and RNAs exert diverse crucial biological activities. To determine which factor in T-Mit contributes to the regulation of mitochondrial complex I activity of microglia, total proteins and RNA were removed from T-Mit using protease and RNase, respectively. ExoGlow™-Protein and ExoGlow™-RNA/DNA reagents were used to specifically label intra-vesicle protein and RNA/DNA, respectively (Fig. S8a). Protease, RNase and DNase are capable of permeating the T-Mit and depleting the protein, RNA and DNA of mitochondria. The mitochondrial membrane potential-based dye MitoTracker was used to monitor whether the T-Mit membrane was affected by the protease, DNase and RNase (Fig. S8b). Collectively, the T-Mit membrane was intact. The complex I activity assay showed that RNase treatment significantly decreased the influence of T-Mit on the complex I activity of microglial mitochondria (*P* = 0.0003) (Fig. 7a). Protease (*P* = 0.037) but not DNase (*P* = 0.41) treatment slightly alleviated the T-Mit-mediated inhibition of the complex I activity (Fig. 7a).

To test whether T-Mit complex I interacts with M-Mit complex I, T-Mit complex I was isolated [28] and labeled with biotin. The biotinylated T-Mit complex I was incubated with the proteins extracted from M-Mit complex I and microglial cytoplasm. The biotinylated T-Mit complex I was then pulled down by streptavidin beads and separated by electrophoresis. Coomassie staining of SDS-PAGE gel showed no additional visible band in T-Mit complex I after incubation with M-Mit complex I or microglial cytoplasm proteins (Fig. S8c). The affinity precipitation-derived proteins were further identified by LC–MS/MS (Table S3). Consistent with the Coomassie staining assay, the result of LC–MS/MS did not suggest direct interaction between T-Mit complex I and M-Mit complex I or microglial cytoplasm proteins. Collectively, these data suggested that the T-Mit RNA cargo plays a key role in the T-Mit modulation of mitochondrial activity.

Given the crucial roles of miRNA in posttranscriptional regulation, we next explored whether T-Mit miRNAs could modulate animal mitochondrial mRNA expression. Total RNA was isolated from T-Mit and small RNA sequencing was conducted by next-generation small RNA sequencing. miRNAs were identified from the sequences of T-Mit small RNAs based on the plant miRNA database and relevant data in publications (Fig. 7b and Table S4). The sequencing data have been deposited in the NCBI Gene Expression Omnibus (GEO) database with accession number GSE165349. To test whether T-Mit miRNAs potentially target microglial mitochondrial mRNAs, the miRNA sequences identified in T-Mit were used to match the sequences of a mouse mitochondrial RNA database using Nucleotide BLAST (https://blast.ncbi.nlm.nih.gov/). Interestingly, two highly rich miRNAs Tae-miR319 and Osa-miR166a-3p in T-Mit containing 8-nucleotide (nt) and 9-nt seed sequences [104] at their 5’-end had perfect complementarity and may potentially bind to microglial mitochondrial gene *ND4* and *ND5*, respectively (Fig. 7c). *ND4* and *ND5* genes encode two important subunits in the mitochondrial complex I that contribute to the proton-coupled electron transfer [105]. Western blot analysis suggested that the levels of ND4 and ND5 were increased in the microglia of aged mice, and T-Mit inhibited their levels (Fig. 7d). To experimentally confirm the miRNA modulation of target gene expression, synthetic Tae-miR319 and Osa-miR166a-3p were encapsulated in T-Mit-derived T-NVs and incubated with BV2 cells. qPCR analysis revealed presence of Tae-miR319 and Osa-miR166a-3p in microglial mitochondria. The expression of *ND4* and *ND5* was reduced by Tae-miR319 and Osa-miR166a-3p, respectively, at both transcriptional mRNA level (Fig. 7e) and protein level (Fig. 7f). BV2 cells were transfected with ND4 and ND5 report plasmids (pEZX-Luc-ND4 and pEZX-Luc-ND5) as well as corresponding mutant constructs containing disrupted binding sites. The luciferase assay showed that ND4 and ND5 activities were modulated by Tae-miR319 and Osa-miR166a-3p, respectively, through their predicted binding sites (Fig. 7g). The effects of these miRNA mimics on ND4 and ND5 luciferase activity were reproduced by T-Mit treatment, confirming that Tae-miR319 and Osa-miR166a-3p contribute to the T-Mit–mediated modulation of ND4 and ND5 expression (Fig. 7h).

To assess the in vivo integrity and stability of T-Mit–derived miRNAs within host mitochondria (M-Mit), aged mice were administered with T-Mit or T-NV encapsulating Tae-miR319 or Osa-miR166a-3p via oral gavage twice weekly. M-Mit was subsequently isolated from brain tissue, and the abundance of Tae-miR319 and Osa-miR166a-3p was quantified by qPCR. Both miRNAs were significantly enriched in the M-Mit from mice receiving T-Mit/miRNAs or T-NV/miRNAs compared with controls treated with PBS, T-NV containing scrambled miRNAs, or free Tae-miR319/Osa-miR166a-3p (Fig. 7i, j). To further evaluate the impact of gastric conditions on T-Mit miRNA stability, we employed an approach analogous to that used for determining T-Mit protein integrity in gastric fluid (Fig. 1l, m). T-Mit encapsulating Tae-miR319 or Osa-miR166a-3p was incubated in gastric fluid at 37 °C for 3 h, and subsequent qPCR analysis demonstrated that T-Mit miRNAs remained intact without detectable degradation, whereas the free form of Tae-miR319 or Osa-miR166a-3p was not detected (Fig. 7k). These findings indicate that T-Mit confers protection against gastrointestinal degradation and enables systemic delivery of functional miRNAs to host mitochondria.

**Fig. 8:**
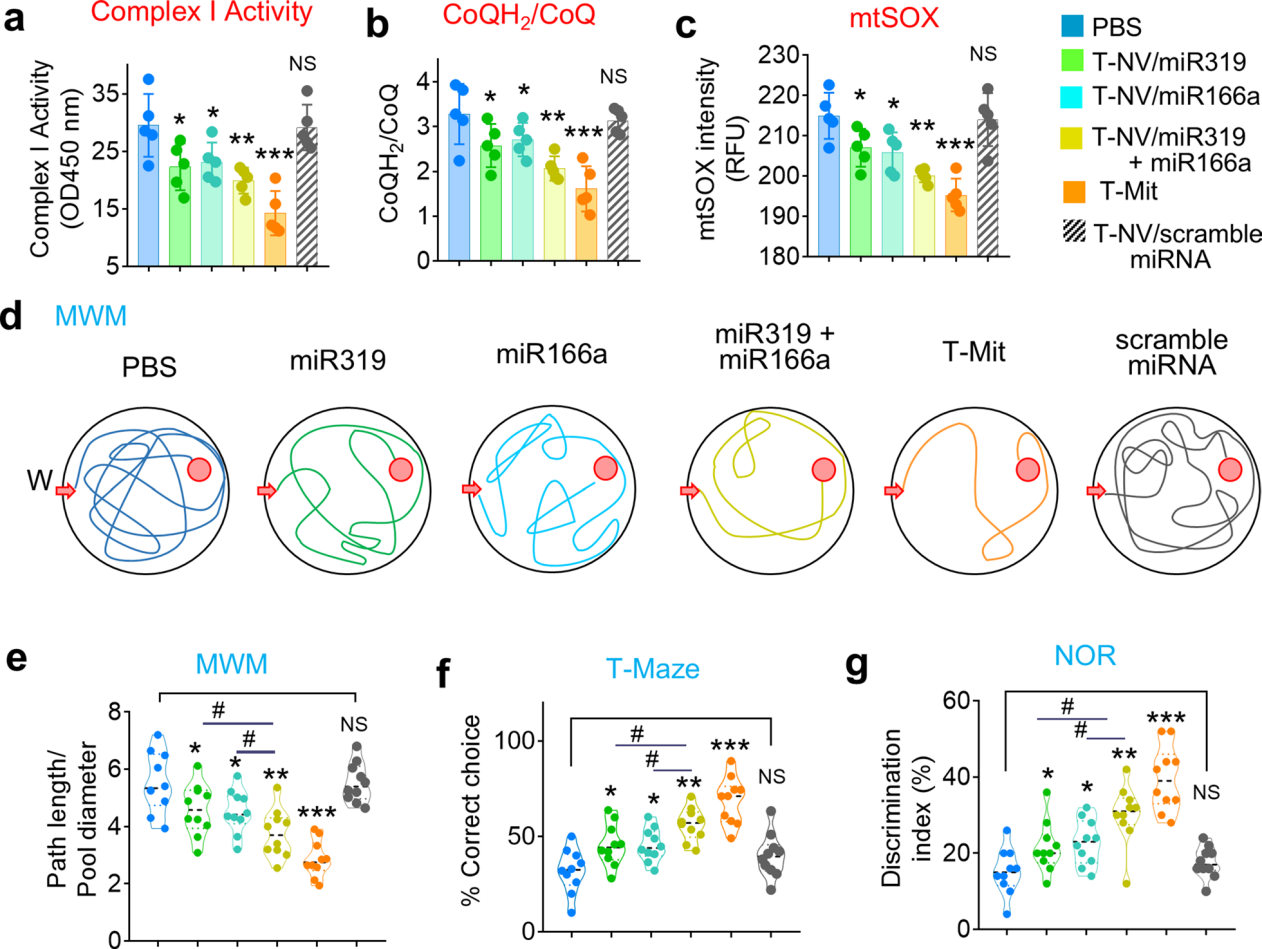
T‑Mit-derived Tae‑miR‑319 and Osa‑miR166a‑3p modulate mitochondrial function and ameliorate aging‑related cognitive decline. **a** Analysis of the mitochondrial complex I activity in microglia. T-NV encapsulated with Tae-miR319 or Osa-miR166a-3p (10 µg/each, every other day) administered to aged mice (*n* = 5 each group) via oral gavage for 2 months. **b** Ratio of CoQH_2_/CoQ in microglia of aged mice measured by HPLC. **c** mtSOX in microglia assessed using MitoSOX™. **d** Representative search paths of mice on the day of the test in the MWM assay (*n* = 10 each group).** e** MWM assay for the mice administrated with T-NV packed with miRNAs. Data are representative of three independent experiments. **f** Quantification of T-maze alternation (*n* = 10 each group). **g** Quantification of discrimination index in NOR test (*n* = 10 each group) with the mean time spent on each object. Data are representative of three independent experiments (error bars, SD). *P < 0.05, **P < 0.01, ***P < 0.001 *vs* PBS, NS, not significant; ^#^*P* < 0.05; chi-square test for categorical variables; ANOVA followed by post-hoc *t*-tests for continuous variables

Given T-Mit and T-NV can traffic into brain and regulate M-Mit complex I subunit gene expression, we next investigated the impact of T-Mit miRNAs on M-Mit complex I-associated activity. Tae-miR319 and Osa-miR166a-3p significantly decreased complex l enzyme activity (Fig. 8a) and the CoQH_2_/CoQ ratio (Fig. 8b), indicating reduction of RET activity through complex I. Consistently, both Tae-miR319 and Osa-miR166a-3p significantly reduced mtSOX production (Fig. 8c) since there was a decline in RET (Fig. 8b). Importantly, Tae-miR319 and Osa-miR166a-3p apparently rescued aging-related cognitive decline (Fig. 8d-g) as efficiently as the T-Mit.

**Fig. 9:**
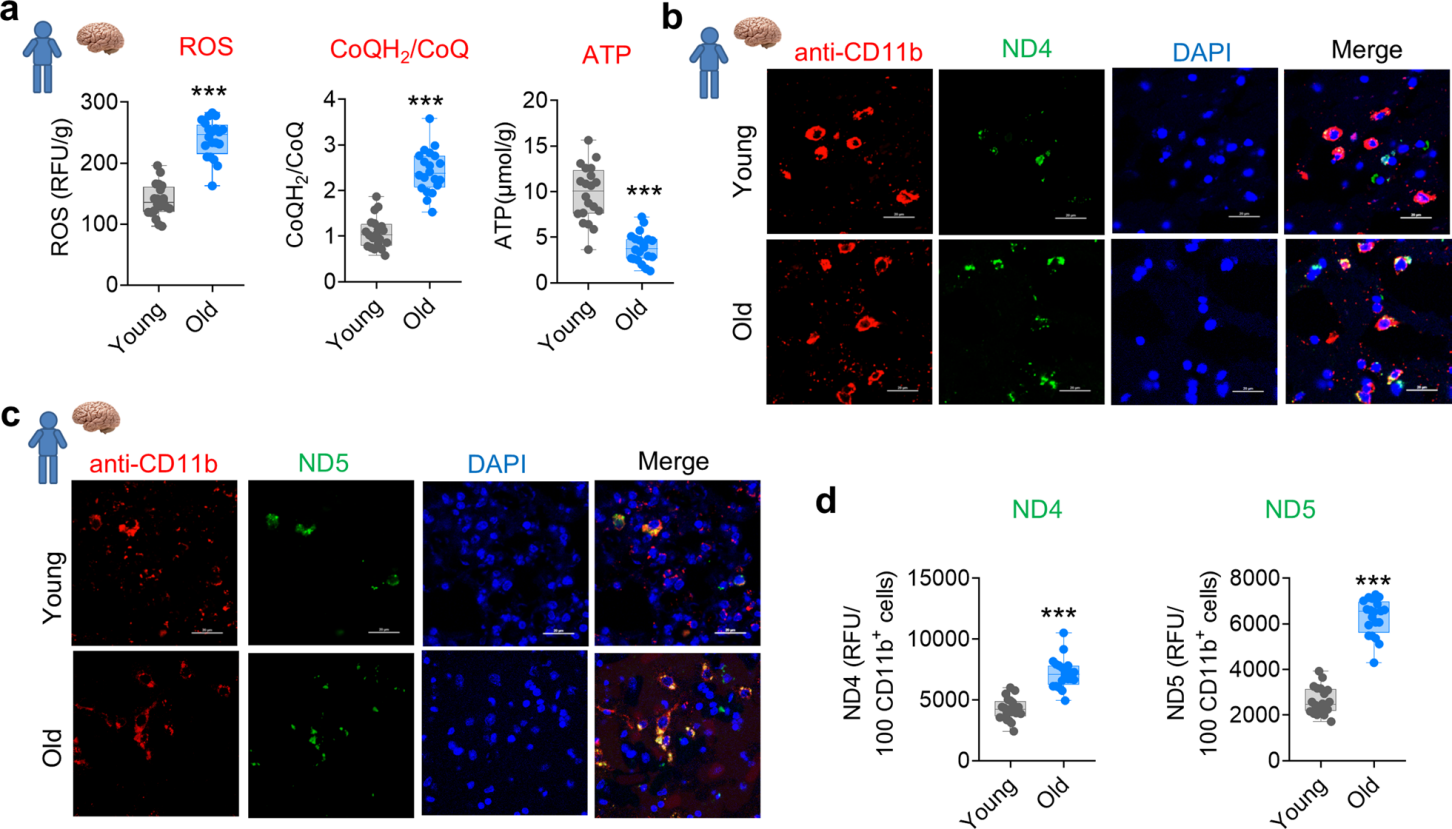
Age‑dependent alterations in NADH dehydrogenase expression and mitochondrial oxidative stress in brain microglia. **a** Comparison of ROS (left), CoQH_2_/CoQ ratio (middle), and ATP level (right) in microglia isolated from the brains of young and old subjects. **b**, **c** Immunofluorescence of ND4 (**b**) and ND5 (**c**) in CD11b^+^ microglia in the brains of young (30.88 ± 4.09 years old, *n* = 20) and old people (70.75 ± 4.59 years old, *n* = 20). Scale bars, 20 µm. **d** Quantification of fluorescent intensity using ImageJ software. Data are representative of three independent experiments (error bars, SD). ***P < 0.001, ANOVA followed by post-hoc* t*-tests for continuous variables

To validate our findings and bridge our animal studies with clinical relevance, microglial cells isolated from human brains of young (30.88 ± 4.09 years old, *n* = 20) and old (70.75 ± 4.59 years old, *n* = 20) donors were analyzed. We found a decline in the ATP level, as well as an augmentation of ROS and the CoQH_2_/CoQ ratio in old donors compared to the young donors (Fig. 9a), indicating RET activation and dysfunction of mitochondrial energy metabolism in aged human brain cells. Immunofluorescence showed that the expression of ND4 and ND5 (Fig. 9b–d) was significantly elevated in microglia from older subjects. These findings are consistent with our mouse data and highlight ND4 and ND5 as translationally relevant biomarkers that may reflect age-associated mitochondrial stress in the human brain. Importantly, their conserved upregulation across species suggests potential utility for monitoring disease progression and therapeutic responses in future clinical studies targeting mitochondrial dysfunction.

Curcumin is a lipid-soluble compound found in turmeric that has been shown to have a positive effect on aging-related neurodegenerative diseases [47]. Therefore, we determined whether curcumin is present in the T-Mit. HPLC analysis indicated there was very little curcumin (1.53 ± 0.60 μg/g) in T-Mit (Fig. S9a, b), whereas the exosome-like nanoparticles [39] isolated from turmeric contained a significant amount of curcumin (139.2 ± 32.7 μg/g) (Fig. S9a, b). The administered dose of turmeric-derived exosome-like nanoparticles (10 mg/mouse/day) contains approximately 1.4 ± 0.33 mg curcumin per mouse/day, which is comparable to previously reported therapeutic curcumin doses (~ 1 mg/mouse/day) used in neuroinflammation and neurodegeneration models [106, 107]. Collectively, these data excluded that curcumin contributes to the effect of T-Mit since the amount of curcumin delivered via T-Mit in our study was ~ 65,000-fold lower than established therapeutic doses [106, 107]. Finally, the potential cytotoxicity of T-Mit was evaluated. The results showed that T-Mit did not affect serum levels of alanine aminotransferase (ALT) and aspartate aminotransferase (AST), two measures of drug-related hepatotoxicity [108], suggesting no significant liver damage in aged mice when used at a therapeutic dose (Fig. S9c).

## Discussion

In this study, we provide evidence supporting our hypothesis that plant–microglial mitochondrial fusion reverses aging-related cognitive dysfunction by reprogramming microglial mitochondrial energy metabolism. Our results demonstrate that orally administered P-Mit can traffic from the gastrointestinal tract to the brain, be preferentially taken up by microglial cells, physically integrate with endogenous mammalian mitochondria, and restore microglial bioenergetic balance in an age-dependent manner.

Using ultrastructural analyses, biochemical profiling, and molecular validation, we show that T-Mit is present within microglial cells following oral administration. Evidence from electron microscopy, lipidomic and proteomic profiling, miRNA encapsulation analysis, elemental mapping of gold nanoparticle–labeled mitochondria, FACS, proximity ligation assays, and MFN1 knockout experiments collectively supports the physical integration of plant mitochondria into the mammalian mitochondrial network. The uptake efficiency was strongly correlated with the level of TREM2, which increases with aging, explaining the preferential accumulation of P-Mit in microglia from aged mice [109, 110]. This finding is significant given the urgent need for non-invasive approaches to targeting microglial mitochondrial dysfunction [5, 111, 112], a key driver of neuroinflammation and neurodegeneration in aging and Alzheimer’s disease [113, 114].

We selected T-Mit as a proof of concept because turmeric is widely recognized for its antioxidant and anti-inflammatory properties. Our data demonstrate that T-Mit has high integrity and superior bioenergetic capacity [115]. Once internalized, T-Mit fuses with endogenous microglial mitochondria in an MFN1-dependent manner, forming functional hybrid mitochondria. This fusion facilitates molecular exchange between plant and host mitochondrial networks. We demonstrate that T-Mit carries plant-derived miRNAs that, following mitochondrial fusion, gain access to the microglial mitochondrial transcriptome and selectively target the coding sequences of mitochondrial complex I subunits ND4 and ND5. This results in partial inhibition of complex I activity [116]. Such regulation is consistent with the architecture of mitochondrial mRNAs, which lack substantial untranslated regions and are therefore regulated primarily through coding sequence interactions [117, 118].

Partial inhibition of complex I reduces mitochondrial ROS production while preserving ATP output, yielding a more efficient and stable bioenergetic state. This aligns with prior studies demonstrating that mild complex I inhibition improves metabolic homeostasis and delays aging-related pathologies [119, 120]. Clinically, metformin and other complex I inhibitors have shown benefits in aging-associated disorders, diabetes, and cancer, underscoring the translational relevance of this mechanism [121, 122]. Hybrid mitochondria further exhibit enhanced complex-I–linked respiration, reduced RET, and decreased oxidative stress. RET is a major source of pathological ROS in aging and neurodegeneration [123]. By reducing RET while maintaining FET, T-Mit restores a favorable ATP-to-ROS ratio in aged microglia, enabling partial functional rejuvenation without reversing aging outright [124].

Mitochondrial dynamics is central to mitochondrial quality control, and our data demonstrate that P-Mit fusion with microglial mitochondria occurs through MFN1. This mitochondrial fusion creates a functional interface that enables the transfer of molecular cargo from T-Mit to host mitochondria. Specifically, the fusion process facilitates the delivery of T-Mit–associated miRNAs into the microglial mitochondrial network, allowing these miRNAs to access and potentially regulate microglial mRNAs encoding key components of the electron transport chain, including complex I subunits ND4 and ND5. As a result, mitochondrial complex I activity is partially inhibited, leading to reduced ROS levels, resulting in enhancement of mitochondrial efficiency and stability, and increased ATP production. This conclusion is also consistent with previous report [13]. We finally demonstrated that orally administering turmeric root mitochondria can rescue cognitive dysfunction in aged mice. The T-Mit downregulated the expression of p16INK4A, inhibited ROS production, and prevented brain cell damage as indicated by Nissl staining. Increased expression of p16INK4A [65, 125] and overproduction of ROS are associated with age-related pathologies [126]. p16INK4A is a key marker and regulator of cellular senescence [65, 125]. Collectively, our findings raise the possibility that mitochondria from distinct edible plant sources may differentially influence fusion and fission pathways, suggesting a potential dietary role in regulating mitochondrial network homeostasis [127, 128]. The balance of fusion and fission of mitochondrial networks is essential for maintaining mitochondrial structure [9]. Mitochondrial fission and fusion are governed by several key proteins that maintain mitochondrial quality control. Fusion is controlled by both outer and inner mitochondrial membrane machinery [9]. Fission is primarily regulated by the GTPase dynamin-related protein 1 (DRP1). Our results presented in this study show that turmeric root mitochondria can fuse with microglial mitochondria in an MFN1-dependent manner. This finding opens a new avenue to further investigate whether P-Mit from other plant species may regulate DRP1 activity. Given the daily intake of diverse edible plants, we speculate that different P-Mit populations may differentially regulate microglial mitochondrial fusion and fission, thereby supporting the importance of dietary diversity for overall health.

P-Mit not only participates in regulating the balance of fusion and fission of mitochondrial networks, but may also regulate the balance between RET and FET. Our data suggest that the RET process is enhanced in microglial cells from aged mice and older people as indicated by increased CoQH_2_/CoQ. T-Mit treatment decreased the CoQH_2_/CoQ ratio and the mitochondrial complex I activity in microglia isolated from aged mice. This may be attributed to the inhibition of expression of complex I subunits ND4 and ND5 by T-Mit miRNAs, resulting in decreased activity of complex I. The reduced activity of complex I leads to decreased ROS production where the mitochondria minimizes electron leakage and oxygen consumption [129]. As a result, a new balance between ATP production and ROS is achieved in the microglia of aged mice. FET is the primary pathway of ATP production, driving cellular energy metabolism. RET can generate ROS, which play roles in cellular signaling and stress response [129]. Our findings suggest that edible P-Mit may serve a physiological role beyond therapy by fine-tuning mitochondrial complex I activity, with distinct P-Mit populations potentially biasing FET or RET to maintain network balance. Although aging cannot be reversed, such modulation may help restore a balanced ATP and ROS state in aged microglia, partially mimicking that of younger cells [130]. 

We further show that mitochondrial lipid composition governs microglial uptake. P-Mit enriched with PS and DGDG is preferentially internalized, whereas PG-enriched mitochondria are less efficiently taken up, consistent with the known ligand specificity of TREM2 [131]. Studies show that TREM2 is predominantly expressed in microglia in the central nervous system and is involved in microglial cell proliferation, survival, migration, and phagocytosis [19, 132, 133]. TREM2 binds to anionic ligands such as phospholipids. Studies of TREM2 clearly demonstrate that activated microglia in AD exacerbate synaptic pruning, causing cognitive impairment [19, 132, 133]. Our data show that T-Mit signals cannot be detected in the brains of young mice via oral administration, which is associated with the level of TREM2 expression. Our study shows that the expression of TREM2 increases with aging in mice. The higher level of TREM2 expression on microglial cells, the more P-Mit taken up by microglia. Whether this conclusion applies to pathogenic conditions such as AD needs to be further investigated. In addition, although we showed that P-Mit phospholipids bind to TREM2 expressed on the microglial cells, more biochemical and structural studies are required to precisely define the interaction of TREM2 with P-Mit lipids. The composition of mitochondrial lipids isolated from one type of P-Mit is different from the mitochondria from other plants, indicating the need to consume a variety of edible plants for humans. It is conceivable that the efficiency of microglial uptake of P-Mit could be dependent on the type of plants a human consumes. Mitochondrial PG serves as a “do not eat me” signal for microglial cells but this may not be the case for other type of brain cells. Further work is needed to determine what type(s) of brain cell take up PG on P-Mit, and whether the ratios of P-Mit PS/DGDG to PG on the same P-Mit regulate homeostasis of microglial cell uptake.

In this study, we also found regional differences in T-Mit uptake by microglia in mouse brain. Microglia can have varied morphologies and functions depending on their location in the brain [134–136]. Studies are needed to further determine whether other types of P-Mit may be different from T-Mit in terms of brain region of uptake since we consume more than one type plant-derived mitochondria from our daily meals. This may facilitate our understanding of P-Mit-based mechanisms and potential development of more effective treatments by targeting dysfunctional microglia located at specific brain subregions. We also noticed a higher uptake of T-Mit in the brain than in the spleen in old mice after gavage. This can be attributed to differences in vascular structure. The spleen has fenestrated capillaries that allow particles like T-Mit to pass through more easily while also having efficient mechanisms for filtering and removing particles. In contrast, the brain's vascular structure, especially when compromised by aging, may not be effective in clearing particles.

Consistent with hippocampal vulnerability to aging, T-Mit treatment significantly reduced neuronal loss and dying neurons in the hippocampus of aged mice, supporting a mitochondria-based neuroprotective strategy targeting conserved mechanisms of brain aging [137, 138]. As adult humans exhibit minimal hippocampal neurogenesis, preservation of existing neurons may represent the dominant approach to benefits [139]. However, longitudinal studies will be required to ensure that enhanced neuronal preservation will not impair necessary neuroimmune clearance [114].

Compared with human-derived mitochondrial transfer therapies, which face challenges regarding targeting, safety, immunogenicity, and scalability, edible P-Mit offers distinct translational advantages because it is naturally consumed, orally delivered, and unlikely to elicit immune rejection [140]. First, human-derived mitochondrial transfer therapy is complex, requiring precise control of mechanisms such as tunneling nanotubes, extracellular vesicles, and gap junction channels [4, 7, 23, 141]. Second, obtaining healthy mitochondria from donor cells and purifying them for transfer are technically demanding [4]. Third, the recipient's immune system may recognize the transferred mitochondria as foreign and mount an immune response, potentially leading to rejection [4]. Fourth, ensuring the safety of mitochondrial transfer is crucial, as improper transfer could lead to unintended consequences, such as the spread of mitochondrial diseases [4]. Last, the process of mitochondrial isolation, purification, and transfer is expensive, which could limit widespread application. Unlike the transferring of human-derived mitochondria, mitochondria from edible healthy plants are contained in all plants of diets. Using standard allometric scaling, we estimate that a daily human dose of approximately 2.4 g of purified P-Mit corresponds to the effective mouse regimen used in this study [142]. This provides a quantitative starting point for future translational studies. Our finding also provides the foundation for research to study the fate of edible P-Mit DNA after mitochondrial transfer. T-Mit sequencing data may address this concern.

This study has limitations. First, the mechanistic experiments relied on BV2 microglial cells in this study and validation in human iPSC-derived microglia is needed. Second, cell-type-specific distribution of P-Mit in the brain and the relative roles of BBB permeability versus metabolic demand remain to be determined. Third, the miRNA-mediated regulation remains to be further confirmed using sequence-specific antagonists and by disrupting mitochondrial import pathways.

Collectively, the present study demonstrates that the diet-derived mitochondria can remodel host mitochondrial function through plant–mammalian mitochondrial fusion, establishing a foundation for P-Mit transfer strategies targeting mitochondrial dysfunction in aging, neurodegeneration, metabolic disease, and cancer [116]. Based on these findings and the use of MitoTimer mice [4, 5, 112], we propose the development of a turmeric-derived MitoTimer system to study T-Mit distribution in recipient cells, which will be feasible once a comprehensive turmeric mitochondrial DNA sequencing database becomes available.

## Supplementary Information

Additional file 1. **Figure S1**. Isolation and characterization of dietary plant-derived mitochondria (P-Mit). **Figure S2**. P-Mit traffic into the aged brain with blood-brain barrier permeability impairment. **Figure S3**. Analysis of P-Mit distribution efficiency through gut-brain axial. **Figure S4**. Impact of T-Mit on Morris water maze test (MWM) in mice and verification of T-Mit recipient cells with immunofluorescence assay. **Figure S5**. P-Mit uptake by microglia via phagocytosis. **Figure S6**. Correlation analysis suggests P-Mit uptake by microglia is lipid dependent. **Figure S7**. Mitochondrial fusion between P-Mit and mitochondria in microglia/macrophage. **Figure S8**. T-Mit miRNAs inhibit the expression of ND4 and ND5.** Figure S9**. Analysis of T-Mit curcumin and toxicity.

Additional file 2. **Table S1**. Composition of lipids in plant-derived mitochondria (P-Mit) using LC-MS (%). **Table S2**. Composition of lipids in microglial mitochondria (M-Mit) and turmeric-microglial fused mitochondria (TM-Mit) using LC-MS (%). **Table S3**. Quantitative LC-MS/MS analysis of T-Mit complex I and its interaction with M-Mit complex I (Abundance). **Table S4** Turmeric mitochondrial (T-Mit) miRNAs sequencing raw counts. **Table** **S5**. Primer sequences used for qPCR

Additional file 3. Full uncropped gels and blots.

## Data Availability

All data generated or analyzed during this study are included in this published article and its Supplementary information files or provided in source data file. The microRNA sequencing data were deposited in the National Center for Biotechnology Information (NCBI) Gene Expression Omnibus (GEO) database with the accession number GSE165349 (https://www.ncbi.nlm.nih.gov/geo/query/acc.cgi?acc=GSE165349). The data that support the findings of this study are available on request from the corresponding author.

## Abbreviations

A-Mit: Aloe-derived mitochondria
ALT: Alanine aminotransferase
AST: Aspartate aminotransferase
ATP: Adenosine triphosphate
BBB: Blood–brain barrier
BV2: Murine microglial cell line
CI: Complex I
CII: Complex II
CIII: Complex III
CoQ: Ubiquinone
CoQH2: Ubiquinol
CSF: Cerebrospinal fluid
DGDG: Digalactosyldiacylglycerol
DiI: 1,1′-Dioctadecyl-3,3,3′,3′-tetramethylindocarbocyanine perchlorate
DiO: 3,3′-Dioctadecyloxacarbocyanine perchlorate
DiR: 1,1′-Dioctadecyltetramethyl indotricarbocyanine iodide
DRP1: Dynamin-related protein 1
ECAR: Extracellular acidification rate
EDS: Energy dispersive spectroscopy
ELISA: Enzyme-linked immunosorbent assay
ELN: Exosome-like nanoparticle
ETC: Electron transport chain
FACS: Fluorescence-activated cell sorting
FCCP: Carbonyl cyanide p-trifluoromethoxyphenylhydrazone
FET: Forward electron transport
Gin-Mit: Ginger-derived mitochondria
HPLC: High-performance liquid chromatography
IDH: Isocitrate dehydrogenase
IF: Immunofluorescence
IL: Interleukin
iNOS: Inducible nitric oxide synthase
KO: Knockout
LC–MS/MS: Liquid chromatography with tandem mass spectrometry
LPS: Lipopolysaccharide
M-Mit: Microglial mitochondria
MFN1: Mitofusin 1
MFN2: Mitofusin 2
MS: Mass spectrometry
mtROS: Mitochondrial reactive oxygen species
mtSOX: Mitochondrial superoxide
MWM: Morris water maze
NAO: Nonyl acridine orange
ND: NADH dehydrogenase
ND4: NADH dehydrogenase 4
ND5: NADH dehydrogenase 5
NF-κB: Nuclear factor kappa B
NOR: Novel object recognition
OCR: Oxygen consumption rate
OPA1: Optic atrophy 1
OXPHOS: Oxidative phosphorylation
PA: Phosphatidic acid
PATL: Patellin
PC: Phosphatidylcholine
PCA: Principal component analysis
PE: Phosphatidylethanolamine
PG: Phosphatidylglycerol
P-Mit: Plant-derived mitochondria
PLA: Proximity ligation assay
PS: Phosphatidylserine
qPCR: Quantitative polymerase chain reaction
RET: Reverse electron transport
RI: Refractive index
RNA-seq: RNA sequencing
ROS: Reactive oxygen species
SD: Standard deviation
SOX: Superoxide
SPR: Surface plasmon resonance
TCA: Tricarboxylic acid
TEM: Transmission electron microscopy
TFGβ: Transforming growth factor beta
TJ: Tight junction
TLC: Thin-layer chromatography
TM-Mit: Turmeric–microglial hybrid mitochondria
TMRE: Tetramethylrhodamine methyl ester
TNFα: Tumor necrosis factor alpha
T-Mit: Turmeric-derived mitochondria
TMSA: T-maze spontaneous alternation
T-NV: Turmeric-derived nanovesicle
TTFA: Thenoyltrifluoroacetone
TREM2: Triggering receptor expressed on myeloid cells 2
VDAC1: Voltage-dependent anion channel 1
WT: Wild type
