# Supplementary material for: Plant-derived mitochondria mitigate aging-related neurodegeneration by reprogramming microglial mitochondrial energy metabolism
Source: Transl Neurodegener. 2026 Jul 8;15:30. doi: 10.1186/s40035-026-00565-1 (PMC13343874; doi:10.1186/s40035-026-00565-1)
Supplement: Supplementary file 1 — Additional file 1. Figure S1. Isolation and characterization of dietary plant-derived mitochondria (P-Mit). Figure S2. P-Mit traffic into the aged brain with blood-brain barrier permeability impairment. Figure S3. Analysis of P-Mit distribution efficiency through gut-brain axial. Figure S4. Impact of T-Mit on Morris water maze test (MWM) in mice and verification of T-Mit recipient cells with immunofluorescence assay. Figure S5. P-Mit uptake by microglia via phagocytosis. Figure S6. Correlation analysis suggests P-Mit uptake by microglia is lipid dependent. Figure S7. Mitochondrial fusion between P-Mit and mitochondria in microglia/macrophage. Figure S8. T-Mit miRNAs inhibit the expression of ND4 and ND5. Figure S9. Analysis of T-Mit curcumin and toxicity. [file 40035_2026_565_MOESM1_ESM.docx]

Supplementary Materials for

**Plant-Microglial Mitochondrial Fusion Reverse Aging-related Neurodegeneration by Reprogramming Microglial Mitochondrial Energy Metabolism**

Yun Teng,^2†^* Chao Luo,^2,4†^ Qingbo Xu,^1†^ Jingyao Mu,^2^ Lucy Teng,^1,6^ Hongjia Qian,^2^ Yinan Huang,^2^ Minmin Liu,^2,5^ Lifeng Zhang,^2^ Juw Won Park,^2,7^ Jae Yeon Hwang,^2^ Maiying Kong,^2,7^ Jun Yan,^2^ Michael L Merchant,^8^ and Huang-Ge Zhang^1,2,3,9^*

Corresponding author: h0zhan17@louisville.edu (H.-G.Z.) and yun.teng@louisville.edu (Y.T.)

**The Supplementary Materials file includes:**

**Figures S1 to S9**


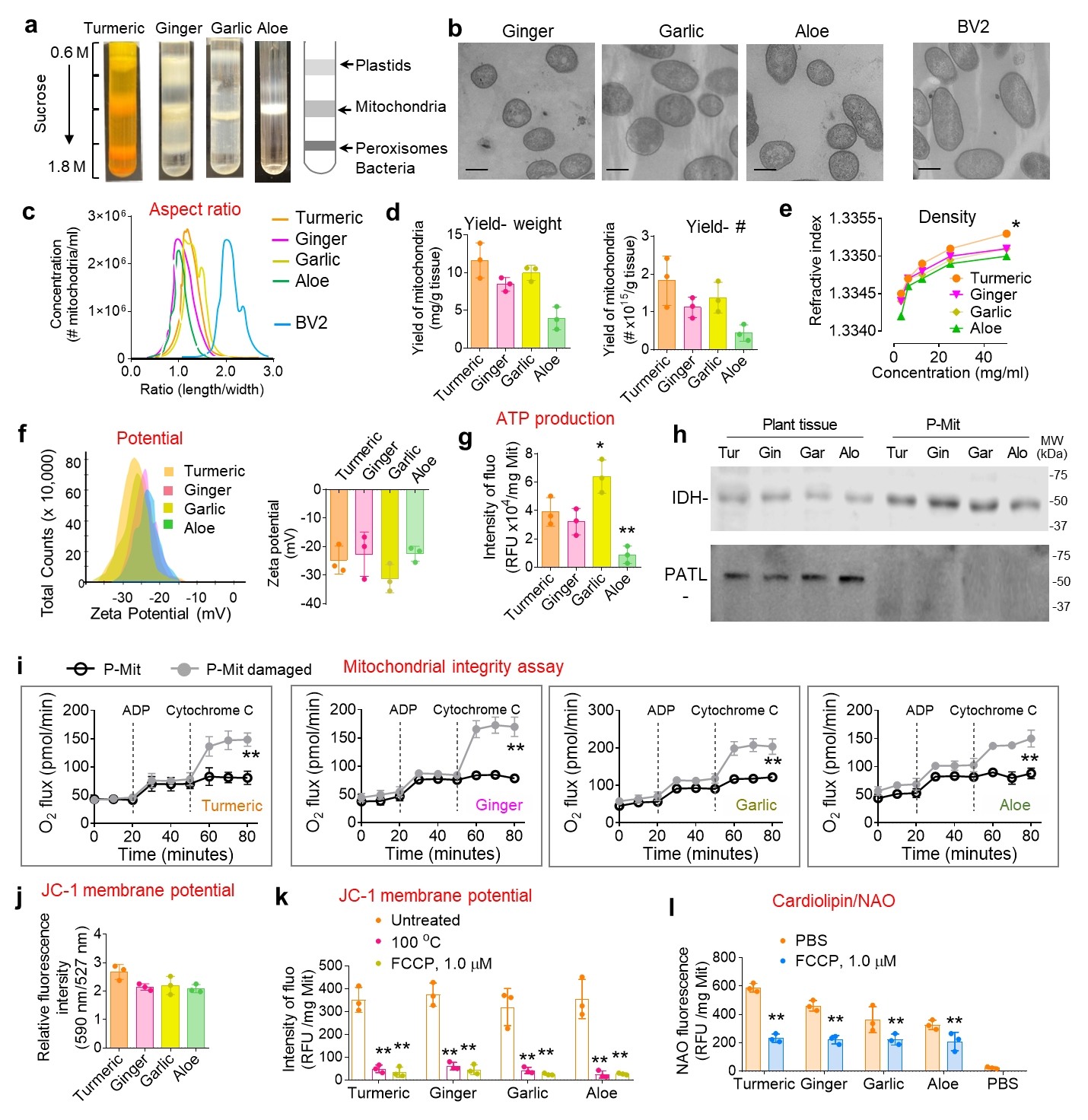
**Supplementary Figures and Figure Legends**

**Figure S1 Isolation and characterization of dietary plant-derived mitochondria (P-Mit). a** Purification of plant-derived mitochondria (P-Mit) from turmeric, ginger, garlic and aloe using sucrose gradient centrifugation with the concentration of sucrose indicated. **b** Representative transmission electron microscopy (TEM) images of P-Mit and microglial BV2 cell-derived mitochondria. Scale bars, 500 nm. **c**The aspect ratio (length/width in TEM) distribution of P-Mit. **d** Quantification of P-Mit yield by weight (left panel). Quantification of P-Mit yield by number of mitochondria (right panel). **e** Refractive index of P-Mit in different concentrations accessed using a refractometer. **f** A representative Zeta potential distribution of P-Mit estimated using the ZetaView with the laser wavelength set at 520 nm (left panel). Quantification of the Zeta potential of P-Mit (right panel). **g** The mitochondrial ATP level assessed with an ATP Assay Kit-Luminescence. **h** A representative western blot of IDH and PATL in plant whole tissue and P-Mit. **i** Integrity assessment of P-Mit. The induction of O_2_ flux indicated mitochondrial membrane deficiency mediated by cytochrome C. For positive control, P-Mit outer membrane disrupted by 1 % (v/v) Triton X-100. **j** Membrane potential of P-Mit assessed using JC-1 dye. Graph show fluorescence ratio of polarized mitochondria at 590 nm and depolarized mitochondrial at 527 nm. **k** P-Mit (0.1 g) in PBS heated at 100°C or incubated with FCCP (1.0 μM) for 5 min. Analysis of membrane potential at 590 nm using JC-1 dye. **l** Analysis of cardiolipin in P-Mit assessed using Nonyl Acridine Orange (NAO) fluorescent dye. Data are representative of three independent experiments (error bars, standard deviation (SD)). * *P* < 0.05, ** *P* < 0.01 (chi-square test for categorical variables; ANOVA along with post-hoc t-tests for continuous variables).

**
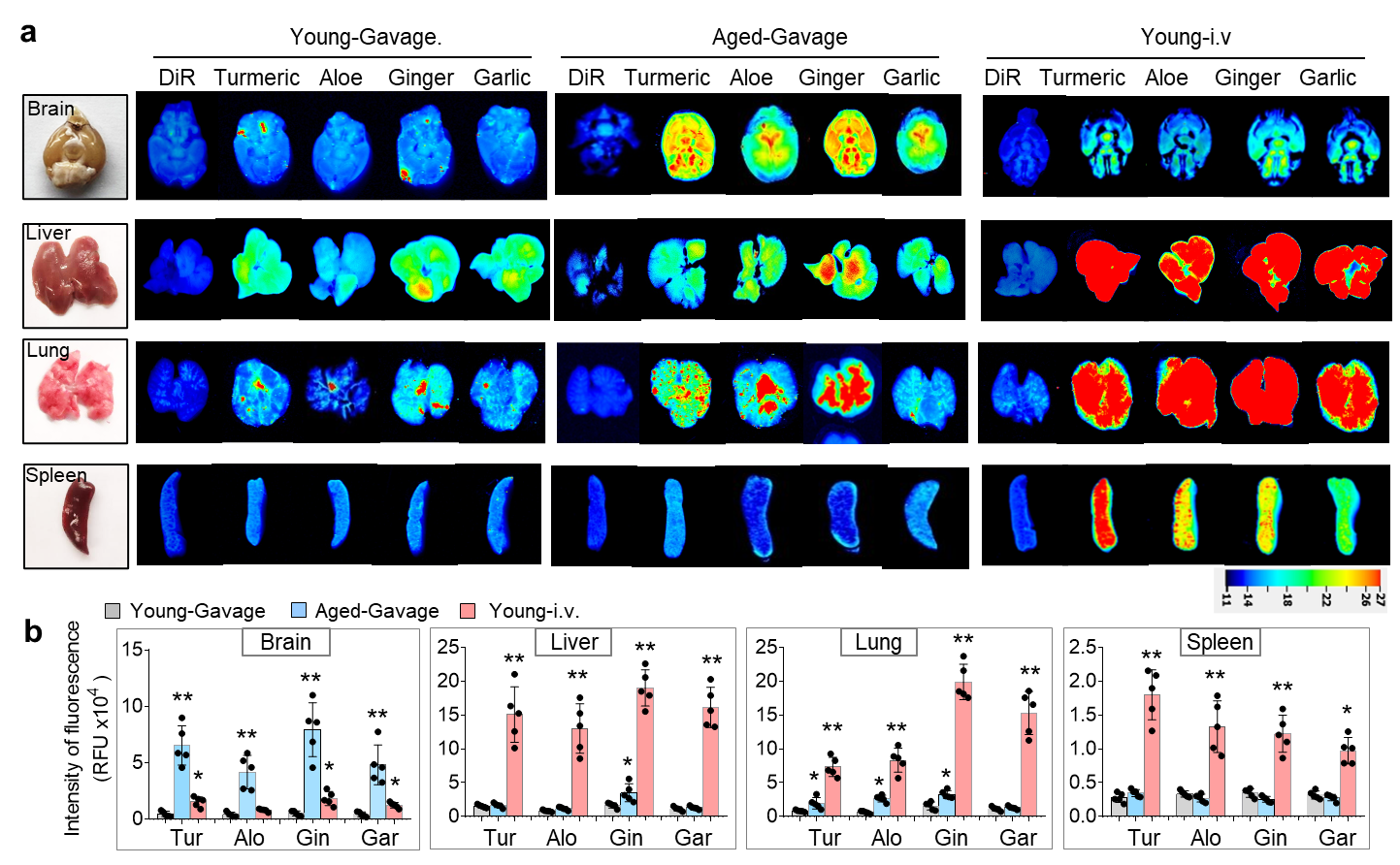
**

**Figure S2 P-Mit traffic into the aged brain with blood-brain barrier permeability impairment. a** A representative fluorescent image of brain, liver, lung and spleen from young and aged C57BL/6 mice (*n*=5) administered with a single gavage of 10 mg DiR dye-labeled P-Mit for 2 h (left and middle panel) or intravenous (i.v.) injection (right panel). **b**Quantification of T-Mit/DiR fluorescent intensity in (**a** ). * *P*  < 0.05, ** *P*  < 0.01 (two-tailed t-test) (vs Young-Gavage). Data are representative of three independent experiments (error bars, SD). * *P* < 0.05, ** *P* < 0.01, *** p *P*< 0.001, NS, not significant (chi-square test for categorical variables; ANOVA along with post-hoc t-tests for continuous variables).

**
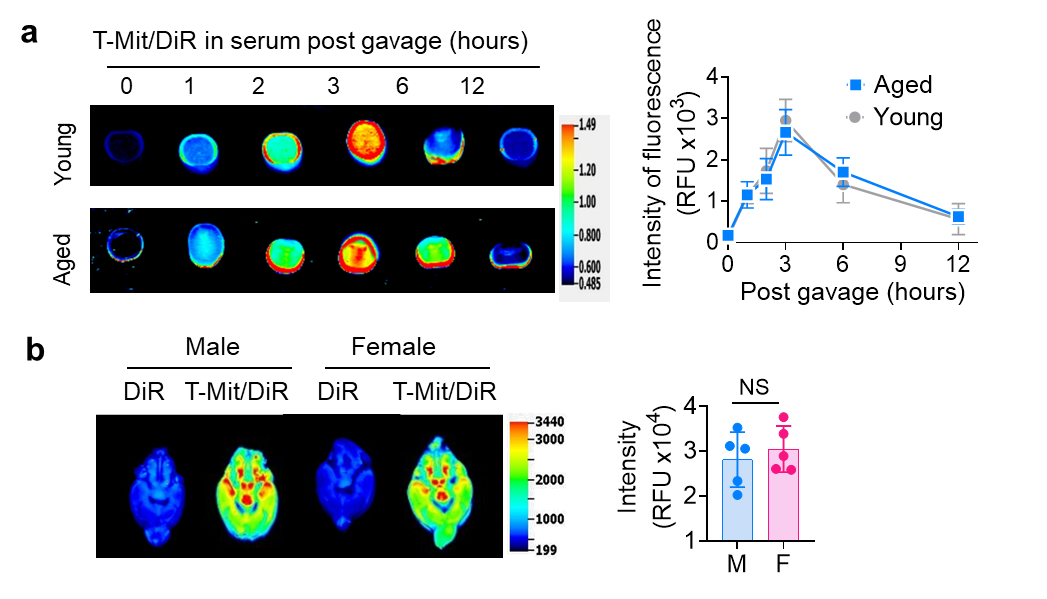
**

**Figure S3** **Analysis of P-Mit distribution efficiency through gut-brain axial. a** A representative image of fluorescence in serum (left panel) from young and aged mice administered with T-Mit/DiR via gavage (left panel) (*n*=5). Quantification of fluorescent intensity (right panel). **b** A representative image of fluorescence in brain (left panel) from male and female mice administered with T-Mit/DiR via gavage (*n*=5). Quantification of fluorescent intensity (right panel). Data are representative of three independent experiments (error bars, SD). NS, not significant (ANOVA along with post-hoc t-tests for continuous variables).

**
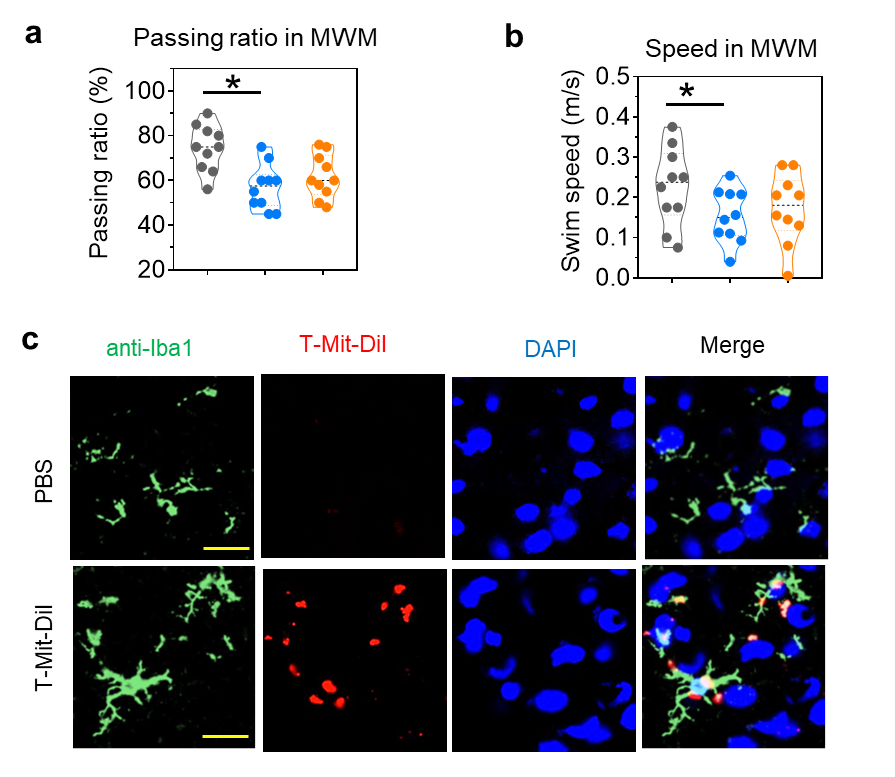
**

**Figure S4 Impact of T-Mit on Morris water maze test (MWM) in mice and verification of T-Mit recipient cells with immunofluorescence assay. a** Quantification of passing ratio in the MWM performance on day 5. Mice reaching the platform within 60 seconds were considered to have passed the test. **b** Quantification of swim speed in MWM performance. **c** T-Mit labeled with fluorescence dye DiI (red) and administered to aged mice by oral gavage (10mg/each, *n*=10). Colocalization analysis of microglia (Iba1^+^) and T-Mit/DiI in brain using IF with confocal microscopy 2h post gavage. The cell nuclei are counterstained with DAPI; Scale bars, 20 μm. Data are representative of three independent experiments (error bars, SD). * *P* < 0.05, (chi-square test for categorical variables; ANOVA along with post-hoc t-tests for continuous variables).

**
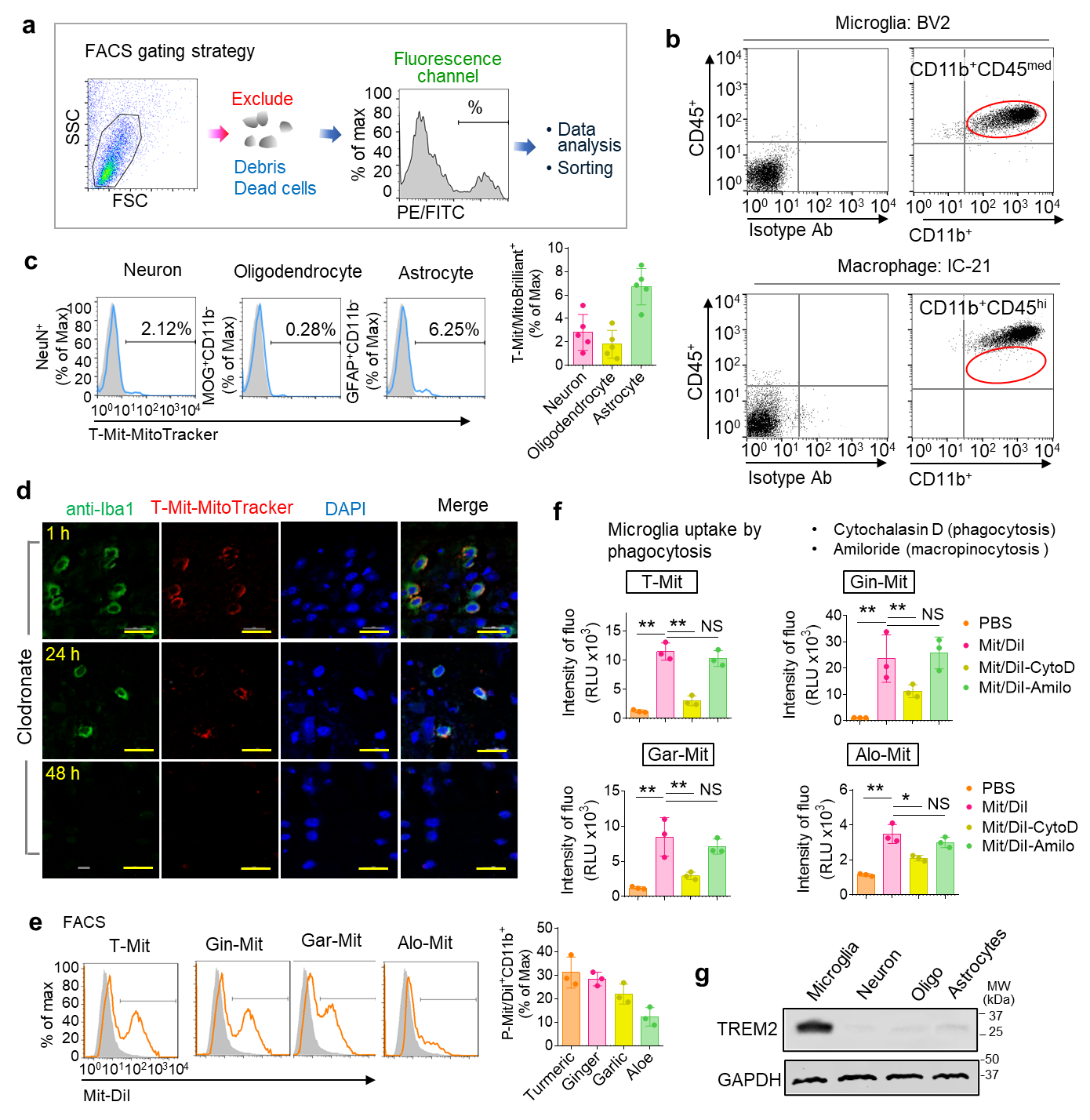
**

**Figure S5 P-Mit uptake by microglia via phagocytosis. a** Gating strategy in flow cytometry (FACS) analysis, forward versus side scatter (FSC vs SSC) gating used to identify cells of interest while excluding debris and dead cells, followed by gating signals in fluorescence channels such as PE and FITC for further analysis of cell markers or collection of cells. **b** FACS analysis of CD45 and CD11b in murine microglial BV2 cells and macrophage IC-21 cells. **c** Brain cells isolated from the mice fed with 10 mg T-Mit/MitoTracker. 2 h post gavage, the T-Mit/MitoTracker positive brain cells gated for recipient cell identification using FACS. Neuron, oligodendrocyte and astrocyte identified with their markers indicated (left panel), quantification of positive cells (right panel). **d** Microglia depleted from the aged mice (*n*=5) with clodronate (10 mg/kg, body weight) via intracranial injection. After 24 h and 48 h, T-Mit/MitoTracker (10 mg/each) administered by gavage followed by the IF analysis of the microglia (Iba1^+^) and T-Mit/MitoTracker in brain using confocal microscopy 2h post gavage. DAPI was used for the visualization of cell nuclei. Scale bars, 20 µm. **e** P-Mit isolated from turmeric (T-Mit), ginger (Gin-Mit), garlic (Gar-Mit) and aloe (Alo-Mit) and labeled with DiI. Microglia BV2 cells incubated with the P-Mit/DiI for 2 h. The percentage of P-Mit/Dil^+^ BV2 cells was determined by FACS analysis. **f** Microglia BV2 cells pretreated with the phagocytosis inhibitor cytochalasin D (CytoD, 10 µM) or micropinocytosis inhibitor amiloride (Amilo, 10 µM) for 2 h, subsequently incubated with the P-Mit/DiI for 2 additional hours. After washing with PBS, the fluorescence signal of P-Mit/Dil in BV2 cells measured using a microplate reader with an excitation at 549 nm and an emission at 565 nm. **g** Western blot analysis of TREM2 in major types of brain cells. Brain cells isolated from mice. The microglia, neurons, oligodendrocytes and astrocytes sorted and collected using FACS according to their markers such as CD11b^+^CD45^med^, NeuN^+^, MOG^+^CD11b^-^, and GFAP^+^CD45^-^CD11b^-^ respectively. Data are representative of three independent experiments (error bars, SD). * *P* < 0.05; ** *P* < 0.01, NS, not significant. (chi-square test for categorical variables; ANOVA along with post-hoc t-tests for continuous variables).

**
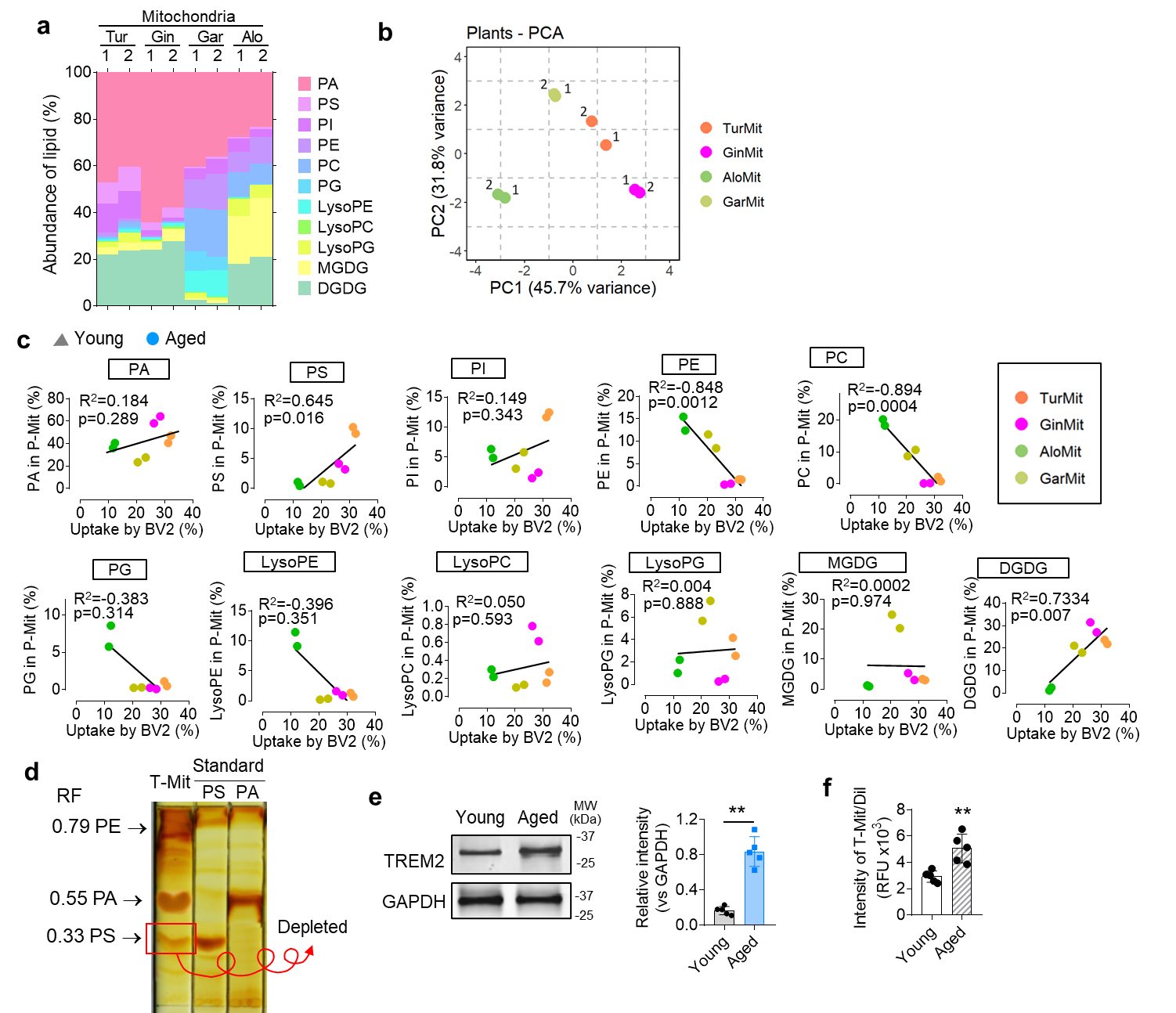
**

**Figure S6 Correlation analysis suggests P-Mit uptake by microglia is lipid dependent. a** Lipids isolated from P-Mit and the lipid composition identified by the liquid chromatography-mass spectrometry (LC-MS). **b** Principal component analysis (PCA) of lipid composition in P-Mit using software R. **c** Dot plots indicating Spearman’s correlation coefficients between the lipid composition of P-Mit and uptake efficiency of microglia. **d** Lipids of T-Mit separated by thin-layer chromatography (TLC) and identified by lipid standard based on the retention factor (RF). RF=distance of compound/distance of solvent, both measured from the origin. **e** A representative fluorescent image of brain from aged mice (*n*=5) fed with 10 mg DiR dye-labeled T-NV or T-NV/PS^del^ for 2 h. **f** Western blot analysis of TREM2 expression in microglia from young and aged mice. GAPDH used as a loading control (left panel); Quantification of band intensity (right panel). **g** Microglia isolated from young and aged mice and cultured with T-Mit/DiI in DMEM medium. After wash, the fluorescence engulfed by microglial cells was assessed using a microplate reader. Data are representative of three independent experiments (error bars, SD). ** *P* < 0.01, (chi-square test for categorical variables; ANOVA along with post-hoc t-tests for continuous variables).

**
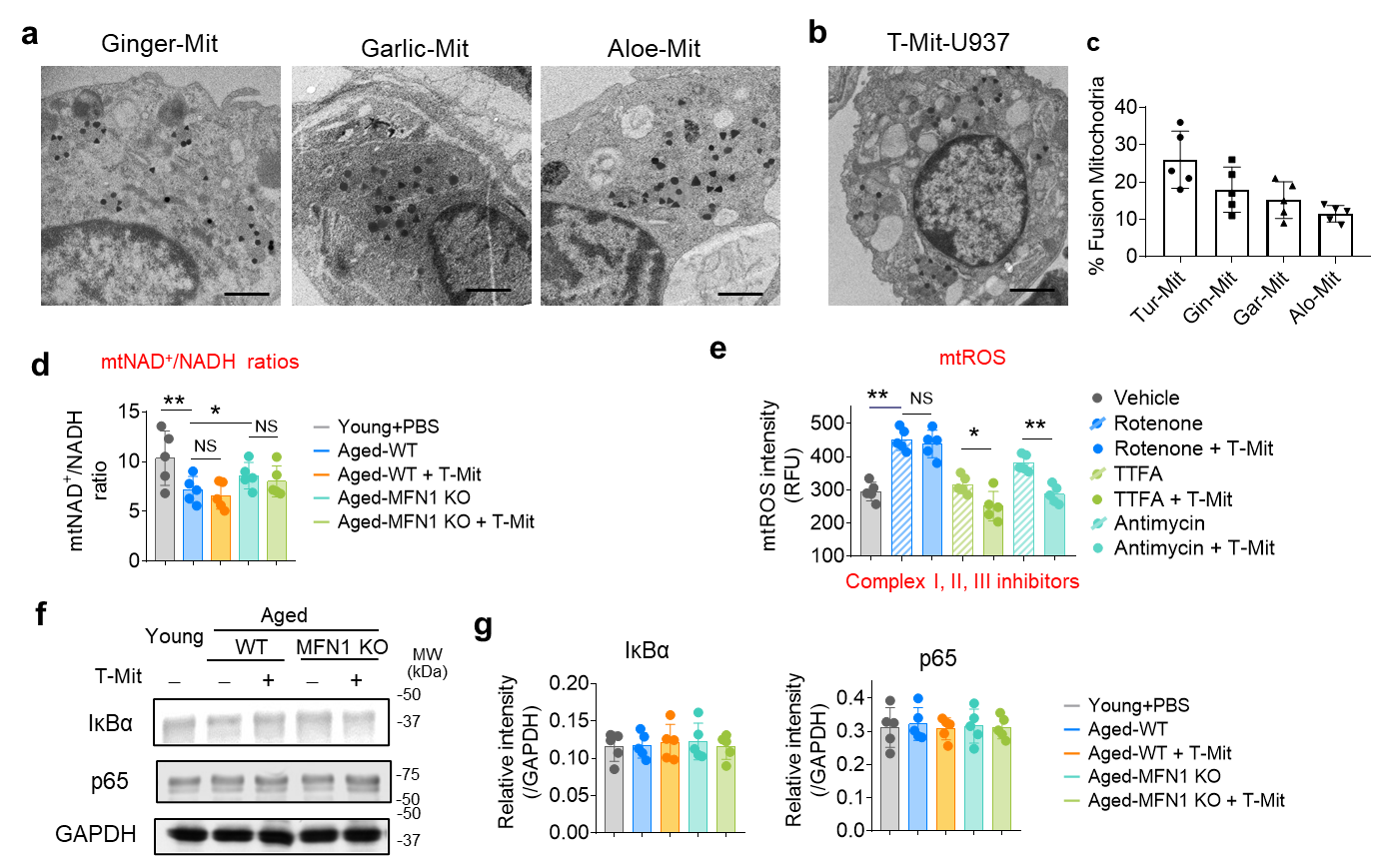
**

**Figure S7 Mitochondrial fusion between P-Mit and mitochondria in microglia/macrophage. a** BV2 cells incubated with Gold-Triangle labeled Gin-Mit, Gar-Mit and A-Mit. BV2 cell mitochondria labeled with anti-Tom20 antibody conjugated with Gold-Sphere identified by TEM. A representative TEM of fused mitochondria with Gold-Triangle^+^Gold-Sphere^+^ in BV2. Scale bars, 500 nm. **b** Human macrophage U937 cells incubated with Gin-Mit, Gar-Mit and A-Mit labeled with Gold-Triangle. Fused mitochondria in U937 visualized by TEM. **c** Quantification of fused mitochondria in U937 cells. **d** Primary microglia isolated from young, aged WT and aged MFN1 KO mice (*n*=5 per group) fed T-Mit for 2 m and grown in DMEM medium. mtNAD^+^/NADH assessed using a NAD^+^/NADH Assay Kit. **e** Analysis of ROS in primary microglia (1 x 10^5^) treated with complex I (rolenone, 0.1 mM), II (TTFA, 0.1 mM) and III inhibitors (antimycin A, 20 μM) 2 h followed by T-Mit (1 mg) treatment for 1 h. **f** Western blot analysis of total IκBα and p-65 in the primary microglia in Figure 6L. **g** Quantification of band intensity in (F). Data are representative of three independent experiments (error bars, SD). * *P*< 0.05, ** *P* < 0.01, NS, not significant (chi-square test for categorical variables; ANOVA along with post-hoc t-tests for continuous variables).

**
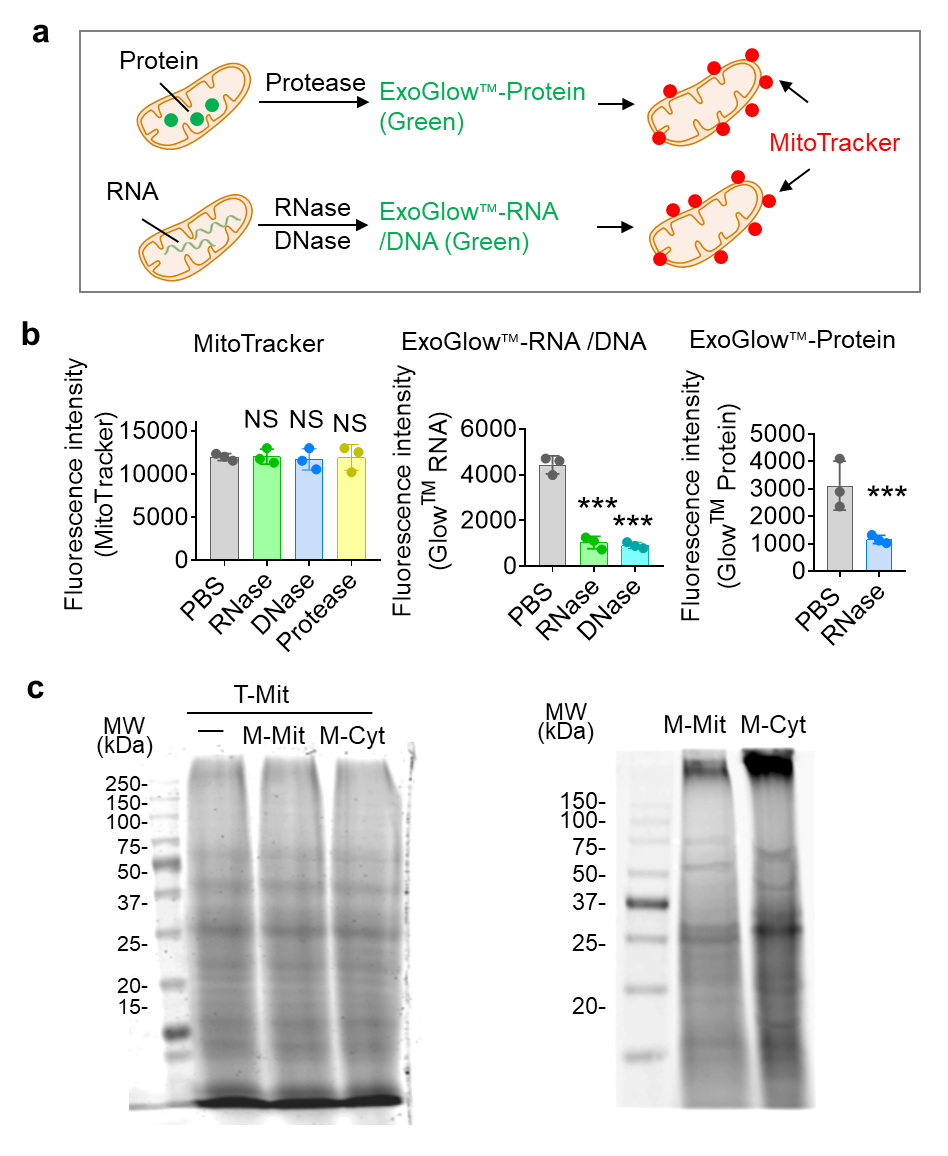
**

**Figure S8 T-Mit miRNAs inhibit the expression of ND4 and ND5. a** Schematic diagram of mitochondrial cargo protein, RNA and DNA removed by protease, RNase and DNase, respectively. mitochondrial membrane potential-based dye MitoTracker labeled mitochondrial membrane served as a control. **b** BV2 cells treated with protease (1.0 mg/ml) and RNase or DNase (2.0 mg/ml) at 37°C for 30 min. Mitochondrial cargo protein, RNA and DNA stained with ExoGlow™-Protein (Green) ExoGlow™-RNA or DNA (Green), respectively. Mitochondrial membrane labeled with MitoTracker served as a control. The fluorescence of MitoTracker, protein and RNA assessed using a microplate reader. **c** T-Mit complex I isolated and labeled with biotin followed by incubating with the protein extracted from M-Mit complex I (M-Mit) and microglial cytoplasm (M-Cyt). The Biotinylated T-Mit complex I-interacted proteins were pulled down by the streptavidin beads and separated by 10% SDS-PAGE. The protein bands visualized by Coomassie staining. Data are representative of three independent experiments (error bars, SD). * *P*< 0.05, ** *P* < 0.01, *** *P* < 0.001 (chi-square test for categorical variables; ANOVA along with post-hoc t-tests for continuous variables).

**
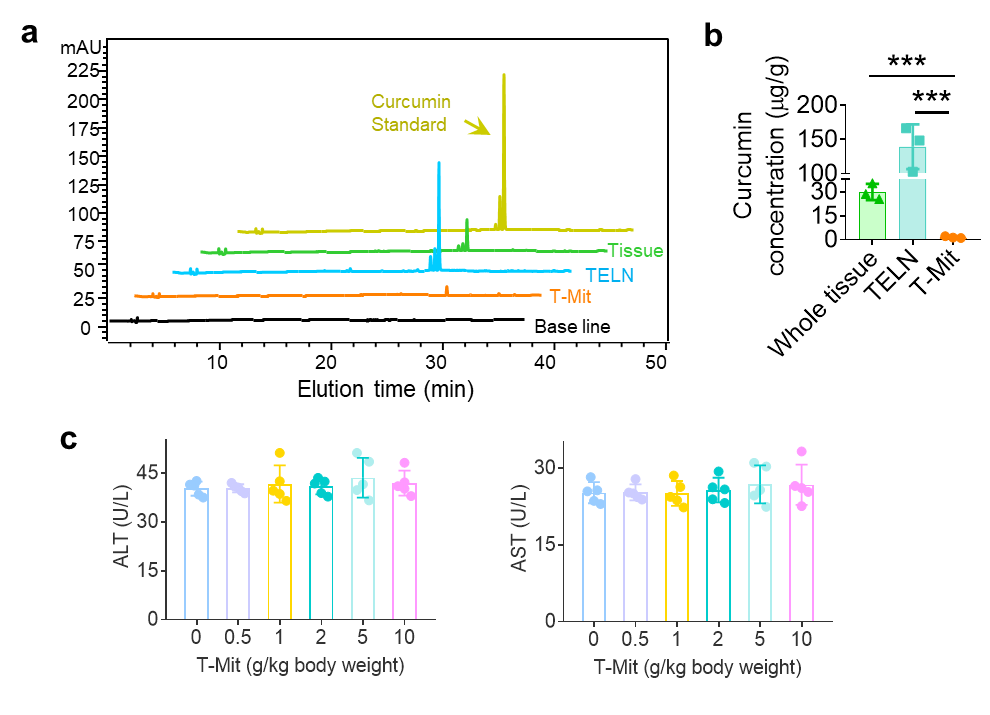
**

**Figure S9 Analysis of T-Mit curcumin and toxicity. a** Lipids extracted from turmeric tissue, T-Mit and turmeric-derived exosome-like nanoparticle (T-ELN). Identification of curcumin based on a curcumin standard (Sigma) using HPLC. **b**Quantification of curcumin based on the absorbance of a curcumin standard. **c** Analysis of T-Mit hepatoxicity. T-Mit gavage administered to aged mice for 2 m. The serum levels of alanine aminotransferase (ALT) (left panel) and aspartate aminotransferase (AST) (right panel) assessed with an infinity enzymatic agent. Data are representative of three independent experiments (error bars, SD). *** *P*< 0.001, (ANOVA along with post-hoc t-tests for continuous variables).
