# Supplementary material for: Plant-derived mitochondria mitigate aging-related neurodegeneration by reprogramming microglial mitochondrial energy metabolism
Source: Transl Neurodegener. 2026 Jul 8;15:30. doi: 10.1186/s40035-026-00565-1 (PMC13343874; doi:10.1186/s40035-026-00565-1)
Supplement: Supplementary file 2 — Additional file 2. Table S1. Composition of lipids in plant-derived mitochondria (P-Mit) using LC-MS (%). Table S2. Composition of lipids in microglial mitochondria (M-Mit) and turmeric-microglial fused mitochondria (TM-Mit) using LC-MS (%). Table S3. Quantitative LC-MS/MS analysis of T-Mit complex I and its interaction with M-Mit complex I (Abundance). Table S4 Turmeric mitochondrial (T-Mit) miRNAs sequencing raw counts. Table S5. Primer sequences used for qPCR [file 40035_2026_565_MOESM2_ESM.pdf]

Table S1 Composition of Lipids in Plan-derived Mitochondria (P-Mit) using LC-MS (%)

Name	Turmeric		Ginger		Garlic		Aloe	
	TurMit1	TurMit2	GinMit1	GinMit2	AloMit1	AloMit2	GarMit1	GarMit2
Phosphatidic acid (PA)	47.16	40.65	64.32	58.12	40.66	36.10	27.69	23.45
Phosphatidylserine (PS)	9.18	10.25	3.21	4.17	0.46	1.10	0.85	1.13
Phosphatidylinositol (PI)	12.45	11.65	2.39	1.46	4.86	6.32	5.79	3.05
Phosphatidylethanolamine (PE)	1.48	1.51	0.58	0.37	12.42	15.46	8.46	11.54
Phosphatidylcholine (PC)	0.84	2.15	0.25	0.16	18.18	20.12	10.61	8.65
Phosphatidylglycerol (PG)	0.51	1.11	0.11	0.26	8.57	5.76	0.32	0.27
LysoPE	0.72	1.32	0.92	1.60	9.13	11.46	0.35	0.19
LysoPC	0.27	0.16	0.62	0.79	0.22	0.30	0.13	0.10
LysoPG	2.55	4.16	0.49	0.27	2.19	1.02	7.45	5.66
Monogalactosyldiacylglycerol (MGDG)	2.97	3.37	3.04	5.33	0.89	1.25	20.37	24.89
Digalactosyldiacylglycerol (DGDG)	21.87	23.65	24.07	27.49	2.43	1.11	17.98	21.07

Each group was pooled from three independent samples.

Table S2 Composition of Lipids in Microglial Mitochondria (M-Mit) and Turmeric-Microglial Fused Mitochondria (TM-Mit) using LC-MS (%)

Name	M-Mit		TM-Mit	
	M-Mit1	M-Mit2	TM-Mit 1	TM-Mit 2
Phosphatidic acid (PA)	47.163	40.654	3.515	3.326
Phosphatidylserine (PS)	9.180	10.254	3.598	4.145
Phosphatidylinositol (PI)	12.447	11.654	3.737	4.215
Phosphatidylethanolamine (PE)	1.484	1.514	4.900	6.124
Phosphatidylcholine (PC)	0.841	2.154	71.558	65.846
Phosphatidylglycerol (PG)	0.511	1.114	3.797	4.654
LysoPE	0.718	1.322	3.845	5.121
LysoPC	0.273	0.157	0.199	0.354
LysoPG	2.547	4.158	3.938	5.154
Monogalactosyldiacylglycerol (MGDG)	2.969	3.365	0.353	0.215
Digalactosyldiacylglycerol (DGDG)	21.868	23.654	0.560	0.845

Each group was pooled from three independent samples.

Table S3 Quantitative LC-MS/MS analysis of T-Mit complex I and its interaction with M-Mit complex I (Abundance)

Description	Source	Gene Symbol	T-Mit	T-Mit+M-Mit	T-Mit+M-cyt	M-Mit	M-Cyt
ATP synthase subunit alpha, mitoch	plant		179.7	213.5	204.5	3.2	3.6
ATP synthase subunit beta OS=Cur	plant	atpB	172.6	219.2	202.4		
Coffee acyl coenzyme A-3-O-methy	plant		203.6	185.9	192.1	8.1	2.4
Curcumin synthase 3 OS=Curcuma	plant	CURS3	184.5	200.2	196.3	7.7	7.6
Curcumin synthase 1 OS=Curcuma	plant	CURS1	192.7	207.3	212.8		
Phenylalanine ammonia lyase (Frag	plant	PAL	164.6	183.3	201.3		
Type III polyketide synthase OS=Cu	plant	pks11	204.8	182.5	185.6	6.5	6.2
Serpin ZX (Fragment) OS=Curcuma	plant		189.3	193.8	202.9		
Protein TIC 214 OS=Curcuma longa	plant	ycf1	178.5	217.4	188.4	2.0	2.1
ATP-dependent Clp protease protea	plant	clpP	197.0	203.0	189.6		
Complex I assembly factor TIMMDC	Mouse	Timmdc1				223.2	176.8
NADH dehydrogenase [ubiquinone]	Mouse	Ndufaf4				202.7	197.3
NADH dehydrogenase [ubiquinone]	Mouse	Ndufa1				48.0	62.6
NADH dehydrogenase [ubiquinone]	Mouse	Ndufa10	6.3	3.8		192.3	197.7
NADH dehydrogenase [ubiquinone]	Mouse	Ndufa11				196.9	203.1
NADH dehydrogenase [ubiquinone]	Mouse	Ndufa12	3.5	1.8	2.8	160.5	159.7
NADH dehydrogenase [ubiquinone]	Mouse	Ndufa13	5.0	10.0	3.6	192.3	192.8
NADH dehydrogenase [ubiquinone]	Mouse	Ndufa2		4.3	2.2	199.5	196.3
NADH dehydrogenase [ubiquinone]	Mouse	Ndufa3				203.0	197.0
NADH dehydrogenase [ubiquinone]	Mouse	Ndufa5	2.5	3.6	3.3	141.3	148.6
NADH dehydrogenase [ubiquinone]	Mouse	Ndufa6				173.8	163.3
NADH dehydrogenase [ubiquinone]	Mouse	Ndufa7				171.5	171.8
NADH dehydrogenase [ubiquinone]	Mouse	Ndufa8				200.8	199.2
NADH dehydrogenase [ubiquinone]	Mouse	Ndufa9	10.8	13.3	11.4	192.4	183.5
NADH dehydrogenase [ubiquinone]	Mouse	Ndufb10				56.2	54.3
NADH dehydrogenase [ubiquinone]	Mouse	Ndufb11	28.8	30.8	20.5	173.0	167.4
NADH dehydrogenase [ubiquinone]	Mouse	Ndufb3	13.5	11.5	10.5	175.9	146.5
NADH dehydrogenase [ubiquinone]	Mouse	Ndufb4				65.8	104.6
NADH dehydrogenase [ubiquinone]	Mouse	Ndufb5	6.5			186.8	206.7
NADH dehydrogenase [ubiquinone]	Mouse	Ndufb6	16.3			191.3	192.3
NADH dehydrogenase [ubiquinone]	Mouse	Ndufb7				106.9	116.5
NADH dehydrogenase [ubiquinone]	Mouse	Ndufb8				193.2	206.8
NADH dehydrogenase [ubiquinone]	Mouse	Ndufb9	12.3	27.4	15.2	152.8	162.2
NADH dehydrogenase [ubiquinone]	Mouse	Ndufc2				208.2	191.8
Adaptin ear-binding coat-associated	Mouse	Necap1					400.0
Adipocyte plasma membrane-assoc	Mouse	Apmap					400.0
Agrin OS=Mus musculus OX=10090	Mouse	Agrn					400.0
Connector enhancer of kinase supp	Mouse	Cnksr2					400.0
Protein phosphatase 1 regulatory su	Mouse	Ppp1r1b					400.0
Transmembrane anterior posterior t	Mouse	Tapt1					400.0
CAAX prenyl protease 1 homolog O	Mouse	Zmpste24				102.0	298.0
Membrane protein MLC1 OS=Mus n	Mouse	Mlc1				104.0	296.0
Transmembrane protein 43 OS=Mu	Mouse	Tmem43				104.6	295.4
Leucine-rich repeat-containing prote	Mouse	Lrrc59				107.5	292.5
Platelet endothelial cell adhesion m	Mouse	Pecam1				115.0	285.0
Glutathione hydrolase 7 OS=Mus m	Mouse	Ggt7				117.2	282.8
Potassium channel subfamily K mer	Mouse	Kcnk1				118.5	281.5
Bifunctional purine biosynthesis pro	Mouse	Atic				123.9	276.1
MAP/microtubule affinity-regulating	Mouse	Mark3				126.5	273.5

Small nucleolar RNA host gene 4 (F	Mouse	Snhg4		128.7	271.3
GrpE protein homolog 1, mitochond	Mouse	Grpel1		133.0	267.0
Acid ceramidase OS=Mus musculus	Mouse	Asah1		134.9	265.1
Chromosome segregation 1 like OS	Mouse	Cse1l		139.6	260.4
Protein phosphatase PTC7 homolog	Mouse	Pptc7		140.1	259.9
Translationally-controlled tumor pro	Mouse	Tpt1		142.3	257.7
Calcium load-activated calcium cha	Mouse	Tmco1		143.0	257.0
Signal peptidase complex subunit 2	Mouse	Spcs2		143.3	256.7
SURF1-like protein (Fragment) OS=	Mouse	Surf1		143.4	256.6
Band 4.1-like protein 2 OS=Mus mu	Mouse	Epb41l2		143.5	256.5
Carboxypeptidase M OS=Mus musc	Mouse	Cpm		145.0	255.0
Muscle and microspikes RAS (Frag	Mouse	Mras		146.7	253.3
Hyaluronan and proteoglycan link p	Mouse	Hapln1		147.5	252.5
Protein XRP2 OS=Mus musculus O	Mouse	Rp2		148.3	251.7
Complexin-2 OS=Mus musculus OX	Mouse	Cplx2		151.3	248.7
Small nuclear ribonucleoprotein Sm	Mouse	Snrpd3		151.6	248.4
Cytochrome c oxidase subunit 1 OS	Mouse	Mtco1		152.3	247.7
Tripeptidyl-peptidase 2 OS=Mus mu	Mouse	Tpp2		153.1	246.9
Tripartite motif-containing 3 (Fragm	Mouse	Trim3		153.5	246.5
F-box only protein 2 OS=Mus musc	Mouse	Fbxo2		154.1	245.9
Oligosaccharyltransferase complex	Mouse	Ostc		154.3	245.7
Synaptotagmin-like 2 OS=Mus mus	Mouse	Sytl2		154.8	245.2
Prolactin regulatory element-binding	Mouse	Preb		155.7	244.3
Oxaloacetate tautomerase FAHD1,	Mouse	Fahd1		156.6	243.4
Mitochondrial import inner membran	Mouse	Timm23		157.2	242.8
Plasmolipin OS=Mus musculus OX=	Mouse	Pllp		158.6	241.4
NADH:ubiquinone oxidoreductase c	Mouse	Ndufaf2		159.6	240.4
Large ribosomal subunit protein uL4	Mouse	Mrpl4		160.1	239.9
Actin-related protein 2/3 complex su	Mouse	Arpc5l		160.3	239.7
Small ribosomal subunit protein mS	Mouse	Mrps34		160.6	239.4
Large neutral amino acids transport	Mouse	Slc7a8		160.7	239.3
ATP synthase F(0) complex subunit	Mouse	Atp5mg		160.9	239.1
Acyl-coenzyme A thioesterase 13 O	Mouse	Acot13		161.1	238.9
Beta-centractin OS=Mus musculus	Mouse	Actr1b	8.6	152.7	238.7
Nucleolin OS=Mus musculus OX=1	Mouse	Ncl		162.4	237.6
Acyl carrier protein (Fragment) OS=	Mouse	Ndufab1		163.0	237.0
Transmembrane 9 superfamily mem	Mouse	Tm9sf2		164.3	235.7
Acetylcholinesterase OS=Mus musc	Mouse	Ache		164.5	235.5
Inosine triphosphate pyrophosphata	Mouse	Itpa		164.6	235.4
FXYD domain-containing ion transp	Mouse	Fxyd6		165.1	234.9
Ciliary neurotrophic factor receptor	Mouse	Cntfr		165.2	234.8
Large ribosomal subunit protein uL1	Mouse	Rpl12		165.8	234.2
Inorganic pyrophosphatase 2, mitoc	Mouse	Ppa2		166.0	234.0
Thioredoxin-related transmembrane	Mouse	Tmx1		167.0	233.0
P2Y purinoceptor 12 OS=Mus musc	Mouse	P2ry12		167.5	232.5
Argininosuccinate synthase OS=Mu	Mouse	Ass1		167.6	232.4
Mitochondrial import inner membran	Mouse	Timm44		167.6	232.4
Neurotrophic tyrosine kinase, recep	Mouse	Ntrk2		167.6	232.4
Adenylate cyclase type 3 OS=Mus m	Mouse	Adcy3		167.9	232.1
Synaptobrevin homolog YKT6 OS=M	Mouse	Ykt6		168.4	231.6
Serine/threonine-protein phosphata	Mouse	Ppp1ca		169.2	230.8
Calcium-binding protein 39 OS=Mus	Mouse	Cab39		169.5	230.5

*Each group was pooled from three independent samples.

Table S4 Turmeric Mitochondrial (T-Mit) miRNAs Sequencing Raw Counts

miR_name	miR_seq	Length	GenomeID	CG%	T-Mit1	T-Mit2
peu-MIR2916-p3_2ss14TC17	GCCGACCAGGGATCGGTGGATGTT	24	WXZB01001616.1	50.6	6640	5246
osa-miR159a.1_2ss20TC21G	TTTGGATTGAAGGGAGCTCCC	21	WXZB01282026.1	50.8	6588	4961
tae-miR319_R-1	TTGGACTGAAGGGAGCTCCC	20	WXZB01007556.1	54.8	5796	3846
peu-MIR2916-p5_1ss6AG	ATACCGTCCTAGTCTCAACCATA	23	WXZB01001616.1	50.6	5005	4090
osa-miR535-5p	TGACAACGAGAGAGAGCACGC	21	WXZB01075556.1	52.8	4273	3042
osa-miR166a-3p	TCGGACCAGGCTTCATTCCCC	21	WXZB01075556.1	46.2	4247	3182
sbi-miR156a_L+1	TTGACAGAAGAGAGTGAGCAC	21	WXZB01075424.1	49.0	4200	3264
PC-3p-100_7893	CTTCCAATTCCTCCCATTCCGA	22	WXZB01370281.1	49.1	4176	3800
osa-miR164a	TGGAGAAGCAGGGCACGTGCA	21	WXZB01000756.1	40.4	3620	2963
tae-miR319_1ss20CT	TTGGACTGAAGGGAGCTCCTT	21	WXZB01041941.1	45.4	3013	2160
peu-miR2916_R-3_1ss6AG	TGGGGGCTCGAAGACGATCA	20	WXZB01005846.1	51.2	2683	1773
aof-miR319b_2ss20CT21TA	TTTGGACTGAAGGGAGCTCTA	21	WXZB01075556.1	46.1	2002	1548
aof-miR319b_L-1	TTGGACTGAAGGGAGCTCCT	20	WXZB01055766.1	37.2	1324	831
vca-miR156b-5p	TTGACAGAAGATAGAGAGCAC	21	WXZB01007556.1	47.8	1302	1096
osa-miR528-5p	TGGAAGGGGCATGCAGAGGAG	21	WXZB01032938.1	50.0	1002	762
bdi-MIR319a-p5	CTTGGACTGAAGGGAGCTCCC	21	WXZB01033222.1	50.0	919	715
tae-miR319	TTGGACTGAAGGGAGCTCCCT	21	WXZB01033222.1	50.0	911	678
tae-miR319	TTGGACTGAAGGGAGCTCCCT	21	WXZB01075556.1	49.2	911	678
ppe-miR156c	TGACAGAAGAGAGTGAGCAC	20	WXZB01007556.1	51.8	756	546
nta-miR156a	TGACAGAAGAGAGTGAGCAC	20	WXZB01075556.1	48.6	756	546
osa-miR162b_2ss8GA20AG	TCGATAAACCTCTGCATCCGG	21	WXZB01000756.1	50.3	748	678
osa-miR162a_1ss20AG	TCGATAAACCTCTGCATCCGG	21	WXZB01007556.1	47.2	748	678
osa-miR535-5p_2ss1TC21CT	CGACAACGAGAGAGAGCACGT	21	WXZB01007556.1	50.7	737	499
zma-MIR172b-p3_2ss14CG17	ATGATGCGTGCCTGGCCT	18	WXZB01007556.1	60.3	508	324
zma-MIR172b-p5_2ss14CG17	ATGATGCGTGCCTGGCCT	18	WXZB01621694.1	62.5	508	324
osa-miR396a-5p	TTCCACAGCTTTCTTGAACTG	21	WXZB01007556.1	45.7	485	375
osa-miR396a-5p	TTCCACAGCTTTCTTGAACTG	21	WXZB01017227.1	43.4	485	375
osa-miR396e-5p	TCCACAGGCTTTCTTGAACTG	21	WXZB01000756.1	44.0	457	365
osa-miR396e-5p	TCCACAGGCTTTCTTGAACTG	21	WXZB01007556.1	47.6	457	365
osa-miR159f_1ss1CT	TTTGGATTGAAGGGAGCTCTA	21	WXZB01075556.1	38.0	454	352
osa-miR168a-5p_2ss14AG21	TCGCTTGGTGCAGGTCGGGAA	21	WXZB01013055.1	65.6	323	255
aof-miR168b_2ss20TA21CA	TCGCTTGGTGCAGGTCGGGAA	21	WXZB01075556.1	65.3	323	255
osa-miR169n_1ss21AC	TAGCCAAGAATGACTTGCCTC	21	WXZB01029035.1	44.3	301	206
osa-miR169n_1ss21AC	TAGCCAAGAATGACTTGCCTC	21	WXZB01043720.1	45.0	301	206
gra-MIR8689-p5_2ss5AT18CT	AAGTTGTGCCCGAGGCCT	18	WXZB01075556.1	69.4	295	195
gra-MIR8689-p5_2ss5AT18CT	AAGTTGTGCCCGAGGCCT	18	WXZB01725324.1	63.8	295	195
gra-miR482_L-2R+1_1ss3TC	CTTCCAATTCCTCCCATTCCG	21	WXZB01370281.1	49.1	294	223
osa-miR5179	TTTTGCTCAAGACCGCGCAAC	21	WXZB01001192.1	51.9	291	205
osa-miR5179	TTTTGCTCAAGACCGCGCAAC	21	WXZB01015718.1	51.7	291	205
tae-miR319_L+1R-1_1ss21CT	CTTGGACTGAAGGGAGCTCCT	21	WXZB01075556.1	44.2	284	204
osa-miR164c_1ss17GA	TGGAGAAGCAGGGTACATGCA	21	WXZB01007556.1	52.7	235	211
osa-miR171h_L-1R+1_1ss2T	CGAGCCGAACCAATATCACTC	21	WXZB01003824.1	41.8	214	151
mes-miR171l	CGAGCCGAACCAATATCACTC	21	WXZB01127780.1	36.2	214	151
zma-miR164g-3p_1ss20CA	CACGTGCTCCCCTTCTCCAAC	21	WXZB01169433.1	54.8	189	144
osa-miR164d_2ss19GT20CT	TGGAGAAGCAGGGCACGTTTT	21	WXZB01009392.1	61.0	178	114
PC-3p-5630_254	CTCACTGCTCTGTCTGTCATCGC	23	WXZB01030497.1	61.7	152	102
ath-MIR5652-p5_2ss2GA17AT	TAACAATATCGGGCTCTT	18	WXZB01442395.1	34.0	147	71
osa-miR394_1ss8TC	TTGGCATCCTGTCCACCTCC	20	WXZB01007556.1	46.2	131	84
osa-miR394_1ss8TC	TTGGCATCCTGTCCACCTCC	20	WXZB01755556.1	55.6	131	84
osa-miR396a-5p_1ss20TG	TTCCACAGCTTTCTTGAACGG	21	WXZB01075556.1	46.8	125	105
stu-miR171b-3p	TTGAGCCGCGTCAATATCTCT	21	WXZB01497344.1	51.0	117	100
PC-5p-11080_137	CGCGTGCGCGGCCCCTGCC	19	WXZB01075556.1	62.9	117	114
osa-MIR395l-p3_2ss12TG18C	CTTTTGTGAAGGGTTTGT	18	WXZB01075556.1	48.4	103	80

osa-miR164a_1ss21AT	TGGAGAAGCAGGGCACGTGCT	21	WXZB01169433.1	54.8	98	75
osa-miR164a_1ss21AT	TGGAGAAGCAGGGCACGTGCT	21	WXZB01577062.1	57.1	98	75
cme-MIR160c-p3_2ss13AG17	AGCCATGCATGTGTAAGT	18	WXZB01226035.1	37.2	95	58
cme-MIR160c-p5_2ss13AG17	AGCCATGCATGTGTAAGT	18	WXZB01755556.1	37.5	95	58
osa-miR167d-5p_R+1	TGAAGCTGCCAGCATGATCTGA	22	WXZB01123706.1	45.4	92	73
osa-miR167d-5p_R+1	TGAAGCTGCCAGCATGATCTGA	22	WXZB01236895.1	50.0	92	73
osa-miR396e-5p_R-1	TCCACAGGCTTTCTTGAACT	20	WXZB01032849.1	37.9	91	79
aof-miR319b_R-1_1ss20CT	TTTGGACTGAAGGGAGCTCT	20	WXZB01696177.1	36.7	91	58
mtr-MIR2592bj-p5_2ss12TC1	ATTCCCACTGTCCCTGTCT	19	WXZB01148958.1	54.5	89	36
mtr-MIR2592bj-p5_2ss12TC1	ATTCCCACTGTCCCTGTCT	19	WXZB01292177.1	54.5	89	36
gma-miR159a-5p_1ss19TA	GAGCTCCTTGAAGTCCAAATG	21	WXZB01490509.1	55.9	88	99
gma-MIR5368-p5_1ss13CT	TCTAACCTTGTGTCAGGACCT	21	WXZB01755556.1	51.0	82	73
gma-MIR5368-p3_1ss13CT	TCTAACCTTGTGTCAGGACCT	21	WXZB01313020.1	54.9	82	73
PC-5p-19266_73	GTAGGGCCGAGTGGTCGGA	19	WXZB01075556.1	50.9	81	65
tae-MIR171b-p3	CGATTGAGCCGTGCCAATATC	21	WXZB01075556.1	42.2	77	74
tae-MIR171b-p3	CGATTGAGCCGTGCCAATATC	21	WXZB01216516.1	39.4	77	74
osa-miR5077_L-1_1ss5GA	TTCACGTCGGGTTCACCA	18	WXZB01216516.2	59.3	76	60
bdi-miR156b-5p_L+2R-1	TTTGACAGAAGAGAGTGAGCA	21	WXZB01171253.1	44.7	76	57
bdi-miR156b-5p_L+2R-1	TTTGACAGAAGAGAGTGAGCA	21	WXZB01205222.1	45.2	76	57
gra-miR482_L-2_1ss3TC	CTTCCAATTCCTCCCATTCC	20	WXZB01075556.1	51.4	75	44
osa-miR166a-3p_L+2R-2	TCTCGGACCAGGCTTCATTCC	21	WXZB01000756.1	50.8	73	57
osa-miR166a-3p_L+2R-2	TCTCGGACCAGGCTTCATTCC	21	WXZB01075556.1	50.5	73	57
tae-miR319_1ss21TC	TTGGACTGAAGGGAGCTCCCC	21	WXZB01007556.1	42.0	71	50
cst-MIR11334-p3_2ss9TG18T	TAAGGAGTGTGTAACAAC	18	WXZB01755556.1	44.6	63	43
cst-MIR11334-p3_2ss9TG18T	TAAGGAGTGTGTAACAAC	18	WXZB01755556.1	48.5	63	43
PC-5p-19620_71	CCGTTGCTCGACTTGGGG	18	WXZB01075556.1	70.5	63	47
osa-miR167a-5p_1ss21AC	TGAAGCTGCCAGCATGATCTC	21	WXZB01053802.1	35.3	61	42
vvi-miR167c	TGAAGCTGCCAGCATGATCTC	21	WXZB01066056.1	38.2	61	42
gma-MIR5368-p3_1ss17CA	GAGATACCACTCTGGAAGAGCT	22	WXZB01007556.1	50.0	61	27
gma-MIR5368-p5_1ss17CA	GAGATACCACTCTGGAAGAGCT	22	WXZB01075556.1	44.9	61	27
osa-miR172a	AGAATCTTGATGATGCTGCAT	21	WXZB01025063.1	41.9	59	44
osa-miR172a	AGAATCTTGATGATGCTGCAT	21	WXZB01075556.1	45.7	59	44
cst-MIR11334-p5_2ss9TG18T	TAAGGAGTGTGTAACAACT	19	WXZB01075556.1	43.5	59	41
cst-MIR11334-p5_2ss9TG18T	TAAGGAGTGTGTAACAACT	19	WXZB01094132.1	43.5	59	41
ath-MIR858a-p3_2ss4GA20T(	TTCATTGTCTGTTCGACCTCG	21	WXZB01075556.1	47.3	56	37
ath-MIR858a-p5_2ss4GA20T(	TTCATTGTCTGTTCGACCTCG	21	WXZB01075556.1	33.3	56	37
osa-miR159a.1_R-1	TTTGGATTGAAGGGAGCTCT	20	WXZB01001568.1	50.0	55	31
mdm-MIR171m-p5_2ss2GA2(	TATTGGAATGGCTCAATCAGA	21	WXZB01075556.1	43.0	54	51
PC-3p-15195_98	CCTGTGCCTGCCCTTTCCACT	21	WXZB01032938.1	50.0	52	52
osa-miR166a-3p_1ss17TC	TCGGACCAGGCTTCATCCCCC	21	WXZB01001458.1	38.6	52	44
osa-miR166a-3p_1ss17TC	TCGGACCAGGCTTCATCCCCC	21	WXZB01030738.1	43.0	52	44
ppe-MIR159-p5_1ss9TA	CTTGCATAACTCAGGAGCTGC	21	WXZB01075556.1	46.1	46	22
mdm-MIR159d-p3_1ss9TA	CTTGCATAACTCAGGAGCTGC	21	WXZB01262466.1	48.9	46	22
ptc-miR6478_1ss21GA	CCGACCTTAGCTCAGTTGGTA	21	WXZB01115226.1	44.4	46	29
ptc-miR6478_1ss21GA	CCGACCTTAGCTCAGTTGGTA	21	WXZB01638076.1	57.6	46	29
osa-MIR156i-p3	CTCACTGCTCTGTCTGTCATC	21	WXZB01167676.1	59.1	45	49
bdi-miR156d-3p_L-1_1ss21C1	CTCACTGCTCTGTCTGTCATC	21	WXZB01075556.1	63.5	45	49
mtr-miR319d-5p_2ss7CT8TC	AGAGCTTCCTTCAGTCCACTC	21	WXZB01075556.2	40.3	45	36
osa-miR396a-5p_R-1	TTCCACAGCTTTCTTGAACT	20	WXZB01049196.1	37.4	44	36
osa-miR396a-5p_R-1	TTCCACAGCTTTCTTGAACT	20	WXZB01061160.1	46.5	44	36
cca-MIR168a-p3_2ss7TA18G	CGGTGGATGTTGCTCTTA	18	WXZB01019147.1	48.4	42	35
osa-miR396a-5p_R+1_2	TTCCACAGCTTTCTTGAACTGC	22	WXZB01027443.1	39.5	39	26
osa-miR396a-5p_R+1_1	TTCCACAGCTTTCTTGAACTGA	22	WXZB01042995.1	44.4	39	26
gma-MIR5368-p3_1ss7TC	ACTGTTCCCTGGGATTGGCT	20	WXZB01007556.1	51.5	38	24
gma-MIR5368-p3_1ss7TC	ACTGTTCCCTGGGATTGGCT	20	WXZB01755556.1	47.5	38	24

cca-MIR6105b-p3_1ss9TG	TGTGAAGGGTTTGTGTTG	18	WXZB01075556.1	56.2	36	14
PC-3p-14436_103	CGGTTCCCCACTTGCATCAAG	21	WXZB01007556.1	56.8	35	35
PC-3p-14436_103	CGGTTCCCCACTTGCATCAAG	21	WXZB01110970.1	59.3	35	35
osa-miR393a_L+1	TTCCAAAGGGATCGCATTGATC	22	WXZB01001688.1	42.2	34	26
osa-miR393a_L+1	TTCCAAAGGGATCGCATTGATC	22	WXZB01231374.1	40.1	34	26
osa-MIR395l-p5_2ss12TG18C	CTTTTGTGAAGGGTTTGTG	19	WXZB01075556.1	48.9	34	33
osa-miR172a_1ss21TC	AGAATCTTGATGATGCTGCAC	21	WXZB01004340.1	47.3	31	16
osa-miR172a_1ss21TC	AGAATCTTGATGATGCTGCAC	21	WXZB01043375.1	43.7	31	16
PC-5p-25879_47	TTTGAACTTTAGACCTGCAGG	21	WXZB01508473.1	43.7	30	28
osa-miR535-5p_1ss2GN	TNACAACGAGAGAGAGCACGC	21	WXZB01075247.1	54.8	28	17
sbi-miR160a	TGCCTGGCTCCCTGTATGCCA	21	WXZB01032626.1	55.3	27	7
bdi-MIR1432-p5_1ss4TC	TGTCCAGGAGAGATGACACCG	21	WXZB01188071.1	44.2	27	24
gma-miR6300_R+1	GTCGTTGTAGTATAGTGGT	19	WXZB01075556.1	46.7	27	10
PC-5p-25026_50	CCCAGATTTGAACTTTAGACC	21	WXZB01075556.1	45.9	27	23
PC-3p-32917_31	GTGCTCTTCCTCGTTGTCACC	21	WXZB01075247.1	54.8	27	17
peu-MIR2916-p5_2ss2AG17T	GGCTCGAAGACGATCAGAT	19	WXZB01019147.1	48.4	27	20
osa-miR535-5p_1ss9AC	TGACAACGCGAGAGAGCACGC	21	WXZB01306906.1	54.4	24	452
bdi-miR156d-3p_L-1R+1_1ss	CTCACTGCTCTGTCTGTCATCG	22	WXZB01030497.1	61.7	23	18
osa-miR171b	TGATTGAGCCGTGCCAATATC	21	WXZB01075556.1	35.8	23	19
bdi-MIR7771-p3_2ss8TA17TA	TTTGGTAATTGGAATGAGT	19	WXZB01442395.1	34.0	23	17
bdi-MIR7771-p3_2ss8TA17TA	TTTGGTAATTGGAATGAGT	19	WXZB01126507.1	31.0	23	17
bdi-MIR7771-p3_2ss8TA17TA	TTTGGTAATTGGAATGAGTAC	21	WXZB01001616.1	32.5	23	17
osa-miR530-5p_R+1	TGCATTTGCACCTGCACCTAA	21	WXZB01048330.1	43.1	23	20
osa-miR530-5p_R+1	TGCATTTGCACCTGCACCTAA	21	WXZB01054822.1	41.3	23	20
osa-miR396a-3p_L-1R+1	TTCAATAAAGCTGTGGGAAA	20	WXZB01027443.1	39.5	22	14
osa-miR396a-3p_L-1R+1	TTCAATAAAGCTGTGGGAAA	20	WXZB01049196.1	37.4	22	14
osa-miR166a-3p_1ss21CA	TCGGACCAGGCTTCATTCCCA	21	WXZB01075556.1	64.4	22	8
mtr-miR171c	TGATTGAGCCGTGCCAATATT	21	WXZB01075556.1	43.0	22	15
mtr-miR171c	TGATTGAGCCGTGCCAATATT	21	WXZB01411714.1	36.7	22	15
aof-miR168a_R+1	TCGCTTGGTGCAGGTCGGGAAC	22	WXZB01004531.1	63.3	21	14
osa-miR171h_L-1R+1	TGAGCCGAACCAATATCACTC	21	WXZB01038002.1	46.7	21	12
gma-MIR4995-p5	CTCATAGGCAGTGGCTTGGTT	21	WXZB01007556.1	53.4	21	10
gma-MIR4995-p3	CTCATAGGCAGTGGCTTGGTT	21	WXZB01735388.1	44.1	21	10
gma-MIR5368-p3_1ss3GA	CCAAGGGACAGTCTCAGGTA	20	WXZB01755556.1	51.0	21	13
gma-MIR5368-p5_1ss3GA	CCAAGGGACAGTCTCAGGTA	20	WXZB01000254.1	57.1	21	13
zma-miR156e-3p_L+1_1ss15	TGCTCACTGCTCTCGCTGTCATC	23	WXZB01007556.1	55.5	20	29
osa-miR164a_R-1	TGGAGAAGCAGGGCACGTGC	20	WXZB01075556.1	50.8	20	20
aof-miR169b_L+1R-1_1ss11C	TTAAGCCAAGAATGACTTGCC	21	WXZB01027345.1	45.2	20	11
aof-miR169b_L+1R-1_1ss11C	TTAAGCCAAGAATGACTTGCC	21	WXZB01046492.1	44.0	20	11
osa-miR156f-3p_L+1_1ss15T	TGCTCACTTCTCTTCCTGTCAGC	23	WXZB01002412.1	51.8	20	22
osa-miR156f-3p_L+1_1ss15T	TGCTCACTTCTCTTCCTGTCAGC	23	WXZB01576770.1	49.7	20	22
osa-MIR2118e-p3_2ss12TA18	AAAAGAGAGTCAAAGAGTA	19	WXZB01755556.1	34.5	20	16
osa-MIR2118e-p3_2ss13TA19	GAAAAGAGAGTCAAAGAGT	19	WXZB01037241.1	39.3	19	10
osa-MIR2118e-p3_2ss13TA19	GAAAAGAGAGTCAAAGAGTA	20	WXZB01075556.1	47.1	19	10
osa-MIR2118e-p3_2ss13TA19	GAAAAGAGAGTCAAAGAGTA	20	WXZB01075556.1	37.9	19	10
gma-MIR6300-p3_1ss18AG_2	CGTTGTAGTATAGTGGTGAGTATT	24	WXZB01234635.1	37.0	19	15
gma-MIR6300-p3_1ss18AG_1	CGTTGTAGTATAGTGGTGAGT	21	WXZB01437136.1	31.4	19	15
aof-MIR166b-p3_1ss18CT	TCGGACCAGGCTTCATTTTT	20	WXZB01000172.1	44.5	18	0
gma-miR172k	TGAATCTTGATGATGCTGCAT	21	WXZB01008427.1	46.5	18	13
gma-MIR5368-p3_1ss11CA	CCACTCTGGAAGAGCTAGAATTCT	24	WXZB01007556.1	48.9	18	3
osa-miR168a-3p_L-3_1ss19G	CCCGCCTTGCACCAACTGAAT	21	WXZB01013055.1	65.6	17	15
osa-miR168a-3p_L-3_1ss19G	CCCGCCTTGCACCAACTGAAT	21	WXZB01334153.1	66.3	17	15
osa-miR167a-5p	TGAAGCTGCCAGCATGATCTA	21	WXZB01007556.1	46.7	16	12
osa-MIR444e-p3_1ss12GA	CAAGTTATGCAATTGCTGCCT	21	WXZB01054022.1	30.3	16	9
osa-MIR444e-p5_1ss12GA	CAAGTTATGCAATTGCTGCCT	21	WXZB01303742.1	48.2	16	9

mtr-MIR2603-p5_2ss9AC17A(	GTCCCTGCCCTTTGTACA	18	WXZB01075556.1	55.8	16	7
mtr-MIR2603-p3_2ss9AC17A(	GTCCCTGCCCTTTGTACA	18	WXZB01550209.1	45.2	16	7
ptc-miR6478_R+1_1ss21GA	CCGACCTTAGCTCAGTTGGTAG	22	WXZB01755556.1	57.6	16	13
ptc-miR6478_R+1_1ss21GA	CCGACCTTAGCTCAGTTGGTAG	22	WXZB01755556.1	39.5	16	13
tae-miR319_R-1_1ss1TA	ATGGACTGAAGGGAGCTCCC	20	WXZB01075556.1	41.0	15	5
aof-miR319a_R+2_2ss19CT2	TTGGACTGAAGGGAGCTCTTTTT	23	WXZB01075556.2	40.3	15	27
gma-MIR5036-p5_1ss4AG	TTCGCTCTCCCTCAAGGGCTT	21	WXZB01280650.1	51.3	15	8
gma-MIR5036-p5_1ss4AG	TTCGCTCTCCCTCAAGGGCTT	21	WXZB01734495.1	53.1	15	8
gma-miR828a_R-1_1ss11AG	TCTTGCTCAAGTGAGTATTCC	21	WXZB01045954.1	51.4	14	22
aof-miR168a_R-1	TCGCTTGGTGCAGGTCGGGA	20	WXZB01007556.1	56.8	13	11
PC-3p-32831_31	TCATGCGGTTCCTTTGGAATT	21	WXZB01231374.1	40.1	13	18
PC-3p-39876_20	AGCTTCTCCACCATGCGCAAC	21	WXZB01075556.1	43.3	13	7
cpa-MIR159a-p5_1ss9TA	TTTGCATAACTCAGGAGCTGC	21	WXZB01001568.1	50.0	13	7
cpa-MIR159a-p5_1ss9TA	TTTGCATAACTCAGGAGCTGC	21	WXZB01282026.1	50.8	13	7
gma-MIR6300-p3	AGTATAGTGGTAAGTATTCCC	21	WXZB01007556.1	34.1	13	7
gma-MIR6300-p3	AGTATAGTGGTAAGTATTCCC	21	WXZB01444287.1	36.7	13	7
zma-miR162-5p_L+1_1ss10T	TGGGCGCAGCGGTTTATCGATC	22	WXZB01000756.1	50.3	12	8
mtr-MIR2592ay-p5_2ss6AG17	GAGTCGGGTTGTTTGGGA	18	WXZB01075556.1	47.1	12	9
mtr-MIR2592ay-p5_2ss6AG17	GAGTCGGGTTGTTTGGGAA	19	WXZB01075556.1	56.7	12	9
osa-miR5072_L-4_1ss12CT	TCCCCAGTGGAGTCGCCA	18	WXZB01000004.1	66.7	12	10
PC-5p-43270_17	CCAAATCGCGGCCGGAATCCG	21	WXZB01156592.1	64.3	12	5
osa-miR396e-5p_R+1_1ss21(	TCCACAGGCTTTCTTGAACTTT	22	WXZB01000076.1	43.7	11	21
osa-miR396e-5p_R+1_1ss21(	TCCACAGGCTTTCTTGAACTTT	22	WXZB01755556.1	40.4	11	21
ath-miR8175_L-2	TCCCCGGCAACGGCGCCA	18	WXZB01755556.2	50.3	10	5
PC-3p-41445_18	GTGCTGTTTCTCGTTGTCAAC	21	WXZB01007556.1	50.7	10	7
PC-3p-41445_18	GTGCTGTTTCTCGTTGTCAAC	21	WXZB01306906.1	54.4	10	7
sbi-MIR395d-p5_2ss5GT17TC	TAGATCTTTTGTGAAGGG	18	WXZB01075556.1	50.0	9	6
sbi-MIR395d-p5_2ss5GT17TC	TAGATCTTTTGTGAAGGG	18	WXZB01075556.1	48.4	9	6
vca-miR156b-3p	GCTCTCTATGCTTCTGTCATC	21	WXZB01007556.1	47.8	8	5
zma-miR164h-5p_1ss13GT	TGGAGAAGCAGGTCACGTGTG	21	WXZB01000614.1	52.4	8	9
ptc-miR6478_R+2_1ss21GA	CCGACCTTAGCTCAGTTGGTAGA	23	WXZB01075556.1	48.1	8	7
ptc-miR6478_R+2_1ss21GA	CCGACCTTAGCTCAGTTGGTAGA	23	WXZB01129759.1	41.9	8	7
gma-MIR10423-p3_1ss3AC	CTCTCAATGAAAGCACCA	18	WXZB01065113.1	35.6	8	0
gma-MIR10423-p5_1ss3AC	CTCTCAATGAAAGCACCA	18	WXZB01084591.1	39.8	8	0
PC-3p-36179_25	AACCGGCTGATGGACTTAAAGACC	24	WXZB01000519.1	35.9	8	11
PC-3p-36179_25	AACCGGCTGATGGACTTAAAGACC	24	WXZB01000756.1	37.9	8	11
PC-3p-47036_14	CGGGGGCAATGAATGGTT	18	WXZB01405944.1	47.3	8	6
osa-miR5072_L-4	TCCCCAGCGGAGTCGCCA	18	WXZB01075556.1	32.5	8	6
osa-miR5072_L-4	TCCCCAGCGGAGTCGCCA	18	WXZB01421997.1	56.5	8	6
gma-MIR9724b-p5_2ss12TC1	AGGGCTGGGCACGAGGGT	18	WXZB01075556.1	62.8	8	9
gma-MIR9724b-p5_2ss12TC1	AGGGCTGGGCACGAGGGT	18	WXZB01405807.1	67.4	8	9
vca-miR168b-3p_2ss3CT12C	CCTGCCTTGCATCAACTGAAT	21	WXZB01075556.1	65.3	7	0
sly-miR168a-3p	CCTGCCTTGCATCAACTGAAT	21	WXZB01176887.1	65.2	7	0
PC-3p-43946_16	CTCCCCTTCTCCAACTGAGGC	21	WXZB01169433.1	54.8	7	9
osa-MIR156i-p3_1ss16CT	TGCTCACTGCTCTGTTTGTCATC	23	WXZB01075556.1	58.0	7	4
cca-MIR6105b-p3_2ss8TG18	GTGAAGGGTTTGTGTTGG	18	WXZB01075556.1	50.0	7	3
gma-miR6300_R+5	GTCGTTGTAGTATAGTGGTGAGT	23	WXZB01075556.1	53.8	7	11
gma-miR6300_R+5	GTCGTTGTAGTATAGTGGTGAGT	23	WXZB01307476.1	33.7	7	11
gma-miR6300_L-1R+7	TCGTTGTAGTATAGTGGTGAGTAT	24	WXZB01490302.1	48.6	7	7
osa-miR156a_R+1	TGACAGAAGAGAGTGAGCACG	21	WXZB01167676.1	59.1	6	5
sbi-miR156a_R+1	TGACAGAAGAGAGTGAGCACG	21	WXZB01403991.1	57.0	6	5
PC-3p-37347_24	TGCTCACTCGTCTTCCTGTCAGC	23	WXZB01028565.1	48.4	6	6
PC-3p-37347_24	TGCTCACTCGTCTTCCTGTCAGC	23	WXZB01576770.1	49.7	6	6
osa-miR167a-5p_R-1	TGAAGCTGCCAGCATGATCT	20	WXZB01000411.1	44.4	6	3
osa-miR394_1ss19CT	TTGGCATTCTGTCCACCTTC	20	WXZB01007556.1	41.0	6	5

osa-miR394_1ss19CT	TTGGCATTCTGTCCACCTTC	20	WXZB01075556.1	37.6	6	5
peu-MIR2916-p3_2ss11TG17	AAGACGATCAGATACCGTCCTAGT(	25	WXZB01005846.1	51.2	6	8
gma-MIR6300-p5_1ss14AG	GTAGTATAGTGGTGAGTATTCCC	23	WXZB01755556.1	53.4	6	8
osa-miR535-5p_1ss21CA	TGACAACGAGAGAGAGCACGA	21	WXZB01214202.1	53.3	5	7
osa-miR535-5p_1ss21CA	TGACAACGAGAGAGAGCACGA	21	WXZB01367455.1	53.0	5	7
aof-miR167b_1ss2GC	TCAAGCTGCCAGCATGATCTGA	22	WXZB01367455.2	42.9	5	0
osa-miR166h-5p_1ss10GT	GGAATGTTGTCTGGCTCGAGG	21	WXZB01093229.1	47.5	4	0
osa-miR166b-5p_1ss19GA	GGAATGTTGTCTGGCTCGAGG	21	WXZB01122014.1	53.0	4	0
gma-miR6300_L-1R+8	TCGTTGTAGTATAGTGGTGAGTATT	25	WXZB01755556.1	43.2	4	4
osa-miR166g-3p_R-2	TCGGACCAGGCTTCATTCC	19	WXZB01122014.1	53.0	3	3
osa-miR166g-3p_R-2	TCGGACCAGGCTTCATTCC	19	WXZB01262413.1	45.1	3	3
sbi-MIR395d-p3_2ss5GT17T(	TAGATCTTTTGTGAAGGGT	19	WXZB01150637.1	48.2	3	4
gma-MIR168b-p5_1ss20GA	TGGTGCAGGTCGGGAACCGAT	21	WXZB01110970.1	59.3	3	3
ptc-miR6478_2ss6CT21GA	CCGACTTTAGCTCAGTTGGTA	21	WXZB01007556.1	52.2	3	4
ptc-miR6478_2ss6CT21GA	CCGACTTTAGCTCAGTTGGTA	21	WXZB01755556.1	28.7	3	4
csi-miR159b-5p	AGCTGCCGACTCATTCATTCA	21	WXZB01041941.1	45.4	0	3
csi-miR159b-5p	AGCTGCCGACTCATTCATTCA	21	WXZB01266896.1	48.4	0	3
ppe-miR396a_R-1	TTCCACAGCTTTCTTGAACG	20	WXZB01743445.1	43.5	0	6
ptc-miR6478_R-1_1ss6CT	CCGACTTTAGCTCAGTTGGT	20	WXZB01023161.1	53.3	0	5
ptc-miR6478_R+2_2ss6CT21	CCGACTTTAGCTCAGTTGGTAGA	23	WXZB01075556.1	52.2	0	5
ptc-miR6478_R+2_2ss6CT21	CCGACTTTAGCTCAGTTGGTAGA	23	WXZB01075556.1	53.4	0	5

*Each group was pooled from three independent samples.

Table S5: Primer sequences used for qPCR

Primers	Forward (5’-3’)	Reverse (5’-3’)
Tae-miR319	TGCTGCCTTAATGTCCAGTG	Universal primer (Qiagen)
Osa-miR166a-3p	TGGTGGACATCCTCATCCTT	Universal primer (Qiagen)
mND4	CCTACTCCTCCAATGGCAAA	CTCTTGCCCTTTCCTGCTTT
mND5	GCACCATGCCTAAAGCTGTC	ACTCAACACACCACCATTGC
mGAPDH	GGTCGGTGTGAACGGATTTG	GGAGTCATACTGGAACATGTAG
