# Supplementary material for: Plant-derived mitochondria mitigate aging-related neurodegeneration by reprogramming microglial mitochondrial energy metabolism
Source: Transl Neurodegener. 2026 Jul 8;15:30. doi: 10.1186/s40035-026-00565-1 (PMC13343874; doi:10.1186/s40035-026-00565-1)

‘supplementary file’ - full uncropped Gels and Blots image(s)

Fig. 1M

IDH

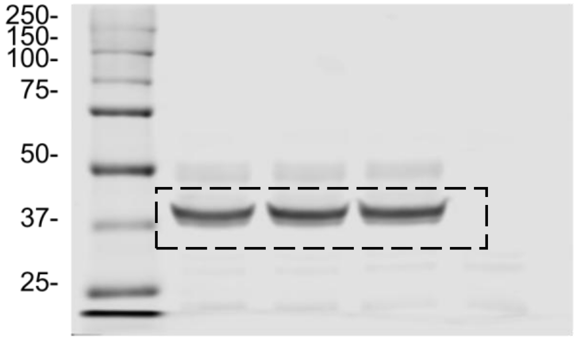

Fig. 3E

TREM2

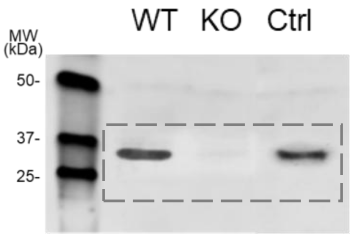

GAPDH

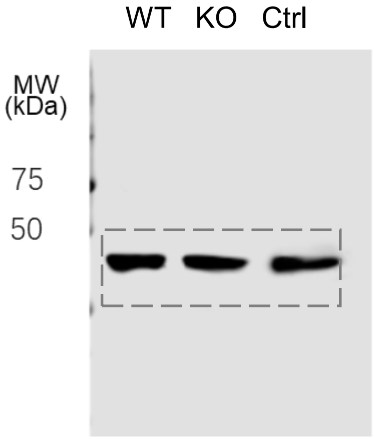

Fig. 3F

TREM2

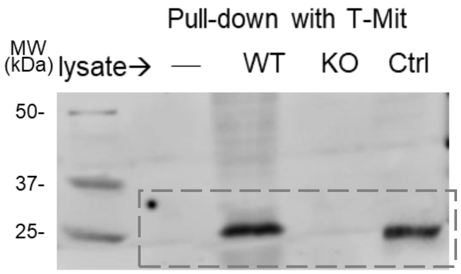

Fig. 5A

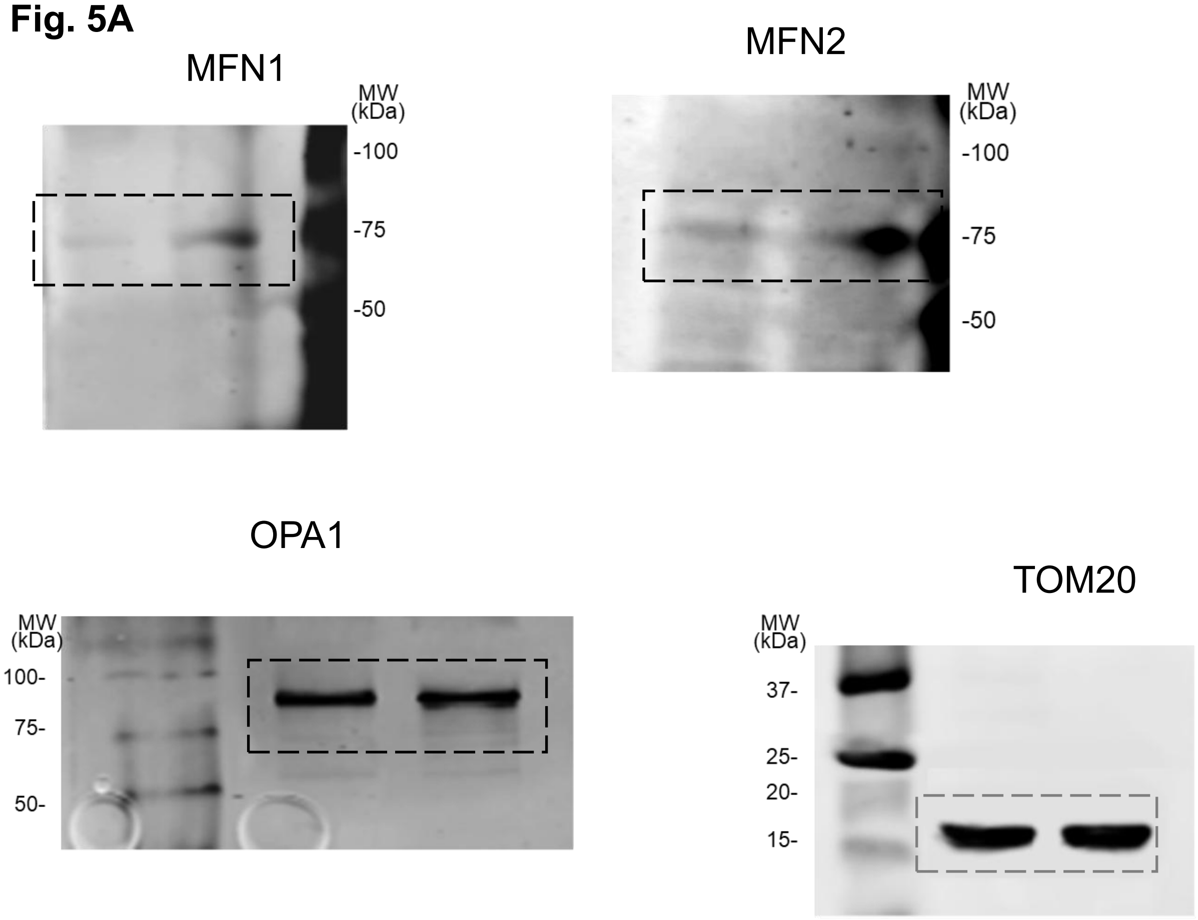
MFN1
MW (kDa)
-100
-75
-50
MFN2
MW (kDa)
-100
-75
-50
OPA1
MW (kDa)
100-
75-
50-
TOM20
MW (kDa)
37-
25-
20-
15-

Fig. 6L

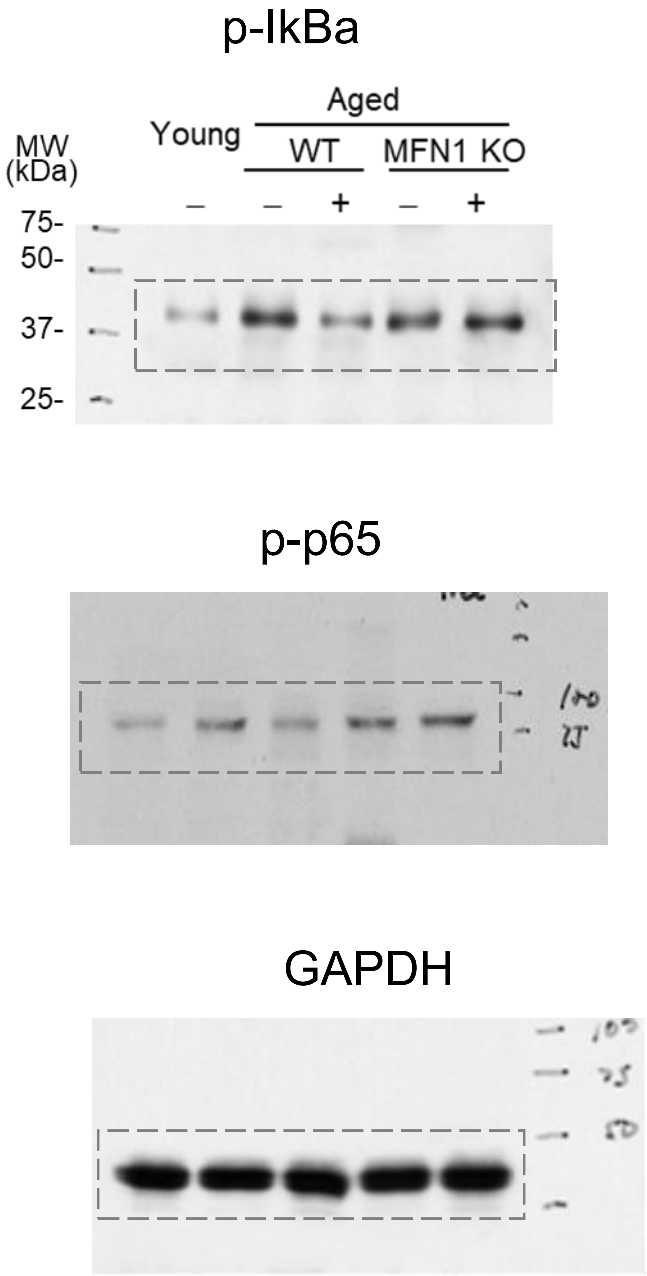

Figure 7D

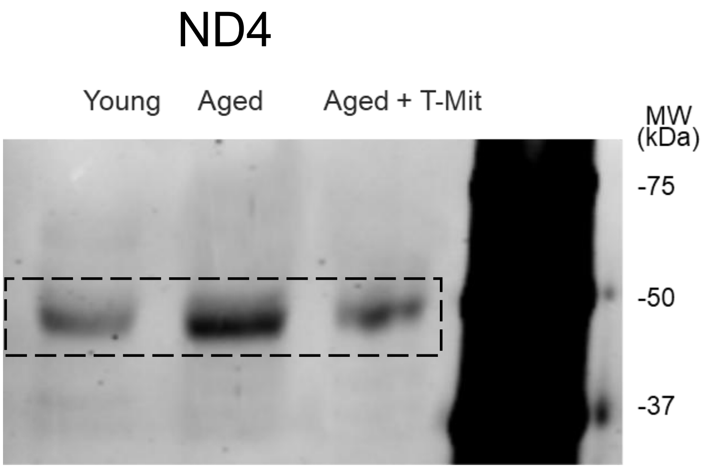
ND4
Young
Aged
Aged + T-Mit
MW (kDa)
-75
-50
-37

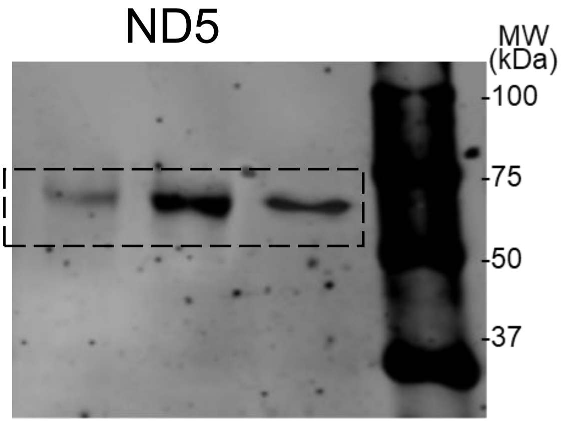
ND5
MW (kDa)
-100
-75
-50
-37

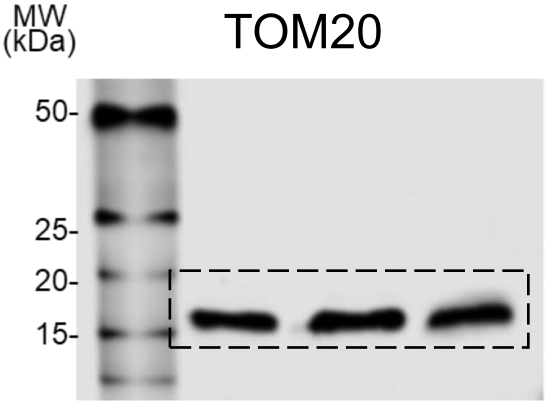
MW (kDa)
TOM20
50-
25-
20-
15-

Fig. 7F

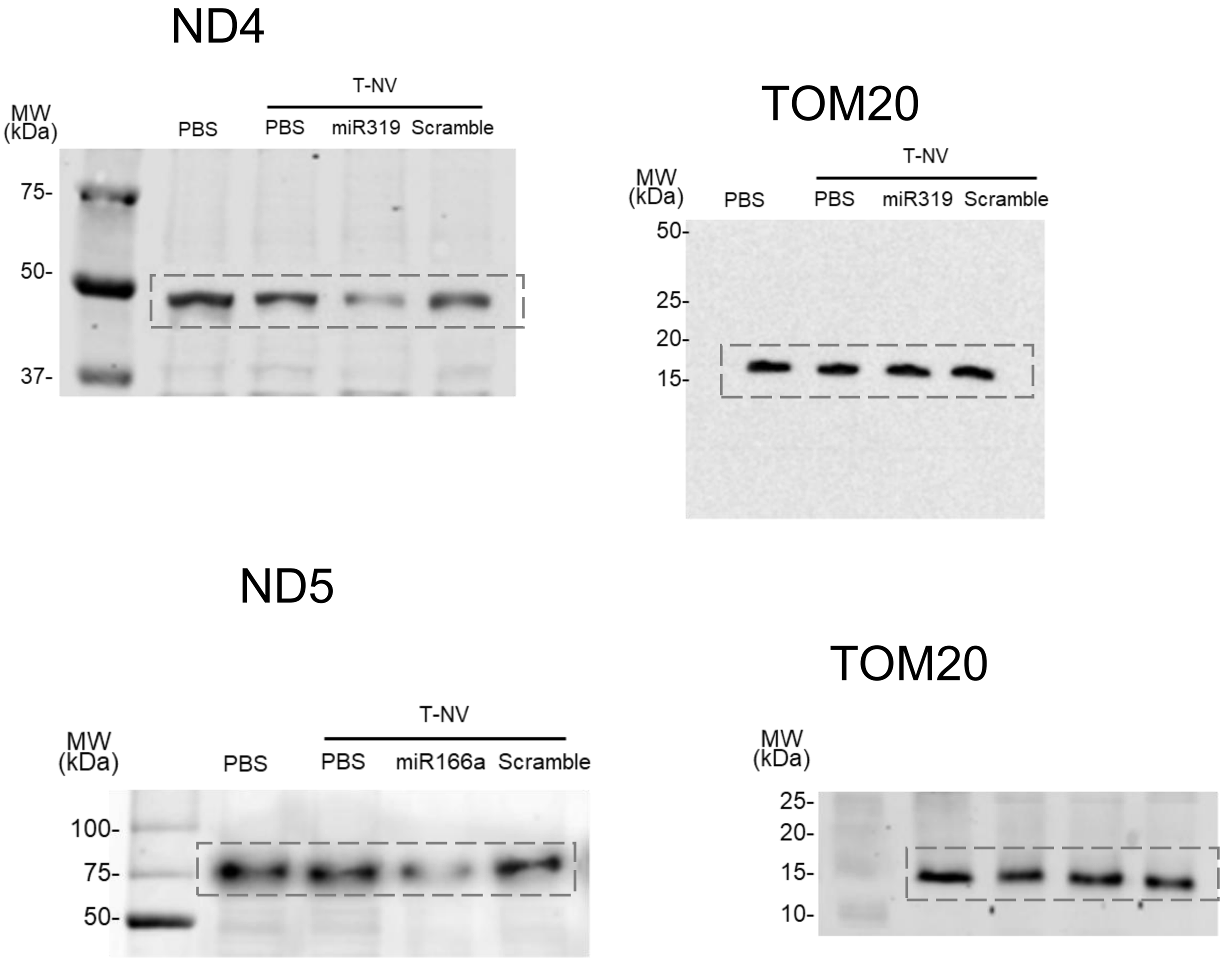
ND4
T-NV
MW (kDa)
PBS
PBS
miR319
Scramble
75-
50-
37-
TOM20
T-NV
MW (kDa)
PBS
PBS
miR319
Scramble
50-
25-
20-
15-
ND5
T-NV
MW (kDa)
PBS
PBS
miR166a
Scramble
100-
75-
50-
TOM20
MW (kDa)
25-
20-
15-
10-

Supplementary Figures

Fig. S1H

IDH

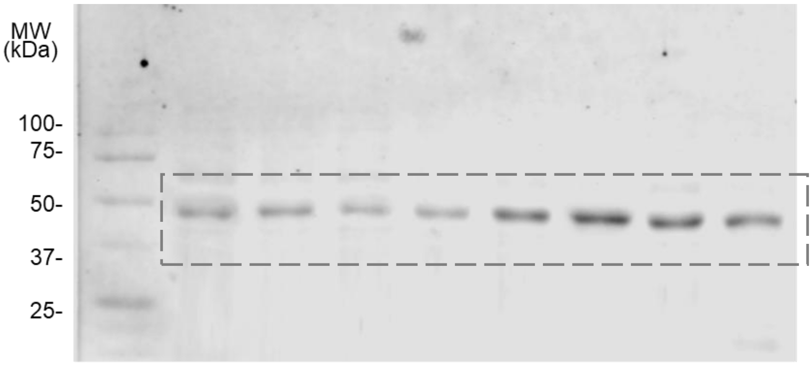

PATL

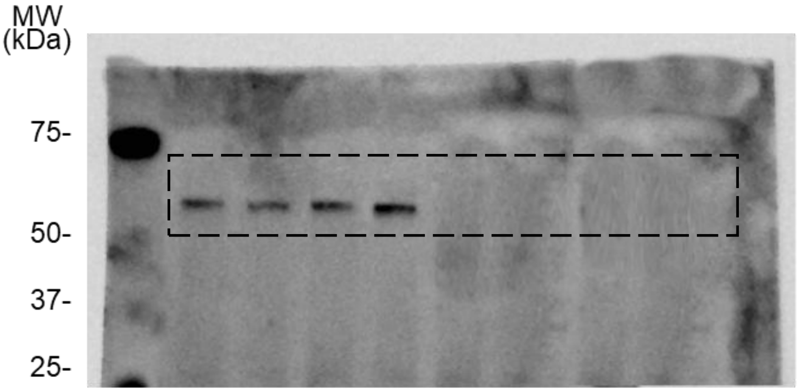

Fig. S5G

Western blot of TREM2 and GAPDH

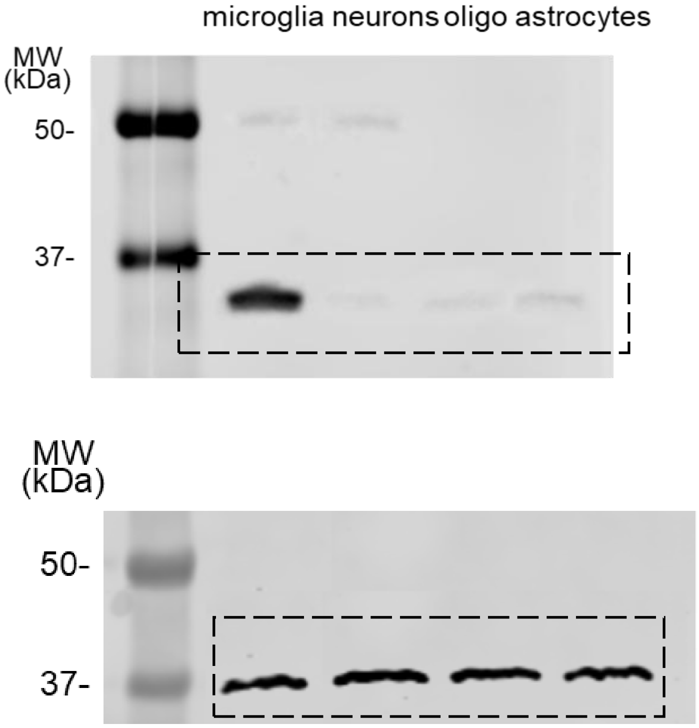

Fig. S6E

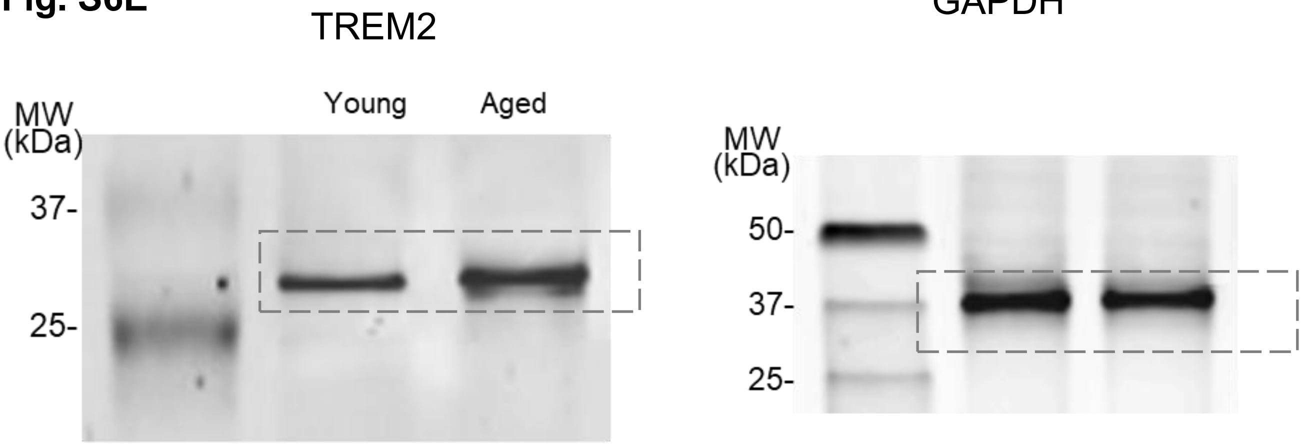

Figure S7F

IkBa

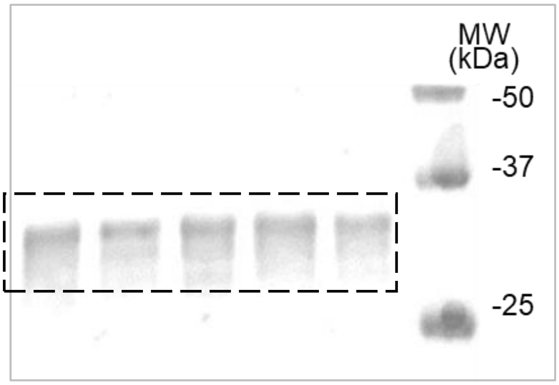

p65

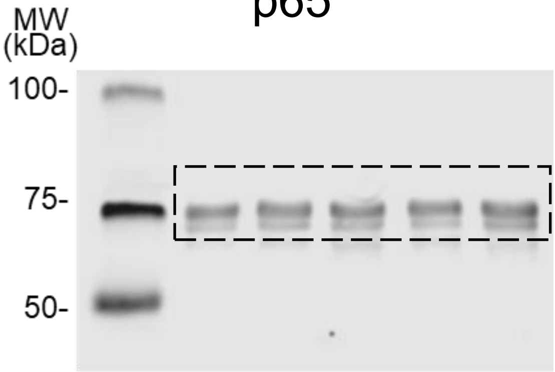

GAPDH

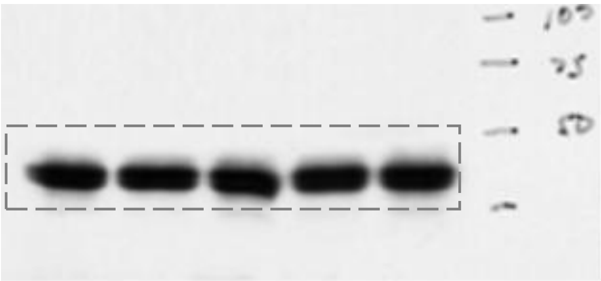

Supplement: Supplementary file 3 — Additional file 3. Full uncropped gels and blots. [file 40035_2026_565_MOESM3_ESM.pdf]
